# Efficacy and safety of immune checkpoint inhibitors for patients with prostate cancer: a systematic review and meta-analysis

**DOI:** 10.3389/fimmu.2023.1181051

**Published:** 2023-10-31

**Authors:** Maryam Noori, Shadi Azizi, Aref Mahjoubfar, Farhan Abbasi Varaki, Farimah Fayyaz, Amir-Hossein Mousavian, Davood Bashash, Mehdi Kardoust Parizi, Amir Kasaeian

**Affiliations:** ^1^ Student Research Committee, School of Medicine, Iran University of Medical Sciences, Tehran, Iran; ^2^ Hematology, Oncology and Stem Cell Transplantation Research Center, Research Institute for Oncology, Hematology and Cell Therapy, Tehran University of Medical Sciences, Tehran, Iran; ^3^ Colorectal Research Center, Iran University of Medical Sciences, Tehran, Iran; ^4^ Cancer Immunology Project (CIP), Universal Scientific Education and Research Network (USERN), Tehran, Iran; ^5^ Network of Immunity in Infection, Malignancy and Autoimmunity (NIIMA), Universal Scientific Education and Research Network (USERN), Tehran, Iran; ^6^ Digestive Diseases Research Center, Digestive Diseases Research Institute, Tehran University of Medical Sciences, Tehran, Iran; ^7^ Department of Hematology and Blood Banking, School of Allied Medical Sciences, Shahid Beheshti University of Medical Sciences, Tehran, Iran; ^8^ Department of Urology, Shariati Hospital, Tehran University of Medical Sciences, Tehran, Iran; ^9^ Department of Urology, Medical University of Vienna, Vienna, Austria; ^10^ Clinical Research Development Unit, Shariati Hospital, Tehran University of Medical Sciences, Tehran, Iran

**Keywords:** immune checkpoint inhibitors, prostate cancer, immunotherapy, PD-1, PDL, CTLA-4

## Abstract

Immunotherapy has revolutionized the treatment paradigm of many cancers, however, its effectiveness in prostate cancer patients is still under question. In the present systematic review and meta-analysis, we sought for assessing the efficacy and safety of Immune checkpoint inhibitors (ICIs) in patients with prostate cancer. PubMed, Scopus, Web of Science, and EMBASE databases were searched on Aguste 19, 2022. Thirty five studies met the eligibility criteria. The median overall survival (mOS) of all treatments was 14.1 months, with the longest and shortest mOS was seen among patients who received anti-CTLA-4 monotherapy and anti-PD-1/PD-L1+anti-CTLA-4 regimen at 24.9 and 9.2 months, respectively. Noteworthy, all types of adverse events had the lowest incidence in the anti-PD-1/PD-L1 monotherapy group. Considering the ICI monotherapy regimens, we found that fatigue, diarrhea, and infusion reaction had the highest incidence rates. Future studies evaluating the efficacy and safety of novel combination therapies with ICIs are warranted.

## 1 Introduction

With about 375 thousand deaths in 2020, prostate cancer is the fifth major cause of cancer death worldwide. Furthermore, approximately 1.5 million new cases were reported in 2020, which makes it the second most commonly diagnosed cancer (1). The majority of patients presented with localized disease which is mainly curable through radiation therapy, radical prostatectomy, or active surveillance (2). Patients who are diagnosed with metastatic hormone sensitive prostate cancer can be efficiently treated which led to a significant increase in their overall survival (3). Nevertheless, a number of patient specially those affected by castration-resistant prostate cancer progress to advanced metastatic disease which have poor outcomes. In such situation, the overall survival falls within nearly two years, highlighting the importance of finding more efficient treatments approaches (4, 5). Apart from chemotherapy and hormone therapies, radiopharmaceutical agents such as Radium-223 dichloride and 177-Lu-PSMA-617 were approved for the treatment of metastatic castration-resistant prostate cancer (6). However, there is still room for hope with more efficient medications.

Many trials using immunotherapy have been conducted for different types of tumors for over a decade, and they have expanded our knowledge of interactions between the immune system and diseases like cancer and its progression. Immunotherapy with immune checkpoint inhibitors (ICIs) has revolutionized the treatment paradigm of several tumors (7–10). In this case, cytotoxic T lymphocyte antigen 4 (CTLA-4), programmed-death 1 (PD-1), and programmed death-ligand 1 (PD-L1) inhibitors indicated promising results in melanoma, non-small cell lung cancer, and gastrointestinal cancers (11–13). In contrast, so-called “cold tumors”, such as prostate cancer, exhibit an immunosuppressive tumor microenvironment (TME) which results in a very restricted response to ICIs (14). Randomized controlled trials (RCTs) on patients with metastatic prostate cancer reported that using ICIs resulted in modest antitumor activity and suggested that combination therapy may enhance survival of these patients (10, 15, 16). Hence, in the present study, we systematically reviewed the clinical trials reporting the efficacy and safety of ICIs for patients with advanced prostate cancer and compared the finding of different regimens.

## 2 Methods

Present systematic review and meta-analysis was conducted in accordance with the Preferred Reporting Items for Systematic Reviews and Meta-Analyses (PRISMA) guidelines (17).

### 2.1 Search strategy

Eligible trials that evaluated the efficacy and safety of ICIs for patient suffering from prostate cancer were identified through a comprehensive literature search in PubMed, Scopus, Web of Science, and EMBASE databases. We searched the trials that were published in English as of Aguste 19, 2022, using the key terms including (“prostate neoplasm” OR “prostate cancer” OR “adenocarcinoma of prostate” OR “squamous cell carcinoma of prostate” OR “transitional cell carcinoma of prostate” OR “castration-resistant prostate neoplasm”) AND (“PD-L1 inhibitor” OR “PD-1 inhibitor” OR “CTLA-4 inhibitor” OR “Pembrolizumab” OR “Nivolumab” OR “Durvalumab” OR “Camrelizumab” OR “Atezolizumab” OR “Ipilimumab”) AND (“trial” OR “clinical trial”). The detailed information on search strategy was outlined in **Supplementary Table 1**. In addition, we reviewed the published abstracts from annual conferences of the American Society of Clinical Oncology (ASCO), the European Society of Medical Oncology (ESMO), and the American Association for Cancer Research (AACR). In the case where duplicate studies were identified, the most recent and complete version of the data was included.

### 2.2 Study selection

Obtained records were exported to EndNote software (Clarivate Analytics, Philadelphia, PA, USA). After removing the duplicate publications, two review authors independently reviewed the title/abstract of the articles according to the inclusion and exclusion criteria. Afterward, the same two authors screened the full-texts of the selected records, independently. Discrepancies were resolved by consulting a third author.

### 2.3 Eligibility criteria

Trials were included if the following criteria were met (1): patients with locally advanced or metastatic prostate adenocarcinoma aged 18 years or older were enrolled (2); a PD-1/PD-L1/CTLA-4 inhibitor with or without standard of care combination treatments was given to one of the study arms; and (3) outcomes of interest in terms of efficacy (i.e. overall survival [OS], progression-free survival [PFS], prostate specific antigen response [PSAR], objective response rate [ORR], disease control rate [DCR], complete response [CR], partial response [PR], stable disease [SD], or progressive disease [PD]) and safety (i.e. treatment-related adverse events (TRAEs), ≥ grade 3 TRAEs, immune-related adverse events (irAEs), serious adverse events (AEs), AEs led to treatment discontinuation, and AEs led to death) were reported.

The exclusion criteria were as follows (1): trials including a mixed cohort of patients with different cancer types (2); trials that administrated immunotherapeutic agents other than ICIs; and (3) other types of studies such as case reports, case series, case-controls, cohorts, cross-sectionals, editorials, letters to the editor, commentaries, re-analysis of previously published articles, and any types of review articles.

### 2.4 Data extraction

The following data were extracted by two authors independently from included trials (1): study characteristics including the name of the first author, year of publication or conference presentation, study title, clinical trial identification number, the acronym of the trial, country of origin, and phase of the trial (2); characteristics of participants including the total number of patients, inclusion and exclusion criteria, age, median follow up duration, PSA level (ng/ml), median duration of treatment, and Eastern Cooperative Oncology Group (ECOG) performance status scale (3); characteristics of intervention medications including type, dose, and schedule of ICI medication(s) along with concomitant treatment(s); and (4) efficacy and safety measures. Disagreements were addressed by consulting a third reviewer.

### 2.5 Quality assessment

All included studies were treated as non-randomized trials. Therefore, the Risk Of Bias In Non-randomized Studies of Interventions (ROBINS-I) tool was used for assessing the quality of trials (18). Two independent reviewers assessed the quality of the included papers. This tool examines the risk of bias according to the following domains: bias due to confounding, bias in selection of participants into the study, bias in classification of interventions, bias due to deviations from intended interventions, bias due to missing data, bias in measurement of outcomes, and bias in selection of the reported results.

### 2.6 Data synthesis

The primary efficacy endpoint was to estimate the median OS and PFS after receiving ICI treatment regimens and the secondary efficacy endpoint was to estimate the pooled rate of ORR, DCR, CR, PR, SD, and PD. The safety outcome was the pooled rate of TRAEs, ≥ grade 3 TRAEs, irAEs, serious AEs, AEs led to treatment discontinuation, and AEs led to death. We used Cochrane’s Q statistic to assess between-study heterogeneity and calculated the I-square statistic. A random-effect model was applied if obvious heterogeneity was present (*I* ^2^ >50%), otherwise, a fixed-effect model was chosen (19).

The subgroup analysis was conducted according to the target of ICI medication and the type of concomitant treatments. Differences between groups were tested by the chi-square test. The survival data were retrieved from Kaplan-Meyer curves via online plot digitizer tool (20). The pooled Kaplan-Meyer curves were plotted and analyzed using the package MetaSurvival (21) of software R version 3.6.3. Moreover, we used STATA version 17.0 (22) to calculate the pooled rates with *metaprop* command, which requires a nominator and a denominator (which is the total sample size) and some other options like random or fixed effects model. This command was built on the existing Stata command *metan*, which is routinely used to pool ratios and differences of means (23). A p-value less than 0.05 were treated as statistically significant.

## 3 Results

### 3.1 Study selection and characteristics of the included studies

**Figure 1:**
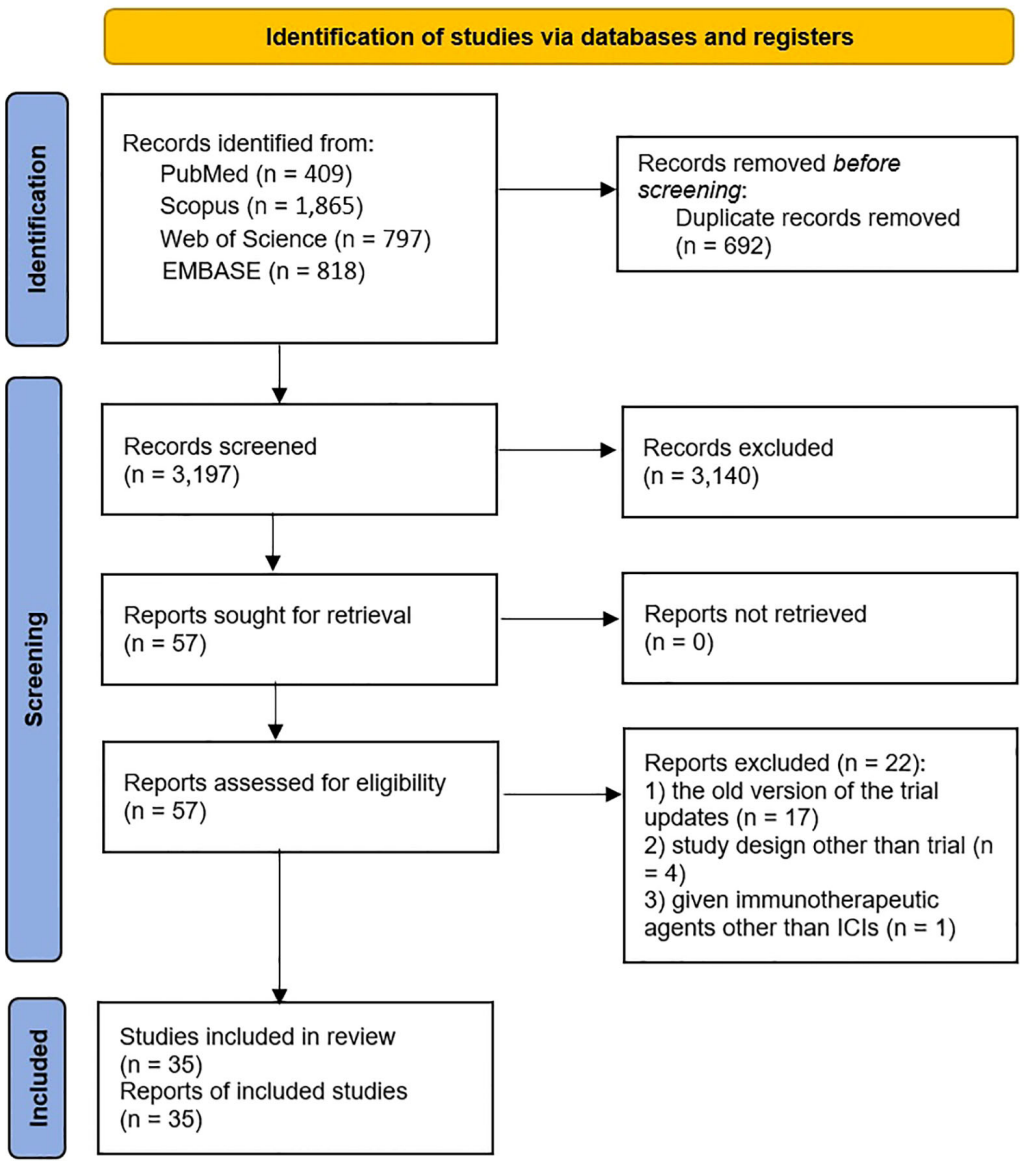
Study selection process of the meta-analysis according to the PRISMA flowchart.

As illustrated in the PRISMA flow diagram (**Figure 1**), 3,889 studies were identified through initial database searching. Following the removal of 692 duplicated records, the remaining 3,197 articles underwent title and abstract screening. After a detailed full-text evaluation of 57 potentially relevant studies, 22 studies were excluded, among which 17 studies were the old version of updated trials (24–40), four were not trials (41–44), and one article reported administration of an immunotherapeutic agent other than ICIs (45). Ultimately, 35 studies met the eligibility criteria and were included in the meta-analysis (10, 15, 16, 46–77).

The eligible articles were published between 2007 and 2022. All included trials were non-randomized studies except for three randomized trials (10, 15, 78) that we considered their experimental groups as single-arm trial. One study was in phase I, one in phase Ia, four in phase Ib, five in phase I/II, three in phase Ib/II, eighteen in phase II, and three in phase III. A total of 3,618 prostate cancer patients who received treatments comprised of ICIs were enrolled in the present meta-analysis. The treatment approaches in the eligible studies included anti PD-1/PD-L1+TKI (46), anti PD-1/PD-L1+radiotherapy (55, 62), anti PD-1/PD-L1+PARP inhibitor (54, 58, 61, 75), anti PD-1/PD-L1+hormone therapy (16, 56, 58, 63–65, 67, 74), anti PD-1/PD-L1+chemotherapy (53, 58, 68, 76), anti PD-1/PD-L1+anti CTLA-4 (47, 50, 60, 69, 70), anti PD-1/PD-L1+anti CTLA-4+hormone therapy (70), anti CTLA-4+radiotherapy (71), anti PD-1/PD-L1+anti CD-38 (77), anti PD-1/PD-L1 monotherapy (48, 49, 51, 52, 59, 60, 66), and anti CTLA-4 monotherapy (10, 15, 57, 71–73). The characteristics of the included studies and treatment doses and schedules are summarized in **Table 1** and **Supplementary Table 2**. In addition, the criteria of enrolling patients reported in the included studies are outlined in **Supplementary Table 3**.

**Table 1:** Characteristics of included studies.

First author	Year of publication	NCT identifier	Trial name	Phase	Total No of patients	Age	Median follow up duration	PSA level (ng/ml)	Median duration of treatment	ECOG PS	Treatment
**Agarwal et al.** (46)	2022	NCT03170960	COSMIC-021	1b	132	median [IQR] = 70 [64-75]	15·2 months	median [IQR] = 40·9 [9·5–89·9]	5·7 months	0 = 68 1 = 64	Atezolizumab + Cabozantinib
**Alva et al.** (47)	2022	NCT03570619	IMPACT (Cohort A)	2	28	NA	NA	2.31	NA	NA	Nivolumab and Ipilimumab
**Antonarakis** (1) **et al.** (48)	2019	NCT02787005	KEYNOTE-199	2	Cohort1 (PD-L1 positive): 133	median [range] = 68 [48-85]	9.5 months	median [range] = 115.5 [0.1-5000.0]	2.1 months	0 = 42 1 = 75 2 = 16	Pembrolizumab
					Cohort 2 (PD-L1 negative): 66	median [range] = 68 [53-84]	7.9 months	median [range] = 116.1 [1.0-3583.0]	1.6 months	0 = 25 1 = 36 2 = 4	
					Cohort 3 (bone predominant): 59	median [range] = 71 [53-90]	14.1 months	median [range] = 43.3 [0.1-2539.0]	3.2 months	0 = 26 1 = 27 2 = 6	
**Antonarakis** (2) **et al.** (49)	2021	NCT02787005	KEYNOTE-199	2	Cohort1 (PD-L1 positive): 133	NA	NA	NA	NA	NA	Pembrolizumab
					Cohort 2 (PD-L1 negative): 67						
					Cohort 3 (bone predominant): 58						
**Boudadi et al.** (50)	2018	NCT02601014	STARVE-PC	2	15	median [range] = 65 [52-76]	8.6 months	median [range] = 115 [31-7576]	NA	0 = 8 1 = 7	Nivolumab and Ipilimumab
**Brown et al.** (51)	2022	NCT03179410	PICK-NEPC	2	15	median [range] = 71 [51–85]	26 months	median [range] = 53.6 [0-393]	56 months	NA	Avelumab
**Fizazi** (1) **et al.** (54)	2021	NCT03338790	CheckMate 9KD	2	Cohort A1 (post-chemotherapy): 88	median [range] = 66 [46–85]	11.9 months	median [range] = 95.8 [0.1–4816.0]	4.4 months	0 = 39 1 = 48	Nivolumab+Rucaparib
					Cohort A2 (chemotherapy-naïve): 71	median [range] = 73 [51–87]	17.5 months	median [range] = 37.8 [0.6–5807.0]	5.8 months	0 = 30 1 = 41	
**Fizazi** (2) **et al.** (53)	2022	NCT03338790	CheckMate 9KD	2	84	median [range] = 71 [53-88]	15.2 months	median [range] = 49.5 [1.2-1085.0]	7.2 months	0 = 36 1 = 48	Nivolumab+Docetaxel
**Fakhrejahani et al.** (52)	2017	NCT01772004	JAVELIN	1	18	67	3 months	11	NA	NA	Avelumab
**Graff** (1) **et al.** (57)	2019	NCT01498978		2	10	median [range] = 64.5 [56–69]		NA	NA	0 = 7 1 = 3	Ipilimumab
**Fong et al.** (55)	2021	NCT02814669	BO30013	1b	44	median [range] = 69.0 [41–85]	13.9 months	median [range] = 51.6 [3–3051]	Atezolizumab: 97.9 days Radium-223: 83.9 days	0 = 26 1 = 18	Atezolizumab+Radium-223
**Graff** (2) **et al.** (56)	2020	NCT02312557	NA	2	28	median [range] = 72 [61-90]	37 months	median [range] = 26.61 [3.0–2502.7]	NA	0 = 11 1 = 17	Pembrolizumab+Enzalutamide
**Hansen et al.** (59)	2018	NCT02054806	KEYNOTE-028	1b	23	median [range] = 65 [46–83]	7.9 months	NA	NA	0 = 5 1 = 17 2 = 1	Pembrolizumab
**Hotte et al.** (60)	2019	NCT02788773	NA	2	39	median [range] = 70 [50-83]	NA	NA	NA	0 = 13 1 = 39	Durvalumab and Tremelimumab
					13						Durvalumab
**Howard et al.** (58)	2022	NCT02861573	KEYNOTE 365 K	1b/2	Cohort A: 102	NA	NA	NA	19.3 months	NA	Pembrolizumab+Olaparib
					Cohort B: 104				32.4 months		Pembrolizumab+Docetaxel
					Cohort C: 102				40.2 months		Pembrolizumab+Enzalutamide
**Karzai et al.** (61)	2018	NCT02484404	NA	2	17	median [range] = 66 [45–79]	9.7 months	median [range] = 79.7 [3.9–2356]		0 = 2 1 = 14 2 = 1	Durvalumab+Olaparib
**Kwan et al.** (62)	2021	ACTRN12618000954224	ICE-PAC	2	31	median [IQR] = 71 [64–75]	18.0 months	median [IQR] = 40 [13-118]	4.3 months	0 = 15 1 = 16	Avelumab+Stereotactic Ablative Body Radiotherapy (SABR)
**Kwon et al.** (10)	2014	NCT00861614	CA184-043	3	399	median [range] = 69 [47–86]	9·9 months	median [range] = 138·5 [0–4576]	119 days	0 = 168 1 = 216 2 = 3	Ipilimumab
**Layton et al.** (63)	2021	NCT03770455	NA	2	5	median [range] = 62 [54-73]	NA	median [range] = 7.2 [3.6-8.63]	NA	NA	Avelumab+Next generation hormonal therapies (NHTs)
**Markowski et al.** (64)	2021	NCT03554317	COMBAT-CRPC	2	45	69	NA	57.6	NA	0 = 12 1 = 23	Nivolumab+Testosterone cypionate(BAT)
**Mourey et al.** (65)	2020	NCT02861573	KEYNOTE-365 (Cohort C)	½	102	NA	19.1 months	NA	NA	NA	Pembrolizumab+Enzalutamide
**Petrylak et al.** (66)	2021	NCT01375842	PCD4989g	1a	35	median [range] = 68 [45–83]	13.0 months	121.4	2.1 months	0 = 12 1 = 23	Atezolizumab
**Piulats et al.** (67)	2021	NCT02861573	KEYNOTE 365 (cohort D)	1b/2	103	median [range] = 70 [46–89]	NA	NA	NA	NA	pembrolizumab+Abiraterone acetate
**Beer et al.** (15)	2017	NCT01057810		3	400	median [range] = 70 [44-91]	NA	median [range] = 41.2 [0.05-4956]		0 = 299 1 = 100 2 = 1	Ipilimumab
**Powles et al.** (16)	2022	NCT03016312	IMbassador250	3	379	median [range] = 70 [51–91]	15.2 month	NA	Atezolizumab: 3.5 months Enzalutamide: 4.5 months	NA	Atezolizumab +Enzalutamide
**Rodriguez-Vida et al.** (68)	2021	EudraCT 2017-004552-39		1b	26	median [range] = 70 [55-83]	14.2 months	NA	8.8 months	0 = 11 1 = 15	Avelumab +Carboplatin
**Sharma et al.** (69)	2020	NCT02985957	CheckMate 650	2	Cohort A1 (chemotherapy-naïve): 45	median [range] = 69 [48-85]	11.9 months	median [range] = 59.5 [3.3–1045.0]	2.1 months	0 = 26 1 = 19	Nivolumab and Ipilimumab
					Cohort A2 (postchemotherapy): 45	median [range] = 65 [46-84]	13.5 months	median [range] = 158.5 [1.8–1348.7]	1.4 months	0 = 25 1 = 20	
**Shenderov et al.** (70)	2021	NCT02601014	NA	2	15	median [range] = 65 [52-76]	9.9 months	median [range] = 115 [31–7576]	2.4 months	0 = 8 1 = 7	Nivolumab and Ipilimumab
					15	median [range] = 70.5 [54-77]		median [range] = 151.4 [4.4–1316.9]	2.8 months	0 = 10 1 = 5	Nivolumab and Ipilimumab+Enzalutamide
**Small et al.** (72)	2007	NA	NA	½	14	median [range] = 70.5 [56-79]	NA	median [range] = 84.6 [8-725]	NA	NA	Ipilimumab
**Subudhi et al.** (73)	2020	NCT02113657	NA	2	30	median [IQR] = 67 [58-73]	45.5 months	median [IQR] = 11.5 [1.7-91.2]	NA	NA	Ipilimumab
**Vaishampayan et al.** (74)	2020	NCT02787005	KEYNOTE-199	2	Cohort 4 (RECIST Measurable): 81	NA	15 months	NA	NA	NA	Pembrolizumab+ Enzalutamide
					Cohort 5 (Bone Predominant Nonmeasurable): 45		19 months				
**Yu** (1) **et al.** (75)	2021	NCT02861573	KEYNOTE-365 Cohort A	½	104	NA	19.3 months	NA	NA	NA	Pembrolizumab +Olaparib
**Slovin et al.** (71)	2013	NCT00323882		½	8	median [range] = 69 [55–78]	15.7 months	median [range] = 91 [7-449]	64 days	0 = 5 1 = 3 2 = 0	Ipilimumab
					7	median [range] = 68 [54–81]		median [range] = 47 [14-197]		0 = 4 1 = 2 2 = 1	Ipilimumab+External-beam radiotherapy (XRT)
					6	median [range] = 57 [51–68]		median [range] = 38 [3-111]		0 = 5 5 = 1 2 = 0	Ipilimumab
					16	median [range] = 65 [53–76]		median [range] = 132 [13-2581]		0 = 10 1 = 6 2 = 0	Ipilimumab
					34	median [range] = 66 [50–83]		median [range] = 120 [8-1314]		0 = 9 1 = 22 2 = 0	Ipilimumab+External-beam radiotherapy (XRT)
					50	median [range] = 65 [50–83]		median [range] = 133 [8-2581]		0 = 19 1 = 28 2 = 0	Ipilimumab+External-beam radiotherapy (XRT)
**Zucali et al.** (77)	2022	NCT03367819	NA	½	24	median [range] = 69.5 [61–88]			13.5 weeks	0 = 9 1 = 15	Cemiplimab+Isatuximab
**Yu** (2) **et al.** (76)	2022	NCT02861573	KEYNOTE-365 (cohort B)	1b/2	105	median [IQR] = 68 [64–74]	32.4 months	median [range] = 44.1 [17.1–131.4]	7.7 months	0 = 56 1 = 48	Pembrolizumab+Docetaxel

PSA, prostate specific antigen; ECOG PS, the Eastern Cooperative Oncology Group performance status; PD-L1, programmed death-ligand 1; IQR, interquartile range, NA, not available.

### 3.2 Quality assessment

According to the ROBINS-I tool, 23 trials were rated as having a moderate methodological quality. In addition, no precise information was reported in 12 trials for assessing the risk of bias. Bias due to confounding was the domain that had the highest rate of moderate risk of bias items, whereas bias due to deviations from intended Intervention was the domain that had the highest rate of low risk of bias items **Supplementary Figure 1**.

### 3.3 Efficacy

#### 3.3.1 Overall survival

**Figure 2:**
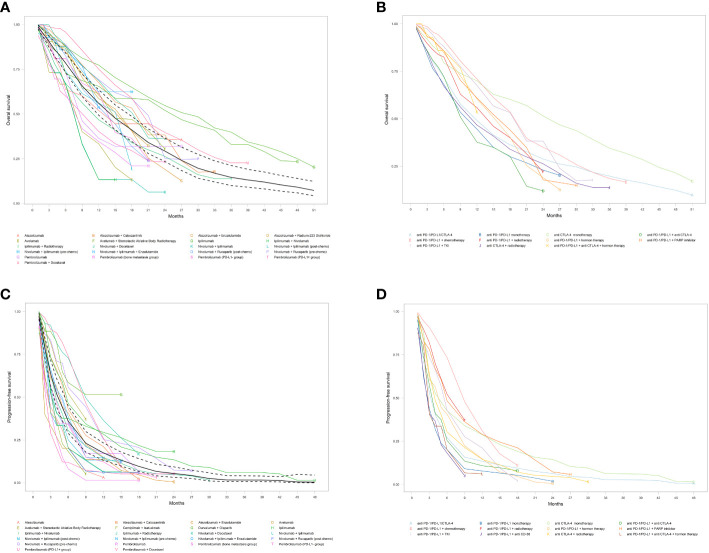
Pooled Kaplan-Meier estimates of overall survival (OS) and progression-free survival (PFS). **(A)** Overall OS; **(B)** OS subgroups by different immune checkpoint inhibitor treatment regimen; **(C)** Overall PFS; and **(D)** PFS by different immune checkpoint inhibitor treatment regimen. Solid and dashed black lines indicate pooled overall estimate and its 95% confidence intervals, respectively.

The pooled overall OS among all patients receiving ICI monotherapy or in combination with other therapies was calculated, and the Kaplan Meier curve was built (**Table 2** and **Figure 2A**). The mOS was 14.1 months (95% CI: 11.7-17.0), and the 6-, 12-, 18-, and 24-months OS rates were 78.4%, 56.0%, 41.0%, and 29.2%, respectively.

**Table 2:** Pooled results of overall survival and progression-free survival in total and by immune checkpoint inhibitor treatment regimen subgroups.

	Pooled mOS (95% CI), months	Pooled 6-m OS (95% CI), %	Pooled 12-m OS (95% CI), %	Pooled 18-m OS (95% CI), %	Pooled 24-m OS (95% CI), %	Pooled mPFS (95% CI), months	Pooled 6-m PFS (95% CI), %	Pooled 12-m PFS (95% CI), %	Pooled 18-m PFS (95% CI), %	Pooled 24-m PFS (95% CI), %
**All drugs combined**	14.1 (11.7- 17.0)	78.4 (73.2-84.1)	56.0 (49.3-63.6)	41.0 (34.6-48.6)	29.2 (23.4-36.5)	4.3 (3.5-5.3)	35.9 (28.9-44.8)	17.5 (13.1-23.2)	9.6 (6.5-14.1)	5.4 (3.2-9.3)
**anti PD-1/PD-L1/CTLA-4**	11.7 (8.2-17.2)	71.8 (62.8-82.0)	49.2 (37.8-64.0)	36.3 (26.0-50.7)	28.8 (19.9-41.7)	3.2 (2.4-4.5)	27.1 (18.3-40.3)	13.5 (7.7-23.4)	9.5 (5.6-16.1)	6.4 (3.4-11.9)
**anti PD-1/PD-L1 monotherapy**	10.2 (7.6-13.0)	68.2 (59.7-77.8)	44.6 (34.8-57.0)	30.1 (22.4-40.3)	23.0 (15.8-33.5)	2.7 (1.9-3.7)	20.2 (10.9-37.3)	7.8 (3.4-17.8)	5.3 (2.3-12.4)	1.9 (0.1-36.9)
**anti CTLA-4 monotherapy**	24.9 (11.4-30.5)	79.4 (62.4-101.0)	70.0 (54.7-89.6)	60.2 (48.8-74.3)	51.5 (42.2-62.7)	4.5 (2.6-6.0)	42.6 (31.5-57.7)	28.8 (23.9-34.7)	19.2 (15.2-24.1)	14.5 (11-19.2)
**anti PD-1/PD-L1+anti CTLA-4**	9.2 (NA-NA)	72.3 (58.7-89.1)	37.7 (20.3-69.9)	30.2 (15.0-60.9)	12.0 (1.7-86.9)	3.4 (2.8-4.6)	25.4 (16.3-39.8)	10.9 (5.2-22.6)	8 (2.7-23.9)	NA
**anti PD-1/PD-L1+chemotherapy**	18.8 (14.8-21.1)	91.3 (83.4-100.0)	71.0 (62.4-80.7)	53.6 (43.6-65.7)	34.9 (25.4-48.0)	8.7 (7.6-10.0)	73.4 (66.8-80.6)	31 (20.2-47.5)	11.1 (3.8-32.3)	NA
**anti PD-1/PD-L1+radiotherapy**	13.8 (3.9-10.5)	82.6 (72.4-94.2)	55.9 (42.2-73.9)	40.0 (26.2-61.1)	22.1 (8.1-60.2)	8.4 (4.5-NA)	52.2	NA	NA	NA
**anti PD-1/PD-L1+hormon therapy**	15.2 (14.0-17.0)	84.7	60.5	40.3	19.0	4.2 (4.1-5.3)	40.7	14.8	4.7	0.6
**anti PD-1/PD-L1+PARP inhibitor**	15.8 (NA-NA)	85.8 (77.9-94.4)	61.0 (46.3-80.2)	44.1 (24.1-80.8)	17.8 (6.3-50.8)	5.9 (3.9-10.5)	48.4 (33-70.9)	28.9 (16-52.3)	21.2 (9.7-46.3)	7.3 (1.1-46.6)
**anti PD-1/PD-L1+TKI**	18.4 (14.3-24.7)	42.9	21.1	2.2	NA	5.5 (4.3-6.6)	88.1	67.6	53.2	38.2
**anti PD-1/PD-L1+anti CD-38**	NA	NA	NA	NA	NA	2.3 (1.9 to 4.3)	18.2	NA	NA	NA
**anti CTLA-4+radiotherapy**	11.2 (9·.5-12.7)	67.2	46.7	33.9	26.3	4·0 (3·6-4·3)	30.4	14.6	7.1	4.8
**anti PD-1/PD-L1+anti CTLA-4+hormon therapy**	14.2 (8.5-NA)	85.8	53.5	NA	NA	2.9 (1.3-5.8)	20.2	6.2	NA	NA

PD-1, programmed death 1; PD-L1, programmed death-ligand 1; CTLA-4, cytotoxic T-lymphocyte antigen 4; PARP, poly-ADP ribose polymerase; TKI, tyrosine kinase inhibitors; OS, overall survival; PFS, progression-free survival; CI, confidence interval; NA, not available.

Subgroup analysis among different ICI regimens demonstrated that anti-CTLA-4 monotherapy had by far the longest mOS at 24.9 months (95% CI: 11.4-30.5), followed by anti-PD-1/PD-L1+chemotherapy regimen at 18.8 months (95% CI: 14.8-21.1), and anti-PD-1/PD-L1+TKI group at 18.4 months (95% CI: 14.3-24.7). Patients in the anti-PD-1/PD-L1+PARP, anti-PD-1/PD-L1+hormone therapy, anti-PD-1/PD-L1+anti-CTLA-4+hormone therapy, and anti-PD-1/PD-L1+radiotherapy groups ended up with similar mOS of 15.8 (95% CI: NA) and 15.2 (95% CI: 14.0-17.0), 14.2 (95% CI: 8.5-NA), and 13.8 (95% CI: NA) months, respectively. On the contrary, the anti-PD-1/PD-L1+anti-CTLA-4 regimen had the poorest mOS, with just 9.2 months (95% CI: NA), inferior to anti-PD-1/PD-L1/CTLA-4 (11.7 months, 95% CI: 8.2-17.2), anti-CTLA-4+radiotherapy (11.2 months, 95% CI: 9.5-12.7), and anti-PD-1/PD-L1 monotherapy (10.2 months, 95% CI: 7.6-13.0). The mOS and OS rates for different ICI monotherapy and in combination with other therapies and their Kaplan-Meyer curve are represented in **Table 2** and **Figure 2B**.

#### 3.3.2 Progression-free survival

The pooled mPFS was 4.3 months (95% CI: 3.5-5.3) with 6-, 12-, 18-, and 24-months PFS rates of 35.9%, 17.5%, 9.6%, and 5.4%, respectively (**Table 2** and **Figure 2C**). The evaluation of PFS in different ICI regimens showed that the longest mPFS was observed in the anti-PD-1/PD-L1+chemotherapy regimen at 8.7 months (95% CI: 7.6-10.0). The pooled mPFS among patients receiving anti-PD-1/PD-L1+radiotherapy was 8.4 months (95% CI: 4.5-NA). For patients taking anti-PD-1/PD-L1+PARP inhibitor, the mPFS was 5.9 months (95% CI: 3.9-10.5) and for those taking anti PD-1/PD-L1+TKI, the mPFS was 5.5 months (95% CI: 4.3-6.6). The anti-CTLA-4 monotherapy, anti-PD-1/PD-L1+hormone therapy, and anti-CTLA-4+radiotherapy groups ended up with comparable mPFS of 4.5 (95% CI: 2.6-6.0), 4.2 (95% CI: 4.1-5.3), and 4.0 months (95% CI: 3.6-4.3), respectively. The shortest mPFS belonged to the anti-PD-1/PD-L1+anti CD-38 group at 2.3 months (95% CI: 1.9-4.3), marginally lower than anti-PD-1/PD-L1+anti CTLA-4, anti-PD-1/PD-L1/CTLA-4, anti-PD-1/PD-L1+anti CTLA-4+hormone therapy, and anti-PD-1/PD-L1 monotherapy, at 3.4 (95% CI: 2.8-4.6), 3.2 (95% CI: 2.4-4.5), 2.9 (95% CI: 1.3-5.8), and 2.7 months (95% CI: 1.9-3.7), respectively. The detailed results of PFS among different ICI regimen groups and their Kaplan-Meyer curve are presented in **Table 2** and **Figure 2D**.

#### 3.3.3 Response rates

**Figure 3:**
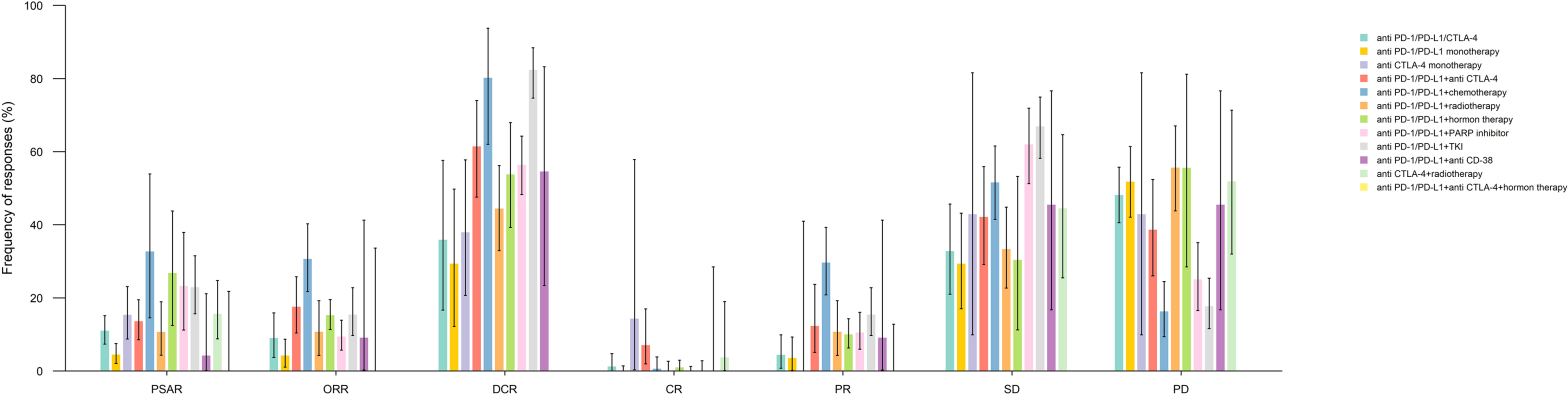
Pooled response rates for prostate specific antigen response (PSAR), objective response rate (ORR), disease control rate (DCR), complete response (CR), partial response (PR), stable disease (SD), and progressive disease (PD) by immune checkpoint inhibitor medication subgroups.

The ORR, DCR, and PSAR rates were analyzed according to the ICI therapy regimen type. The bar chart illustrated in **Figure 3** describes the aforementioned response rates between different treatment regimens containing ICIs. **Supplementary Table 4** and **Supplementary Figure 2** show that the pooled PSAR rates in anti-PD-1/PD-L1+chemotherapy and anti-PD-1/PD-L1+hormone therapy groups were significantly higher than in other ICI regimens groups, at 32.68% (95% CI: 14.52-53.90) and 26.76% (95% CI: 12.43-43.76), respectively. In contrast, the lowest PSAR rates were at 0.0%, 4.17%, and 4.46% among patients receiving anti-PD-1/PD-L1+anti-CTLA-4+hormone therapy, anti-PD-1/PD-L1+anti-CD38, and anti-PD-1/PD-L1 monotherapy, respectively. The PSAR in anti-PD-1/PD-L1 monotherapy was considerably inferior to almost all other ICI regimen groups. Moreover, the pooled PSARs among patients receiving anti PD-1/PD-L1/CTLA-4, anti CTLA-4 monotherapy, anti PD-1/PD-L1+anti CTLA-4, anti PD-1/PD-L1+radiotherapy, and anti PD-1/PD-L1+PARP inhibitor were 10.98%, 15.32%, 13.60%, 10.61%, and 23.24%, respectively. Significant heterogeneity was observed in pooled PSAR rate analyses of anti-PD-1/PD-L1/CTLA-4 and anti-PD-1/PD-L1+hormone therapy (I^2 = ^44.12%, p=0.04 and I^2 = ^89.03%, p<0.01, respectively).

The highest pooled ORR was observed in the anti-PD-1/PD-L1+chemotherapy group at 30.61% (95% CI: 21.71-40.28), which was significantly higher than in other ICI therapy groups, except in anti-PD-1/PD-L1+anti CD-38 group (9.09%, 95% CI: 0.23-41.28) (**Supplementary Table 5**). Comparatively, patients taking anti-PD-1/PD-L1 monotherapy and anti-PD-1/PD-L1+anti-CTLA-4+hormone therapy had ORRs of 4.20% (95% CI: 1.06-8.67) and 0% (95% CI: 0-33.63), significantly lower than in anti-PD-1/PD-L1+anti CTLA-4 (17.51%), anti-PD-1/PD-L1+chemotherapy (30.61%), anti-PD-1/PD-L1+hormone therapy (15.23%), and anti-PD-1/PD-L1+TKI (15.38%) groups. Moreover, patients receiving anti-PD-1/PD-L1/CTLA-4 (9.01%), anti-PD-1/PD-L1+PARP inhibitor (9.41%), and anti-PD-1/PD-L1+ radiotherapy (10.67%) had comparable ORRs. The ORRs reported in studies evaluating the anti-PD-1/PD-L1/CTLA-4 receiving patients had significant heterogeneity (I^2 = ^58.86, p=0.01) (**Supplementary Figure 3**).

Although similar to ORR, the DCR observed in the anti-PD-1/PD-L1+chemotherapy group was considerably high (80.21%, 95% CI: 61.98-93.77), the highest DCR was detected among the anti-PD-1/PD-L1+TKI group (82.31%, 95% CI: 74.65-88.44). On the contrary, the anti-PD-1/PD-L1 monotherapy group had the lowest DCR, at 29.31% (95% CI: 12.18-49.78), which was significantly lower than that in all the other ICI regimen groups. Likewise, patients taking the anti-PD-1/PD-L1/CTLA-4 regimen also had a DCR of 35.86% (95% CI: 16.61-57.60), significantly lower than in other regimen groups, except in the anti-CTLA-4 monotherapy group (37.93%, 95% CI: 20.69-57.74). Moreover, the DCR in the anti-PD-1/PD-L1+radiotherapy (44.41%), anti-PD-1/PD-L1+hormone therapy (53.77%), anti-PD-1/PD-L1+CD-38 (54.55%), anti-PD-1/PD-L1+PARP inhibitor (56.34), and anti-PD-1/PD-L1+anti-CTLA-4 (61.4%) groups were alike. Considerable heterogeneity was detected in the pooled analysis of DCR in the anti-PD-1/PD-L1/CTLA-4 (I^2^ = 92.09%, p<0.01), anti-PD-1/PD-L1 monotherapy (I^2^ = 83.93%, p<0.01), and anti-PD-1/PD-L1+hormone therapy (I^2^ = 72.71%, p=0.01) (**Supplementary Figure 4** and **Supplementary Table 6**).

The pooled results of CR, PR, SD, and PD by ICI treatment regimen groups, as well as the tables of associated p-values, are presented in **Supplementary Figures 5**-**8** and **Supplementary Tables 7**-**10**.

### 3.4 Safety

**Figure 4:**
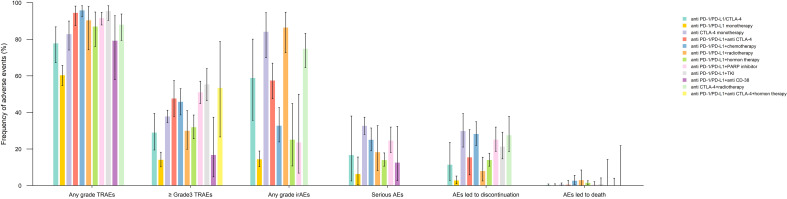
Pooled frequency of treatment-related adverse events (TRAEs), ≥ grade 3 TRAEs, immune-related adverse events (irAEs), serious adverse events (AEs), AEs led to treatment discontinuation, and AEs led to death.

Any grade TRAEs, ≥ grade3 AEs, irAEs, serious AEs, AEs led to discontinuation, and AEs led to death were analyzed according to the ICI therapy regimen subgroup. The bar chart presented in **Figure 4** shows the earlier mentioned AEs between different treatment regimens containing ICIs. Noteworthy, all the AEs mentioned before had the lowest incidence in the anti-PD-1/PD-L1 monotherapy group. The anti-PD-1/PD-L1+TKI and anti-PD-1/PD-L1+chemotherapy had the highest incidence of ≥ grade3 and any grade TRAEs, respectively. The anti-PD-1/PD-L1+radiotherapy group had the highest any grade irAEs, and AEs led to death, whereas anti-CTLA-4 monotherapy was the leading subgroup in serious AEs and AEs led to discontinuation. The detailed pooled results of different AE types by ICI medication subgroup and their associated p-values are manifested in **Supplementary Figures 9**-**14** and **Supplementary Tables 11**-**16**.

**Figure 5:**
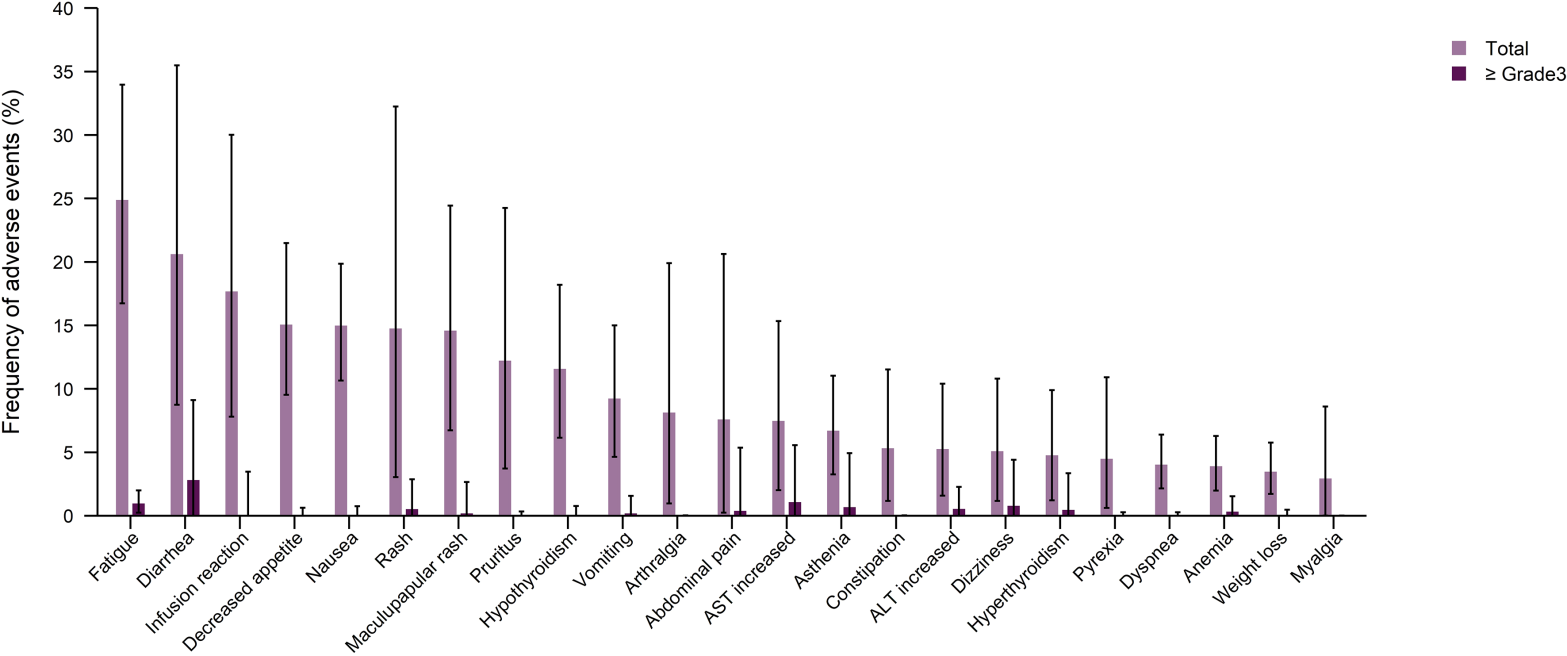
Pooled frequency of any grade treatment-related adverse events (TRAEs) and ≥ grade 3 TRAEs caused by immune checkpoint inhibitor monotherapy treatment regimen groups.

To eliminate the AEs related to components of treatment other than ICIs, an additional analysis was carried out to determine the frequency of TRAEs in treatment regimens consisting of ICIs alone (anti-PD-1/PD-L1+/anti CTLA-4). The most commonly reported any grade TRAEs included fatigue (24.88%), diarrhea (20.61%), infusion reaction (17.67%), and decreased appetite (15.06%). Moreover, the most frequent ≥ grade 3 TRAE was diarrhea (2.8%), followed by AST increase (11.07%), fatigue (0.97%), and dizziness (0.79%) (**Figure 5** and **Supplementary Figures 15**, **16**).

## 4 Discussion

ICI medications have been long investigated for urological cancers, leading to FDA approval of Nivolumab, Pembrolizumab, and Avelumab for urothelial carcinoma (79). Likewise, Ipilimumab, Nivolumab, Pembrolizumab, and Avelumab gained FDA approval for advanced renal cell carcinoma, mainly in combination with other medications in the class TKIs such as Axitinib, Cabozantinib, or Lenvatinib (79). Nevertheless, the benefit of ICIs in prostate cancer remain unclear (80). There has been several single-arm trials on efficacy and safety of ICIs as monotherapy or in combinations with other therapeutic modalities including androgen inhibitors, PARP inhibitors, TKIs, chemotherapy, radiotherapy, or other ICIs (81, 82). However, only the results of 3 phase III trials using the Ipilimumab or Atezolizumab have been published for mCRPC patients (10, 15, 16); and due to non-promising findings, none of ICIs have been approved by FDA and fit the treatment paradigm for prostate cancer patients.

The present systematic review is the first study aiming at pooling the results of clinical trials administrating ICIs for prostate cancer patients. We compared the effectiveness and AEs of several ICI monotherapy and combination therapy regimens in order to be used in clinical practice. Notably, anti-PD-1/PD-L1 monotherapy or combination of anti-PD-1/PD-L1 with CTLA-4 inhibitors showed 4.2% and 17.5% ORR in our study, respectively. In agreement, the results of a recent meta-analysis showed that Pembrolizumab monotherapy or Ipilimumab-plus-Nivolumab regimen resulted in an ORR of 5% and 17%, among prostate cancer patients, respectively (83). Regarding PSAR, similar results were obtained; while the PSAR for Pembrolizumab monotherapy, Ipilimumab monotherapy, and Ipilimumab-plus-Nivolumab were 11%, 19%, and 14%, respectively in the mentioned study (83), it was 4.5%, 15.3%, and 13.6% for anti-PD-1/PD-L1 monotherapy, anti-CTLA-4 monotherapy, and anti-PD-1/PD-L1+anti-CTLA-4 regimen in accordance to our findings, respectively.

Although initial studies have proposed that immunotherapy with an autologous active cellular vaccine (Sipuleucel-T) provide promising outcomes for men with prostate cancer (84), the body of evidence came into the conclusion that prostate tumor is less immunogenic than thought before (85–87). Immunologically, prostate cancer is a cold tumor which represents with low tumor mutational burden (TMB) (73, 88). It has been reported that efficacy of ICIs for prostate cancer patients outstripped the chemotherapy only when the TMB exceeded 10 mutations per megabase, detected only in a low proportion of patients (42). Low level of TMB and subsequently lower level of neoantigen expression reflects decreasing rate of immune cell infiltration particularly T cells into the prostate tumor tissue. Additionally, the hypoxic zone of the prostate tumor microenvironment prohibits T-cell attraction by causing an acidic pH, depleting important nutrients, promoting transforming growth factor-Beta (TGF-B) signaling, and activating myeloid-derived suppressor cells (89, 90). Thus, by inhibition of CD8^+^ T cell infiltration, a compromised response to ICIs would be observed in prostate cancer patients.

In order to combat the immunosuppressive microenvironment and override tumor escape mechanisms, multiple approaches have been examined to improve the efficacy of ICIs for prostate cancer patients. According to our study, a number of trials attempted to enhance the chance of better outcomes following ICI therapy by combining it with other medications. In this case, we found that administration of ICIs with chemotherapy regimens had desirable outcomes in a way presenting with the longest PFS as well as highest ORR and PSAR among prostate cancer patients. Indeed, killing tumor cells by chemotherapeutic agents can result in releasing tumor neoantigens, overcoming compensatory immunosuppressive mechanisms, and subsequently improving the function of effector immune cells (91–93). Therefore, combining immunotherapy with chemotherapy can induce addictive or synergic clinical activity. Apart from chemotherapy, studies have shown that a combination of CTLA-4 and PD-1/PD-L1 blockade has better antitumor outcome than monotherapy regimens alone (94); however, our results showed that anti-PD-1/PD-L1+anti-CTLA-4 regimen had the lowest mOS among all other regimens. This might be due to the intrinsic characteristics of the enrolled patients in trials that were associated with worse outcomes and need to be investigated further in future trials.

It has been reported that ICIs, in spite of their efficacies, may induce a wide range of AEs which should be monitored closely. In the present review, we assessed the safety of ICIs as well as the severity of side effects in prostate cancer patients. The anti-PD-1/PD-L1 monotherapy had the lowest incidence of all types of AEs, while anti-PD-1/PD-L1+TKI and anti-PD-1/PD-L1+chemotherapy had the highest incidence of ≥grade 3 and any grade TRAEs, respectively. Therefore, although combinational therapy with standard medications might improve the outcomes, it accompanies by more AEs, which should be considered carefully in prostate cancer patients who are typically of older ages. A recent systematic review sought for evaluating the AEs following ICI therapy in patients with urologic cancers (95), demonstrating that prostate cancer patients had the highest rate of irAEs and ≥grade3 irAEs among all other urologic malignancies with a rate of 48.3% and 17.6%, respectively (95). The immunological reason why irAEs are more common in prostate cancer must be investigated in ongoing experimental studies. According to ICI monotherapy trials, we found that the most commonly reported any grade TRAEs included fatigue, diarrhea, and Infusion reactions. In this regard, Wang and colleagues performed a systematic review to assess the rate of different AEs related to ICI therapy for all human cancers (96); similarly, they found that the incidence of fatigue was the highest (18.3%) among all other AEs, which was lower relative to our estimates (24.9%). In addition, diarrhea and infusion reactions ranked third and fourteenth in this study, respectively with lower incidence rates relative to our findings (9.5% vs. 20.6% and 3.6% vs. 17.7%, respectively) (96).

## 5 Strength and limitations

Our systematic review was the first study intended to comprehensively investigate the efficacy and safety of ICIs for patients with prostate cancer. We included 35 trials, pooling the results of 3,618 participants into the data analysis. Applying novel approaches, we calculated the mOS and mPFS for the overall treatments as well as for categorized treatment regimens, separately. However, we faced a few limitations which should be considered when interpreting our findings. Firstly, we pooled the proportions obtained from single arms which might be potentially susceptible to bias as a result of variations in previous treatment lines, baseline differences in patient characteristics, target of medications, and dose and schedule of treatments. Secondly, inclusion of conference abstracts which were not peer-reviewed might be the another source of bias. Thirdly, we only included studies that examined the ICI combination therapies with conventional medications, while many trials investigated addition of ICIs to the state of the art immunotherapeutic agents. Therefore, future systematic reviews can also include novel combination approaches.

## 6 Conclusion and future perspective

Immunotherapy using monoclonal antibodies against immune checkpoint proteins assumed to be a promising opportunity for patients with prostate cancer; however, real-world data is not convincing even with ICI combination with conventional medications. We found that patients with advanced prostate cancer responded differently to ICI regimens and a variety of AEs were observed according to the type of administrated medications. While our findings may provide important guidance to clinicians in management of patients with prostate cancers, introduction of ICIs as the standard treatment of advanced prostate cancer requires further studies. Currently, trials are directed toward advanced combination approaches with novel immunotherapeutic medications. For instance, a bispecific T cell engagers (BITE) have been recently engineered to simultaneously bind to prostate-specific membrane antigen (PSMA) on tumor cells and CD3 on T cells, resulting in direct T cell activation and tumor cell lysis; the safety and efficacy of this BITE is being evaluated in combination with Pembrolizumab in the trial NCT03792841. Besides, conjugation of lutetium-177 to the PSMA ligand has been provided by Lu-PSMA-617 which is a radiopharmaceutical agent constructed to directly deliver radiation to prostate tumor cells; currently, addition of Lu-PSMA-617 to Pembrolizumab is being studied in the NCT03658447 trial. Furthermore, the other treatment modalities such as chimeric antigen receptor T cell (CAR-T) therapy and cancer vaccines have been developed for prostate cancer patients which are currently at early phases of clinical trials and can be given as combination therapy with ICIs in near future (97–99). All in all, while ICIs ^:^either as single agents or in a combined-modal strategy with conventional medications^:^may partially improve survival of patients with advanced prostate cancer, advanced combination approaches with novel immunotherapeutic medications may pave the way for successful therapeutic strategies using immunotherapy in this malignancy.

## Author contributions

MN designed the study. MN, SA, and FA performed the study selection and data extraction. MN conducted the analysis. MN, SA, FA, FF, AM, and DB wrote the first draft of the manuscript. DB, MK, and AK critically revised the manuscript. All authors reviewed the drafted manuscript for critical content. All authors contributed to the article and approved the submitted version.
