# Supplementary material for: Efficacy and safety of immune checkpoint inhibitors for patients with prostate cancer: a systematic review and meta-analysis
Source: Front Immunol. 2023 Oct 31;14:1181051. doi: 10.3389/fimmu.2023.1181051 (PMC10644317; doi:10.3389/fimmu.2023.1181051)
Supplement: Supplementary file 1 [file DataSheet_1.docx]

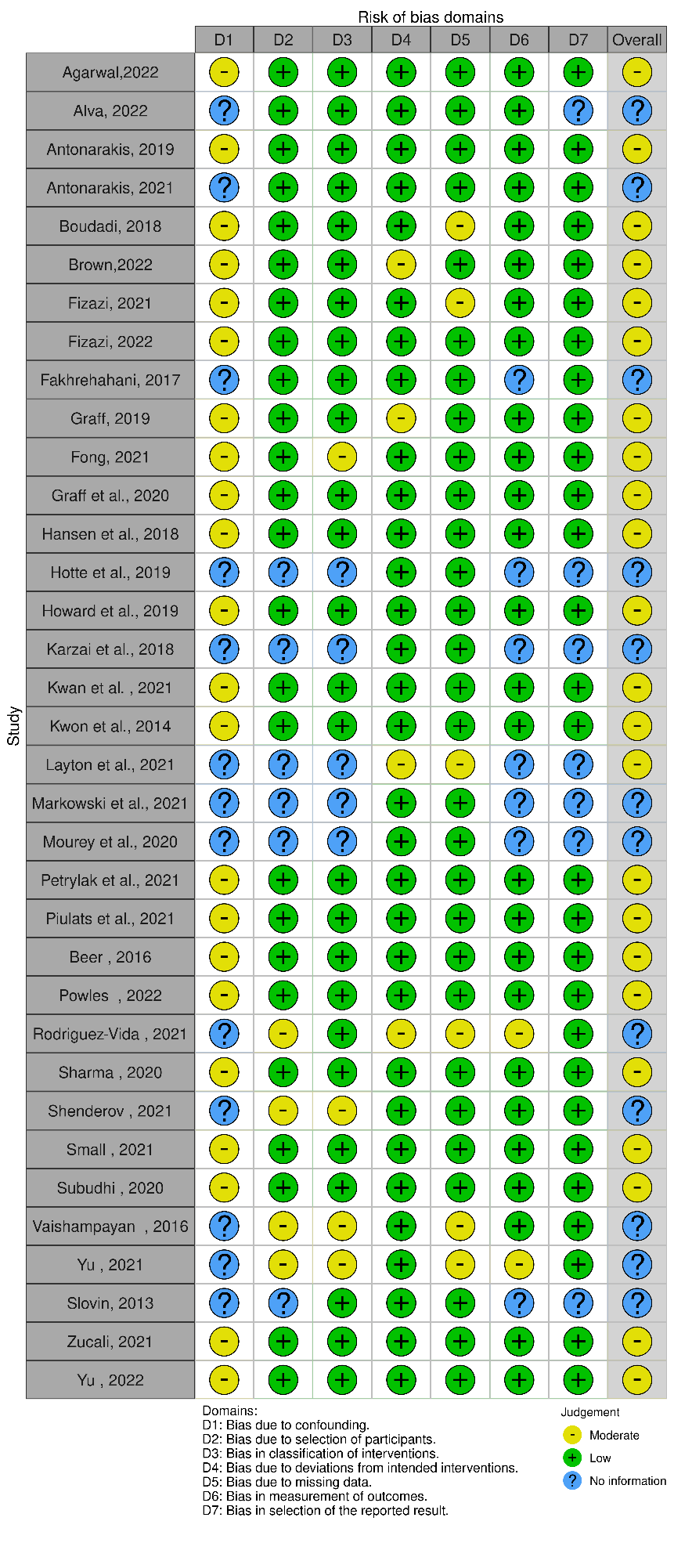


**Figure S1.** Quality of included trials based on the tool for assessing risk of bias in non-randomized studies of interventions (ROBINS-I)


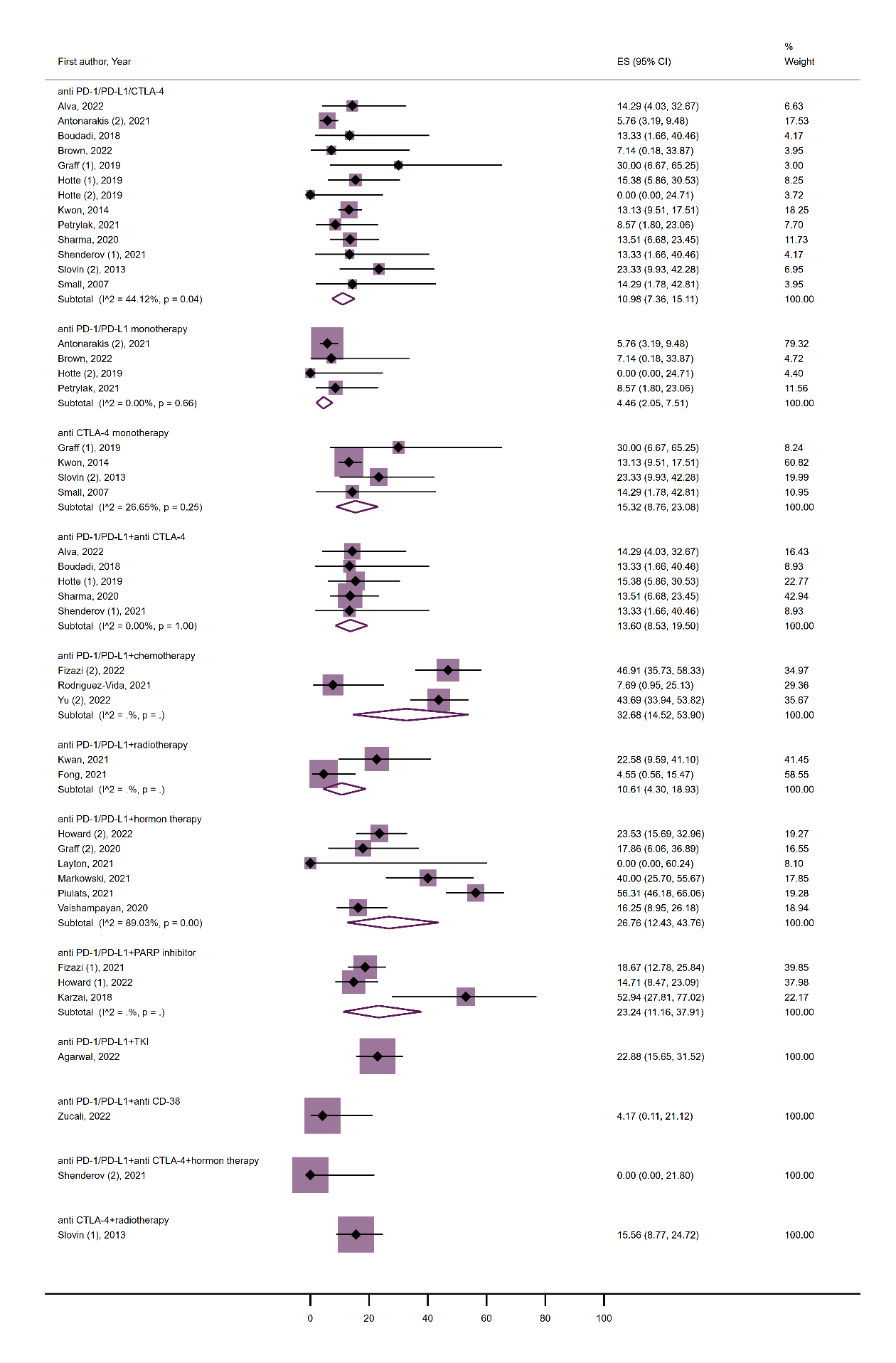


**Figure S2.** Pooled results of prostate specific antigen response rates by immune checkpoint inhibitor medication subgroups.


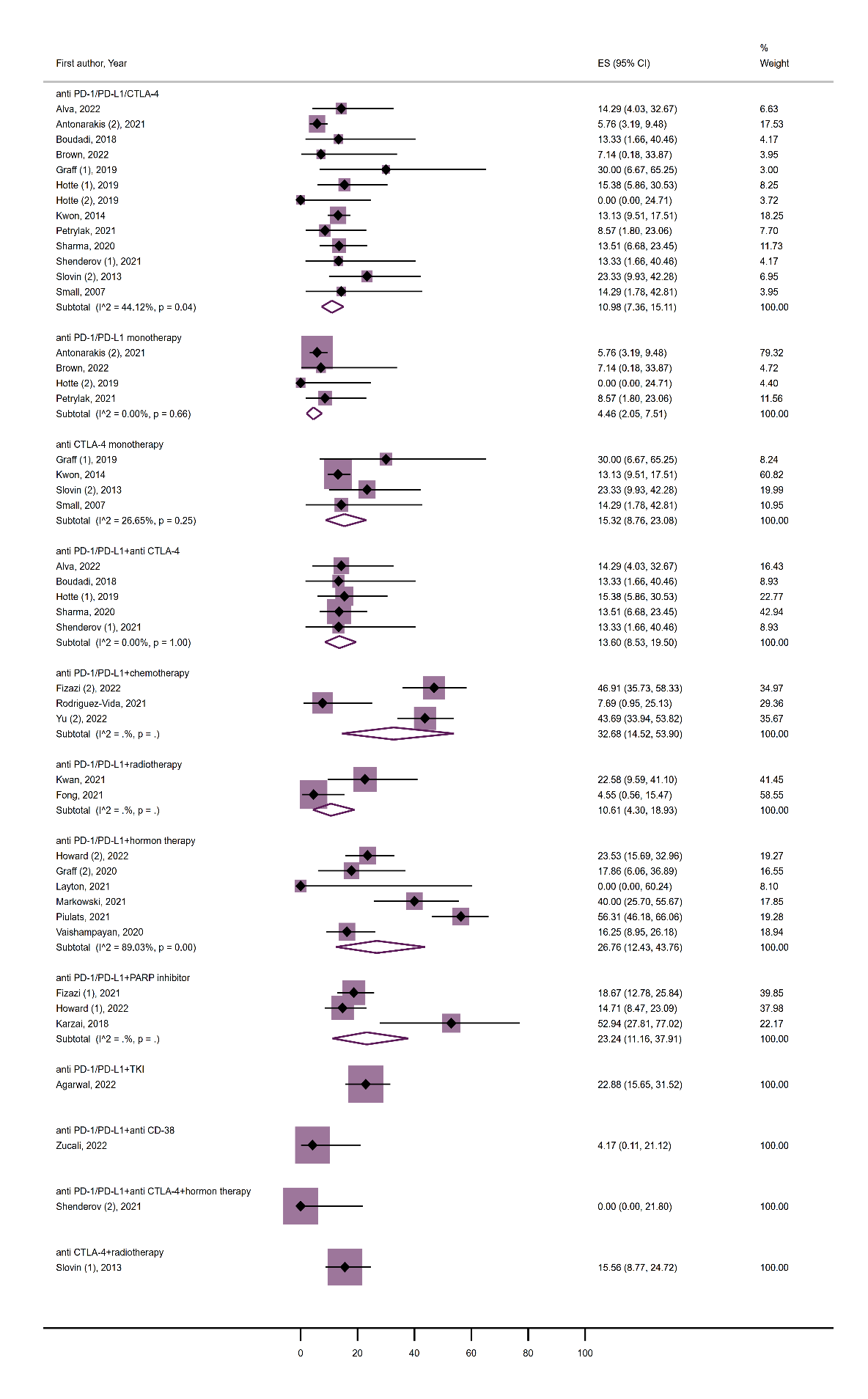


**Figure S3.** Pooled results of objective response rates by immune checkpoint inhibitor medication subgroups.


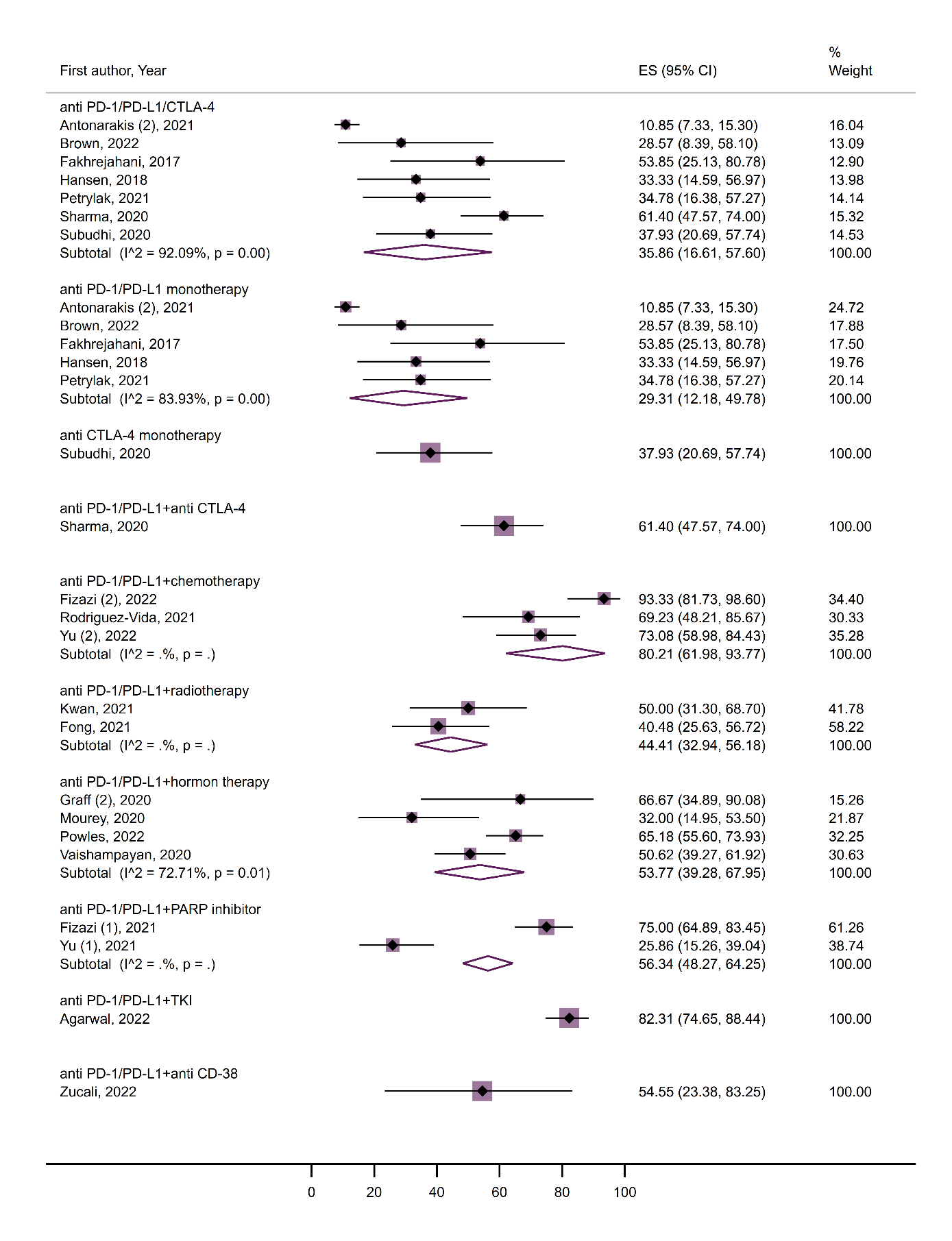


**Figure S4.** Pooled results of disease control rates by immune checkpoint inhibitor medication subgroups.


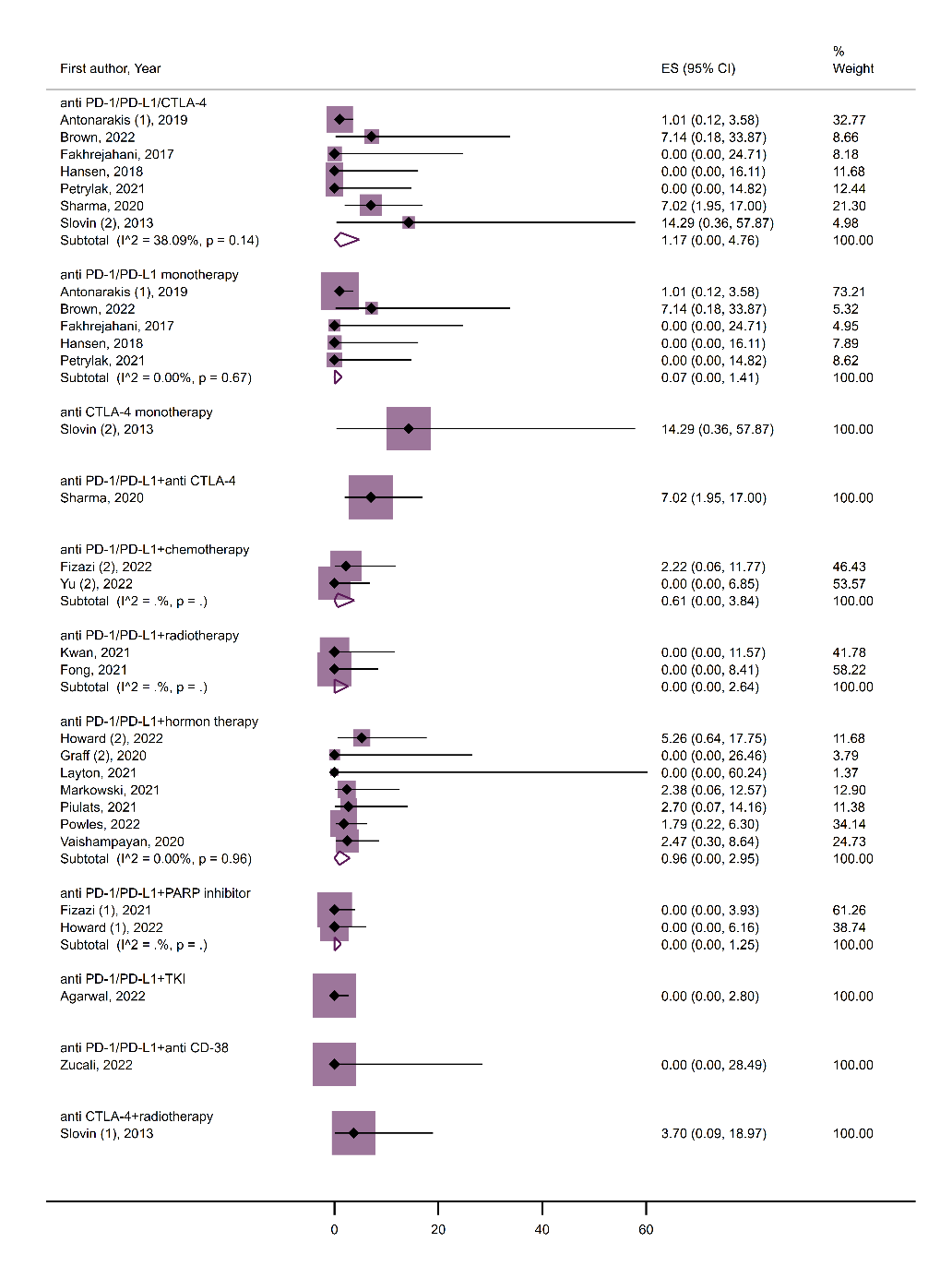


**Figure S5.** Pooled results of complete response rates by immune checkpoint inhibitor medication subgroups.


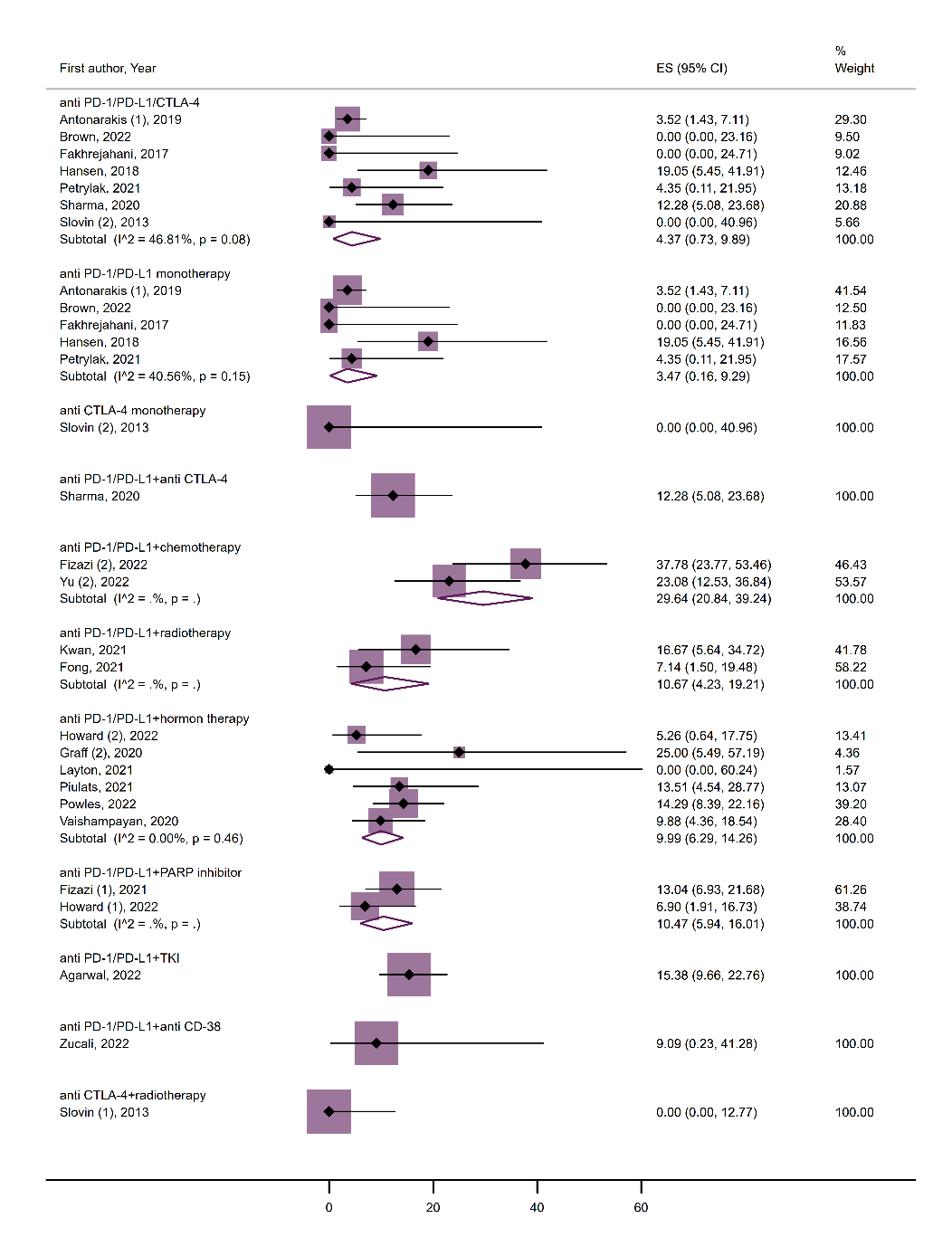


**Figure S6.** Pooled results of partial response rates by immune checkpoint inhibitor medication subgroups.


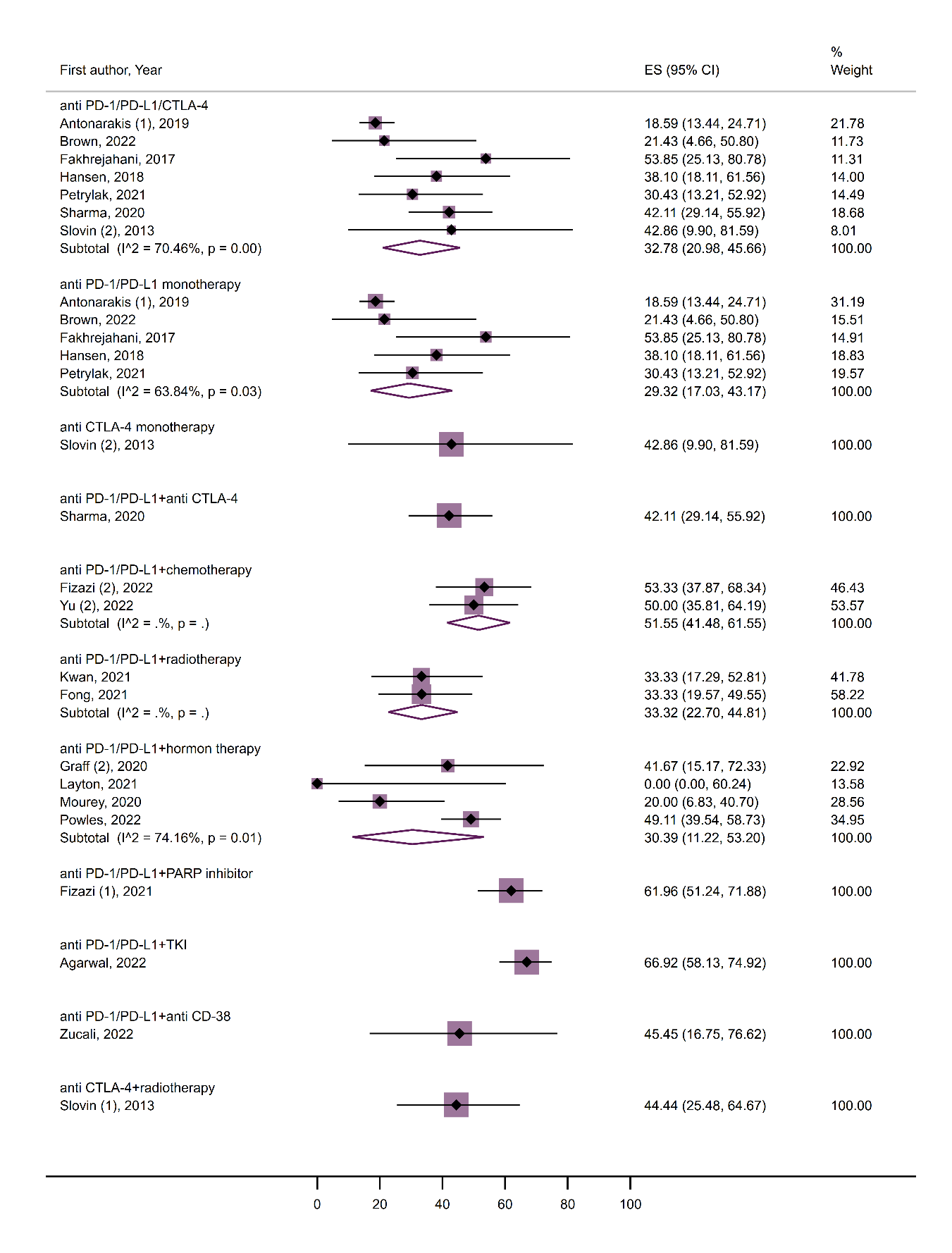


**Figure S7**. Pooled results of stable disease rates by immune checkpoint inhibitor medication subgroups.


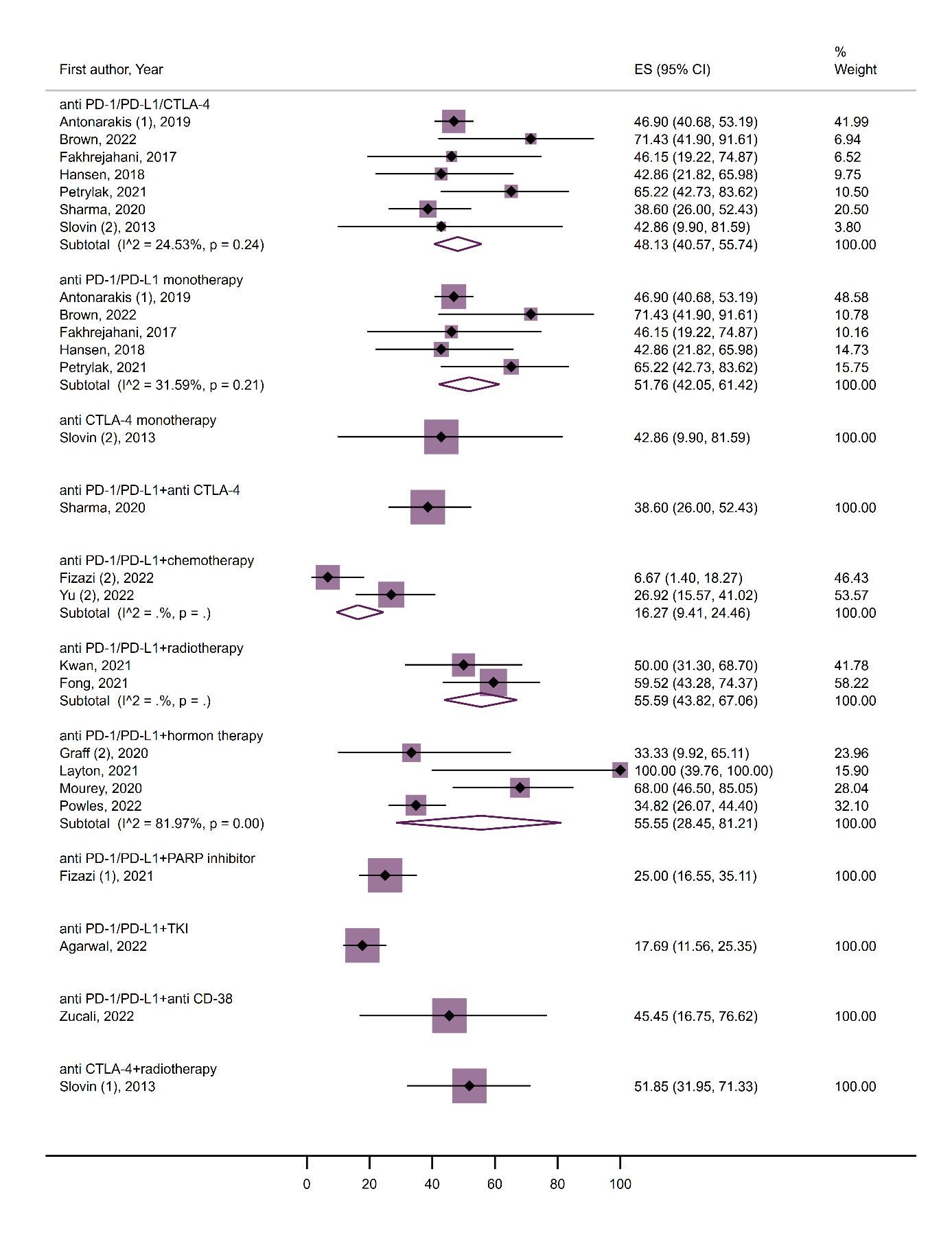


**Figure S8.** Pooled results of progressive disease rates by immune checkpoint inhibitor medication subgroups.


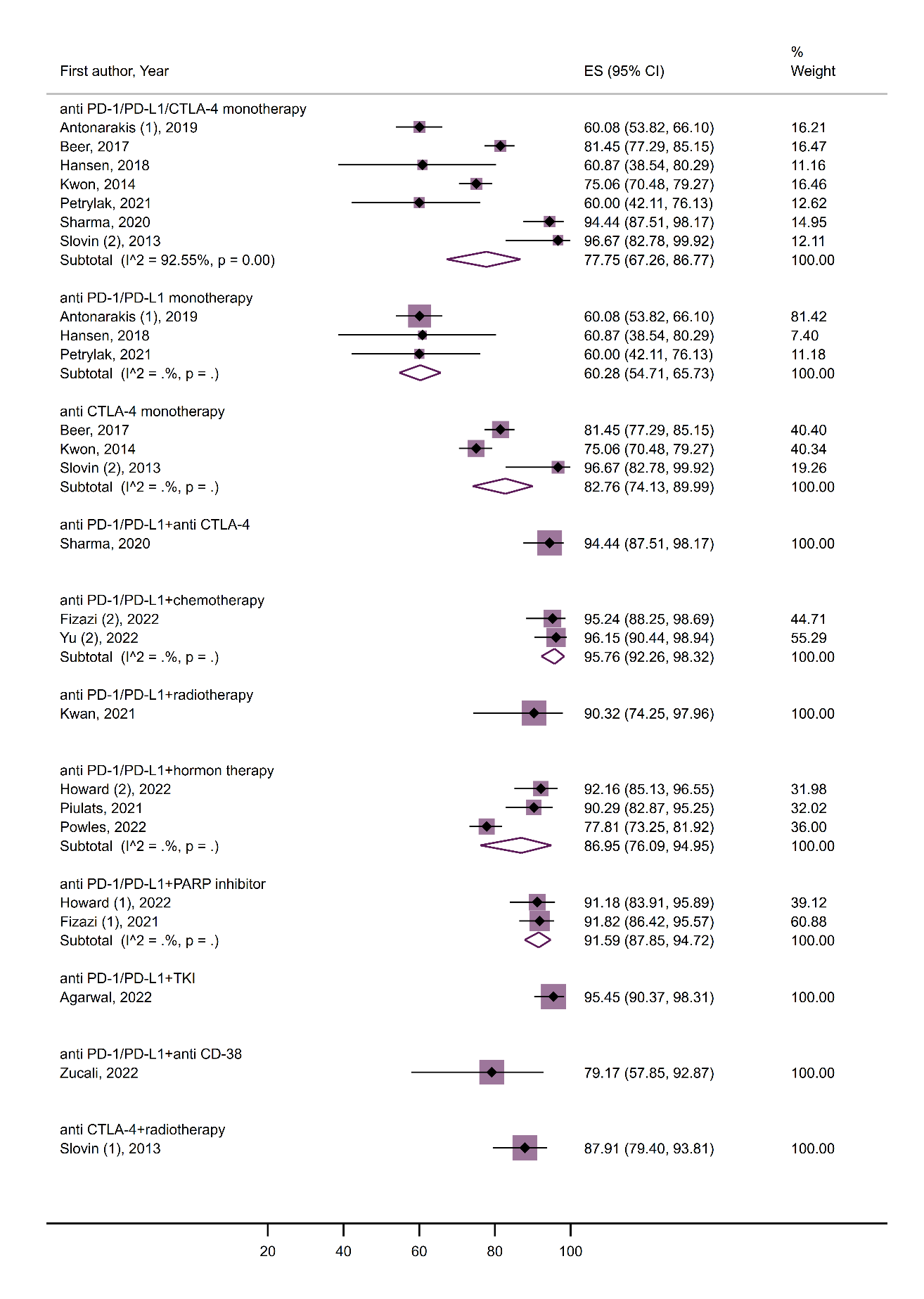


**Figure S9.** Pooled results of any grade treatment-related adverse events by immune checkpoint inhibitor medication subgroups.


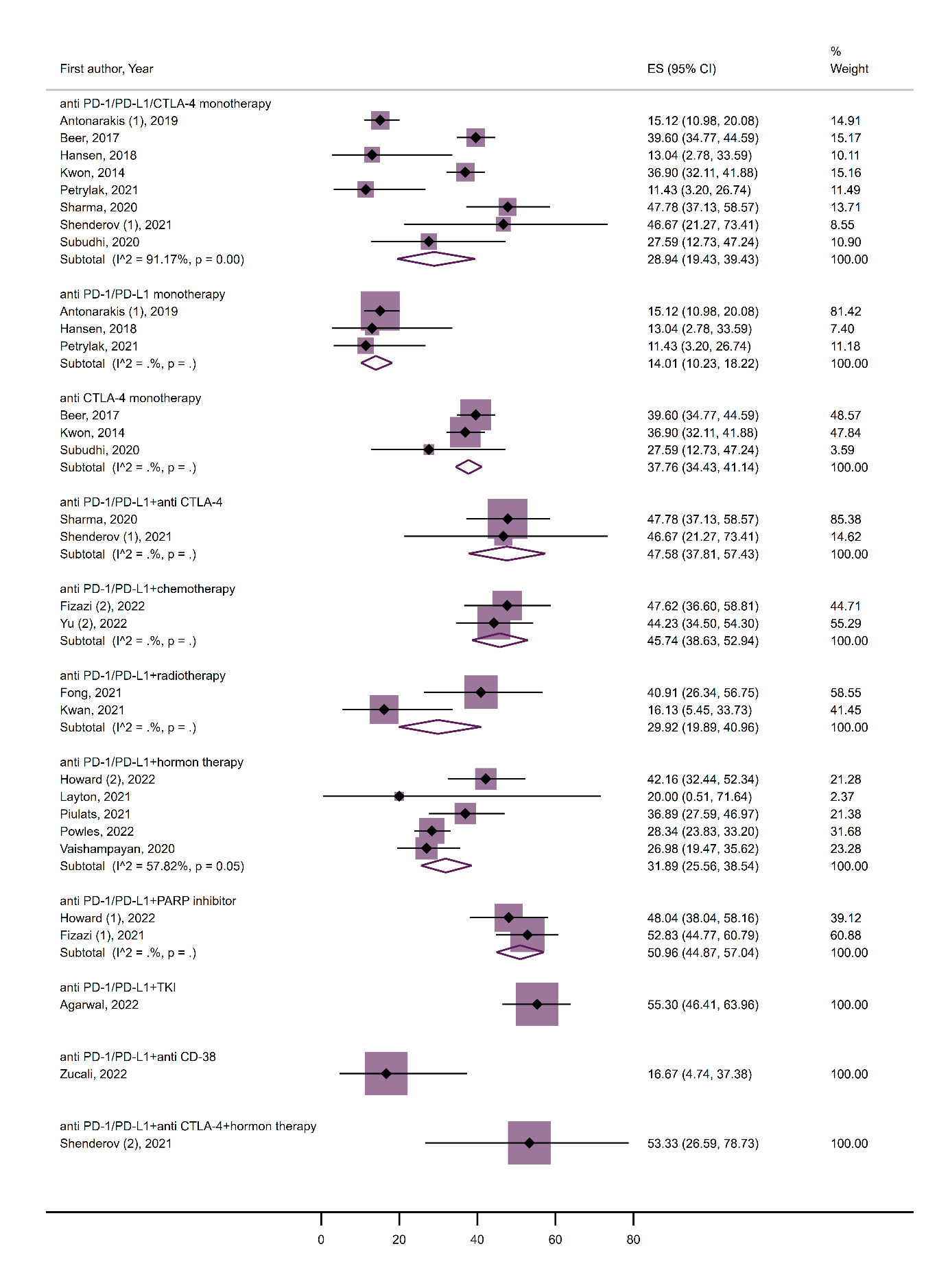


**Figure S10.** Pooled results of ≥ grade 3 treatment-related adverse events by immune checkpoint inhibitor medication subgroups.


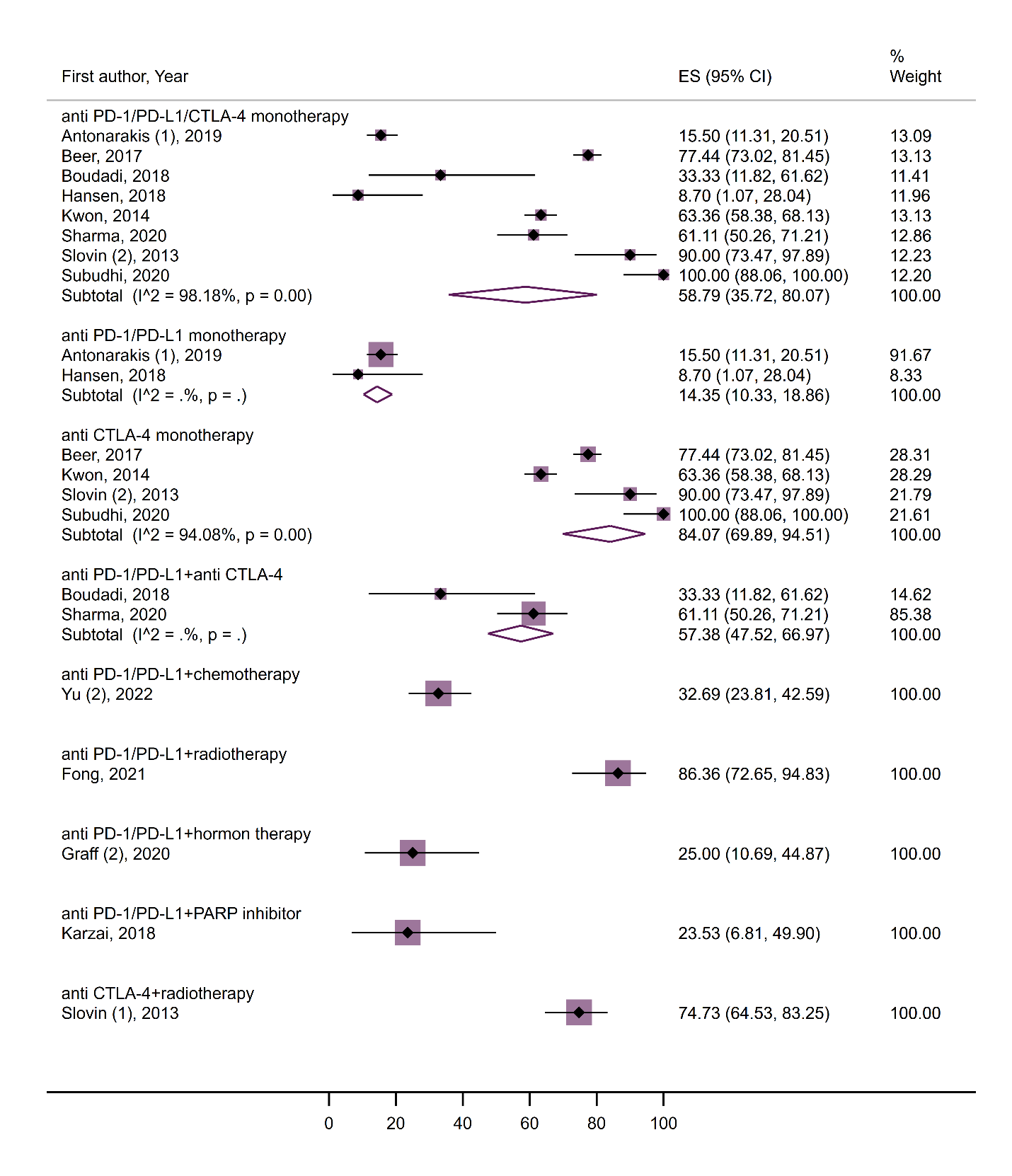


**Figure S11.** Pooled results of immune-related adverse events by immune checkpoint inhibitor medication subgroups.

**Figure S12.** Pooled results of serious adverse events by immune checkpoint inhibitor medication subgroups.


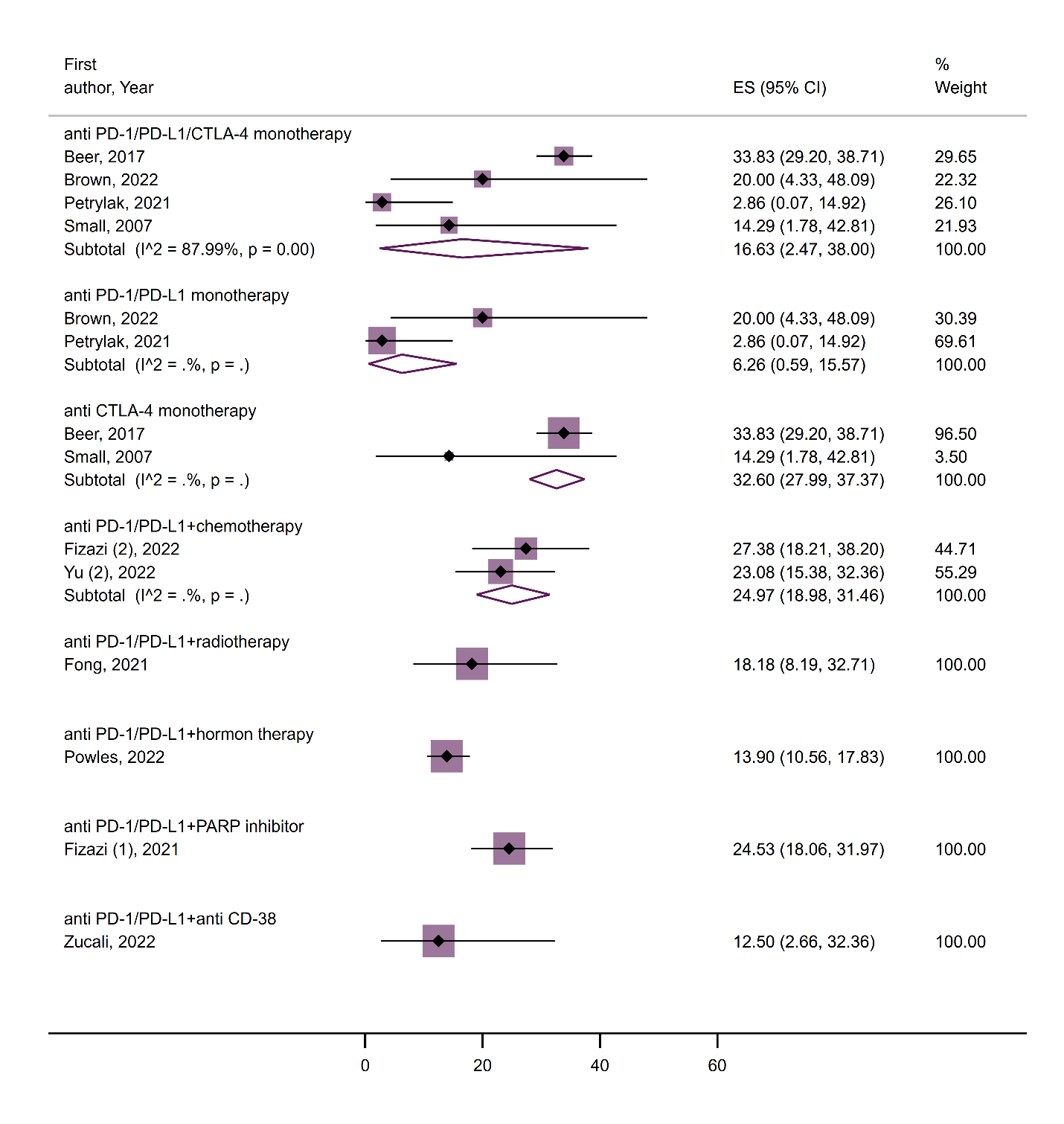

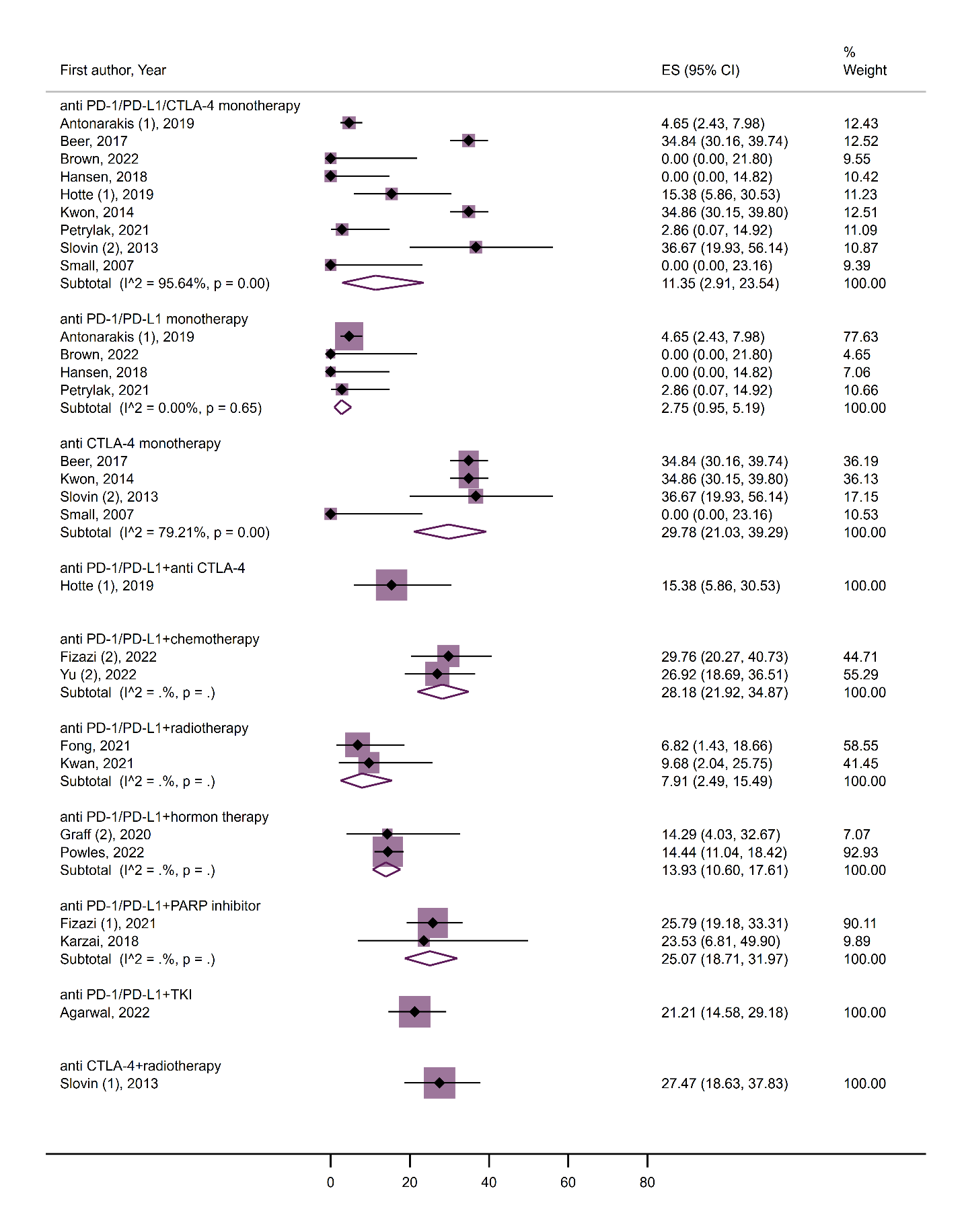


**Figure S13.** Pooled results of adverse events led to treatment discontinuation by immune checkpoint inhibitor medication subgroups.


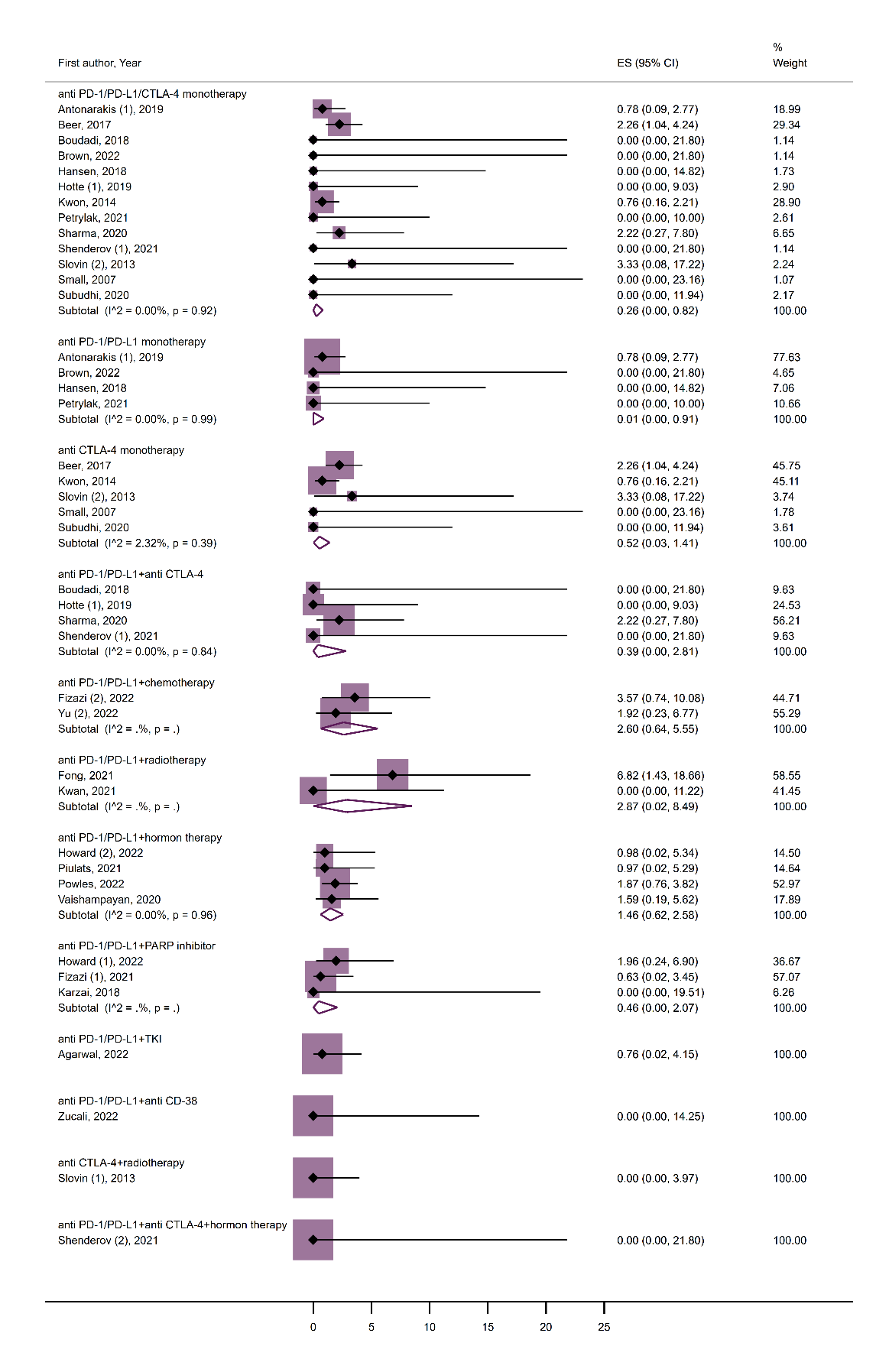

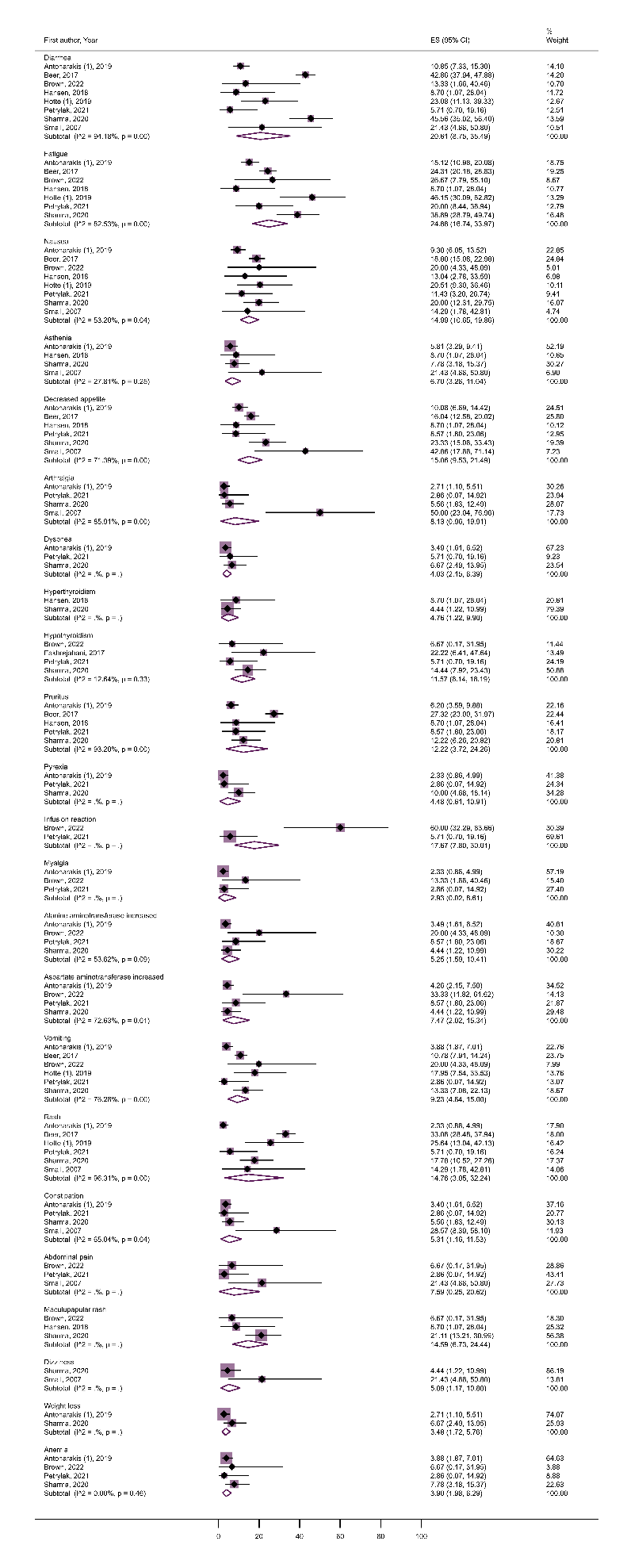

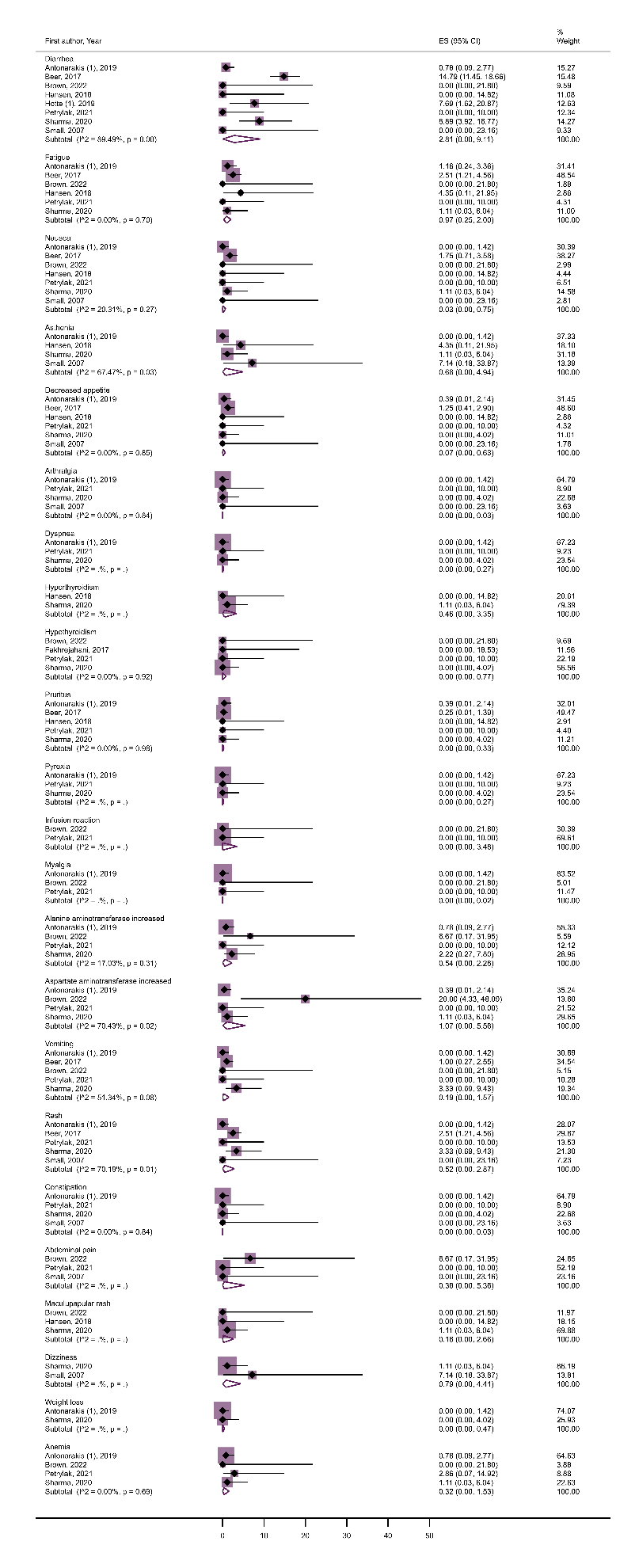


**Figure S14**. Pooled results of adverse events led to death by immune checkpoint inhibitor medication subgroups.

**Figure S15.** Pooled results of any grade treatment-related adverse events caused by immune checkpoint inhibitor monotherapy treatment regimen groups.

**Figure S16.** Pooled results of all ≥ grade3 treatment-related adverse events caused by immune checkpoint inhibitor monotherapy treatment regimen groups.

**Table S1.** Search strategy.

| Database  (Search date) | Step | Search strategy | Number of results |
| --- | --- | --- | --- |
| PubMed  (8.19.2022) | #1 | “Prostatic Neoplasms”[MeSH] OR Prostatic[tiab] OR prostate[tiab] OR “prostate neoplasm*”[tiab] OR “prostatic neoplasm*”[tiab] OR “prostate cancer*”[tiab] OR “prostatic cancer*”[tiab] OR “cancer of prostate”[tiab] OR “neoplasm of prostate”[tiab] OR “cancer of the prostate”[tiab] OR “neoplasm of the prostate”[tiab] OR “adenocarcinoma of prostate”[tiab] OR “adenocarcinoma of the prostate”[tiab] OR “squamous cell carcinoma of prostate”[tiab] OR “squamous cell carcinoma of the prostate”[tiab] OR “transitional cell carcinoma of prostate”[tiab] OR “transitional cell carcinoma of the prostate”[tiab] OR “cancers of prostate”[tiab] OR “neoplasms of prostate”[tiab] OR “cancers of the prostate”[tiab] OR “neoplasms of the prostate”[tiab] OR “adenocarcinomas of prostate”[tiab] OR “adenocarcinomas of the prostate”[tiab] OR “squamous cell carcinomas of prostate”[tiab] OR “squamous cell carcinomas of the prostate”[tiab] OR “transitional cell carcinomas of prostate”[tiab] OR “transitional cell carcinomas of the prostate”[tiab] OR “Prostatic Neoplasms, Castration-Resistant”[MeSH] OR “Castration-Resistant Prostate Neoplasm*”[tiab] OR “Castration Resistant Prostate Neoplasm*”[tiab] OR “Castration-Resistant Prostate Cancer*”[tiab] OR “Castration Resistant Prostate cancer*”[tiab] OR “Androgen-Resistant Prostate Neoplasm*”[tiab] OR “Androgen Resistant Prostate Neoplasm*”[tiab] OR “Androgen-Resistant Prostate Cancer*”[tiab] OR “Androgen Resistant Prostate cancer*”[tiab] OR “Androgen-Insensitive Prostate Neoplasm*”[tiab] OR “Androgen Insensitive Prostate Neoplasm*”[tiab] OR “Androgen-Insensitive Prostate Cancer*”[tiab] OR “Androgen Insensitive Prostate cancer*”[tiab] OR “Androgen-Independent Prostate Neoplasm*”[tiab] OR “Androgen Independent Prostate Neoplasm*”[tiab] OR “Androgen-Independent Prostate Cancer*”[tiab] OR “Androgen Independent Prostate cancer*”[tiab] OR “Hormone refractory Prostate Neoplasm*”[tiab] OR “Hormone refractory Prostate Neoplasm*”[tiab] OR “Hormone refractory Prostate Cancer*”[tiab] OR “Hormone refractory Prostate cancer*”[tiab] OR “Castration-Resistant Prostatic Neoplasm*”[tiab] OR “Castration Resistant Prostatic Neoplasm*”[tiab] OR “Castration-Resistant Prostatic Cancer*”[tiab] OR “Castration Resistant Prostatic cancer*”[tiab] OR “Androgen-Resistant Prostatic Neoplasm*”[tiab] OR “Androgen Resistant Prostatic Neoplasm*”[tiab] OR “Androgen-Resistant Prostatic Cancer*”[tiab] OR “Androgen Resistant Prostatic cancer*”[tiab] OR “Androgen-Insensitive Prostatic Neoplasm*”[tiab] OR “Androgen Insensitive Prostatic Neoplasm*”[tiab] OR “Androgen-Insensitive Prostatic Cancer*”[tiab] OR “Androgen Insensitive Prostatic cancer*”[tiab] OR “Androgen-Independent Prostatic Neoplasm*”[tiab] OR “Androgen Independent Prostatic Neoplasm*”[tiab] OR “Androgen-Independent Prostatic Cancer*”[tiab] OR “Androgen Independent Prostatic cancer*”[tiab] OR “Hormone refractory Prostatic Neoplasm*”[tiab] OR “Hormone refractory Prostatic Neoplasm*”[tiab] OR “Hormone refractory Prostatic Cancer*”[tiab] OR “Hormone refractory Prostatic cancer*”[tiab] | 244,576 |
|  | #2 | “Immune Checkpoint Inhibitors”[MeSH] OR “Immune Checkpoint Inhibitor*”[tiab] OR “Immune Checkpoint Blocker*”[tiab] OR “Immune Checkpoint Blockade*”[tiab] OR “Immune Checkpoint Inhibition*”[tiab] OR “PD-L1 Inhibitor*”[tiab] OR “PD L1 Inhibitor*”[tiab] OR “PDL1 Inhibitor*”[tiab] OR “PD-L1 blocker*”[tiab] OR “PD L1 blocker*”[tiab] OR “PDL1 blocker*”[tiab] OR “Programmed Death-Ligand 1 Inhibitor*”[tiab] OR “Programmed Death Ligand 1 Inhibitor*”[tiab] OR “Programmed Death-Ligand 1 blocker*”[tiab] OR “Programmed Death Ligand 1 blocker*”[tiab] OR “anti Programmed Death-Ligand 1”[tiab] OR “anti-Programmed Death-Ligand 1”[tiab] OR “anti Programmed Death Ligand 1”[tiab] OR “anti-Programmed Death Ligand 1”[tiab] OR “CTLA-4 Inhibitor*”[tiab] OR “CTLA 4 Inhibitor*”[tiab] OR “CTLA4 Inhibitor*”[tiab] OR “CTLA-4 blocker*”[tiab] OR “CTLA 4 blocker*”[tiab] OR “CTLA4 blocker*”[tiab] OR “Cytotoxic T-Lymphocyte Associated Protein 4 Inhibitor*”[tiab] OR “Cytotoxic T Lymphocyte-Associated Protein 4 Inhibitor*”[tiab] OR “Cytotoxic T-Lymphocyte Associated Protein 4 blocker*”[tiab] OR “Cytotoxic T Lymphocyte-Associated Protein 4 blocker*”[tiab] OR “Cytotoxic T-Lymphocyte Associated antigen 4 Inhibitor*”[tiab] OR “Cytotoxic T Lymphocyte-Associated antigen 4 Inhibitor*”[tiab] OR “Cytotoxic T-Lymphocyte Associated antigen 4 blocker*”[tiab] OR “Cytotoxic T Lymphocyte-Associated antigen 4 blocker*”[tiab] OR “Cytotoxic T-Lymphocyte-Associated Protein 4 Inhibitor*”[tiab] OR “Cytotoxic T Lymphocyte Associated Protein 4 Inhibitor*”[tiab] OR “Cytotoxic T-Lymphocyte-Associated Protein 4 blocker*”[tiab] OR “Cytotoxic T Lymphocyte Associated Protein 4 blocker*”[tiab] OR “Cytotoxic T-Lymphocyte-Associated antigen 4 Inhibitor*”[tiab] OR “Cytotoxic T Lymphocyte Associated antigen 4 Inhibitor*”[tiab] OR “Cytotoxic T-Lymphocyte-Associated antigen 4 blocker*”[tiab] OR “Cytotoxic T Lymphocyte Associated antigen 4 blocker*”[tiab] OR “anti-cytotoxic T lymphocyte-associated antigen 4”[tiab] OR “anti cytotoxic T lymphocyte-associated antigen 4”[tiab] OR “anti-cytotoxic T lymphocyte-associated protein 4”[tiab] OR “anti cytotoxic T lymphocyte-associated protein 4”[tiab] OR “anti-cytotoxic T-lymphocyte-associated antigen 4”[tiab] OR “anti-cytotoxic T-lymphocyte-associated antigen 4”[tiab] OR “PD-1 Inhibitor*”[tiab] OR “PD 1 Inhibitor*”[tiab] OR “PD1 Inhibitor*”[tiab] OR “PD 1 blocker*”[tiab] OR “PD1 blocker*”[tiab] OR “PD-1 blocker*”[tiab] OR “Programmed Cell Death Protein 1 Inhibitor*”[tiab] OR “Programmed Cell Death Protein 1 blocker*”[tiab] OR “anti Programmed Cell Death Protein 1”[tiab] OR “anti-Programmed Cell Death Protein 1”[tiab] OR “anti-PD1”[tiab] OR “anti PD1”[tiab] OR “anti-PD-1”[tiab] OR “anti PD-1”[tiab] OR “anti-PD-L1”[tiab] OR “anti PDL1”[tiab] OR “anti PD-L1”[tiab] OR “anti-PDL1”[tiab] OR “anti-PD L1”[tiab] OR “anti PD L1”[tiab] OR “anti-CTLA4”[tiab] OR “anti-CTLA 4”[tiab] OR “anti-CTLA-4”[tiab] OR “anti CTLA4”[tiab] OR “anti CTLA 4”[tiab] OR “anti CTLA-4”[tiab] OR “PD-1-PD-L1 Blockade*”[tiab] OR “PD 1 PD L1 Blockade*”[tiab] OR “Pembrolizumab”[tiab] OR “MK-3475”[tiab] OR “lambrolizumab”[tiab] OR “Keytruda”[tiab] OR “SCH-900475”[tiab] OR “Nivolumab”[tiab] OR “Opdivo”[tiab] OR “ONO-4538”[tiab] OR “ONO 4538”[tiab] OR “ONO4538”[tiab] OR “MDX-1106”[tiab] OR “MDX 1106”[tiab] OR “MDX1106”[tiab] OR “BMS-936558”[tiab] OR “BMS 936558”[tiab] OR “BMS936558”[tiab] OR “Ipilimumab”[tiab] OR “Yervoy”[tiab] OR “MDX 010”[tiab] OR “MDX010”[tiab] OR “MDX-010”[tiab] OR “MDX-CTLA-4”[tiab] OR “MDX CTLA 4”[tiab] OR “Durvalumab”[tiab] OR “MEDI4736”[tiab] OR “MEDI-4736”[tiab] OR “Imfinzi”[tiab] OR “Dostarlimab”[tiab] OR “GSK4057190”[tiab] OR “TSR-042”[tiab] OR “Cemiplimab”[tiab] OR ”REGN2810”[tiab] OR “Avelumab”[tiab] OR “MSB-0010682”[tiab] OR “MSB0010682”[tiab] OR ”bavencio”[tiab] OR ”MSB0010718C”[tiab] OR ”MSB-0010718C”[tiab] OR “Atezolizumab”[tiab] OR “MPDL3280A”[tiab] OR ”MPDL-3280A”[tiab] OR “Tecentriq”[tiab] OR “RG7446”[tiab] OR “RG-7446”[tiab] OR “pidilizumab”[tiab] OR “CT-011”[tiab] OR “CT 011”[tiab] OR “tremelimumab”[tiab] OR “ticilimumab”[tiab] OR “CP 675”[tiab] OR “CP675 cpd”[tiab] OR “CP-675”[tiab] OR “CP-675,206”[tiab] OR “CP-675206”[tiab] OR “CP675206”[tiab] OR “CP 675206”[tiab] OR "dostarlimab"[Supplementary Concept] OR "cemiplimab"[Supplementary Concept] OR "Ipilimumab"[Mesh] OR "tremelimumab"[Supplementary Concept] OR "pembrolizumab"[Supplementary Concept] OR "atezolizumab"[Supplementary Concept] OR "Nivolumab"[Mesh] OR "durvalumab"[Supplementary Concept] OR "avelumab" [Supplementary Concept] OR "pidilizumab" [Supplementary Concept] OR “sintilimab”[tiab] OR “SHR-1210”[taib] OR “camrelizumab”[tiab] OR “toripalimab”[tiab] OR “HX008”[tiab] OR “tremelimumab”[tiab] | 41,660 |
|  | #3 | “RCT”[tiab] OR “trial*”[tiab] OR “intervention*”[tiab] OR “placebo”[tiab] OR “randomised trial*”[tiab] OR “randomized trial*”[tiab] OR “controlled trial*”[tiab] OR "Random Allocation"[Mesh] OR "Single-Blind Method"[Mesh] OR "Double-Blind Method"[Mesh] OR "Cross-Over Studies"[Mesh] OR "Clinical Trial"[Publication Type] OR "Clinical Trials as Topic"[Mesh] OR "Controlled Clinical Trial"[Publication Type] OR "Clinical Trial, Phase I"[Publication Type] OR "Clinical Trial, Phase II"[Publication Type] OR "Clinical Trial, Phase III"[Publication Type] OR "Clinical Trial, Phase IV"[Publication Type] OR "Non-Randomized Controlled Trials as Topic"[Mesh] | 2,993,526 |
|  | #4 | #1 AND #2 AND #3 | 409 |
| Scopus  (8.19.2022) | #1 | TITLE-ABS-KEY(Prostatic OR prostate OR “prostate neoplasm*” OR “prostatic neoplasm*” OR “prostate cancer*” OR “prostatic cancer*” OR “cancer of prostate” OR “neoplasm of prostate” OR “cancer of the prostate” OR “neoplasm of the prostate” OR “adenocarcinoma of prostate” OR “adenocarcinoma of the prostate” OR “squamous cell carcinoma of prostate” OR “squamous cell carcinoma of the prostate” OR “transitional cell carcinoma of prostate” OR “transitional cell carcinoma of the prostate” OR “cancers of prostate” OR “neoplasms of prostate” OR “cancers of the prostate” OR “neoplasms of the prostate” OR “adenocarcinomas of prostate” OR “adenocarcinomas of the prostate” OR “squamous cell carcinomas of prostate” OR “squamous cell carcinomas of the prostate” OR “transitional cell carcinomas of prostate” OR “transitional cell carcinomas of the prostate” OR “Castration-Resistant Prostate Neoplasm*” OR “Castration Resistant Prostate Neoplasm*” OR “Castration-Resistant Prostate Cancer*” OR “Castration Resistant Prostate cancer*” OR “Androgen-Resistant Prostate Neoplasm*” OR “Androgen Resistant Prostate Neoplasm*” OR “Androgen-Resistant Prostate Cancer*” OR “Androgen Resistant Prostate cancer*” OR “Androgen-Insensitive Prostate Neoplasm*” OR “Androgen Insensitive Prostate Neoplasm*” OR “Androgen-Insensitive Prostate Cancer*” OR “Androgen Insensitive Prostate cancer*” OR “Androgen-Independent Prostate Neoplasm*” OR “Androgen Independent Prostate Neoplasm*” OR “Androgen-Independent Prostate Cancer*” OR “Androgen Independent Prostate cancer*” OR “Hormone refractory Prostate Neoplasm*” OR “Hormone refractory Prostate Neoplasm*” OR “Hormone refractory Prostate Cancer*” OR “Hormone refractory Prostate cancer*” OR “Castration-Resistant Prostatic Neoplasm*” OR “Castration Resistant Prostatic Neoplasm*” OR “Castration-Resistant Prostatic Cancer*” OR “Castration Resistant Prostatic cancer*” OR “Androgen-Resistant Prostatic Neoplasm*” OR “Androgen Resistant Prostatic Neoplasm*” OR “Androgen-Resistant Prostatic Cancer*” OR “Androgen Resistant Prostatic cancer*” OR “Androgen-Insensitive Prostatic Neoplasm*” OR “Androgen Insensitive Prostatic Neoplasm*” OR “Androgen-Insensitive Prostatic Cancer*” OR “Androgen Insensitive Prostatic cancer*” OR “Androgen-Independent Prostatic Neoplasm*” OR “Androgen Independent Prostatic Neoplasm*” OR “Androgen-Independent Prostatic Cancer*” OR “Androgen Independent Prostatic cancer*” OR “Hormone refractory Prostatic Neoplasm*” OR “Hormone refractory Prostatic Neoplasm*” OR “Hormone refractory Prostatic Cancer*” OR “Hormone refractory Prostatic cancer*”) | 340,210 |
|  | #2 | TITLE-ABS-KEY(“Immune Checkpoint Inhibitor*” OR “Immune Checkpoint Blocker*” OR “Immune Checkpoint Blockade*” OR “Immune Checkpoint Inhibition*” OR “PD-L1 Inhibitor*” OR “PD L1 Inhibitor*” OR “PDL1 Inhibitor*” OR “PD-L1 blocker*” OR “PD L1 blocker*” OR “PDL1 blocker*” OR “Programmed Death-Ligand 1 Inhibitor*” OR “Programmed Death Ligand 1 Inhibitor*” OR “Programmed Death-Ligand 1 blocker*” OR “Programmed Death Ligand 1 blocker*” OR “anti Programmed Death-Ligand 1” OR “anti-Programmed Death-Ligand 1” OR “anti Programmed Death Ligand 1” OR “anti-Programmed Death Ligand 1” OR “CTLA-4 Inhibitor*” OR “CTLA 4 Inhibitor*” OR “CTLA4 Inhibitor*” OR “CTLA-4 blocker*” OR “CTLA 4 blocker*” OR “CTLA4 blocker*” OR “Cytotoxic T-Lymphocyte Associated Protein 4 Inhibitor*” OR “Cytotoxic T Lymphocyte-Associated Protein 4 Inhibitor*” OR “Cytotoxic T-Lymphocyte Associated Protein 4 blocker*” OR “Cytotoxic T Lymphocyte-Associated Protein 4 blocker*” OR “Cytotoxic T-Lymphocyte Associated antigen 4 Inhibitor*” OR “Cytotoxic T Lymphocyte-Associated antigen 4 Inhibitor*” OR “Cytotoxic T-Lymphocyte Associated antigen 4 blocker*” OR “Cytotoxic T Lymphocyte-Associated antigen 4 blocker*” OR “Cytotoxic T-Lymphocyte-Associated Protein 4 Inhibitor*” OR “Cytotoxic T Lymphocyte Associated Protein 4 Inhibitor*” OR “Cytotoxic T-Lymphocyte-Associated Protein 4 blocker*” OR “Cytotoxic T Lymphocyte Associated Protein 4 blocker*” OR “Cytotoxic T-Lymphocyte-Associated antigen 4 Inhibitor*” OR “Cytotoxic T Lymphocyte Associated antigen 4 Inhibitor*” OR “Cytotoxic T-Lymphocyte-Associated antigen 4 blocker*” OR “Cytotoxic T Lymphocyte Associated antigen 4 blocker*” OR “anti-cytotoxic T lymphocyte-associated antigen 4” OR “anti cytotoxic T lymphocyte-associated antigen 4” OR “anti-cytotoxic T lymphocyte-associated protein 4” OR “anti cytotoxic T lymphocyte-associated protein 4” OR “anti-cytotoxic T-lymphocyte-associated antigen 4” OR “anti-cytotoxic T-lymphocyte-associated antigen 4” OR “PD-1 Inhibitor*” OR “PD 1 Inhibitor*” OR “PD1 Inhibitor*” OR “PD 1 blocker*” OR “PD1 blocker*” OR “PD-1 blocker*” OR “Programmed Cell Death Protein 1 Inhibitor*” OR “Programmed Cell Death Protein 1 blocker*” OR “anti Programmed Cell Death Protein 1” OR “anti-Programmed Cell Death Protein 1” OR “anti-PD1” OR “anti PD1” OR “anti-PD-1” OR “anti PD-1” OR “anti-PD-L1” OR “anti PDL1” OR “anti PD-L1” OR “anti-PDL1” OR “anti-PD L1” OR “anti PD L1” OR “anti-CTLA4” OR “anti-CTLA 4” OR “anti-CTLA-4” OR “anti CTLA4” OR “anti CTLA 4” OR “anti CTLA-4” OR “PD-1-PD-L1 Blockade*” OR “PD 1 PD L1 Blockade*” OR “Pembrolizumab” OR “MK-3475” OR “lambrolizumab” OR “Keytruda” OR “SCH-900475” OR “Nivolumab” OR “Opdivo” OR “ONO-4538” OR “ONO 4538” OR “ONO4538” OR “MDX-1106” OR “MDX 1106” OR “MDX1106” OR “BMS-936558” OR “BMS 936558” OR “BMS936558” OR “Ipilimumab” OR “Yervoy” OR “MDX 010” OR “MDX010” OR “MDX-010” OR “MDX-CTLA-4” OR “MDX CTLA 4” OR “Durvalumab” OR “MEDI4736” OR “MEDI-4736” OR “Imfinzi” OR “Dostarlimab” OR “GSK4057190” OR “TSR-042” OR “Cemiplimab” OR ”REGN2810” OR “Avelumab” OR “MSB-0010682” OR “MSB0010682” OR ”bavencio” OR ”MSB0010718C” OR ”MSB-0010718C” OR “Atezolizumab” OR “MPDL3280A” OR ”MPDL-3280A” OR “Tecentriq” OR “RG7446” OR “RG-7446” OR “pidilizumab” OR “CT-011” OR “CT 011” OR “tremelimumab” OR “ticilimumab” OR “CP 675” OR “CP675 cpd” OR “CP-675” OR “CP-675,206” OR “CP-675206” OR “CP675206” OR “CP 675206” OR “sintilimab” OR “SHR-1210” OR “camrelizumab” OR “toripalimab” OR “HX008” OR “tremelimumab”) | 59,780 |
|  | #3 | TITLE-ABS-KEY(“RCT” OR “trial*” OR “intervention*” OR “placebo” OR “randomised trial*” OR “randomized trial*” OR “controlled trial*” OR "Random Allocation” OR "Single-Blind" OR "Double-Blind" OR "Cross-Over" OR "Clinical Trial*" OR "Controlled Clinical Trial" OR "Non-Randomized Controlled Trial*") | 4,358,287 |
|  | #4 | #1 AND #2 AND #3 | 1,865 |
| Web of Science  (8.19.2022) | #1 | TS=(Prostatic OR prostate OR “prostate neoplasm*” OR “prostatic neoplasm*” OR “prostate cancer*” OR “prostatic cancer*” OR “cancer of prostate” OR “neoplasm of prostate” OR “cancer of the prostate” OR “neoplasm of the prostate” OR “adenocarcinoma of prostate” OR “adenocarcinoma of the prostate” OR “squamous cell carcinoma of prostate” OR “squamous cell carcinoma of the prostate” OR “transitional cell carcinoma of prostate” OR “transitional cell carcinoma of the prostate” OR “cancers of prostate” OR “neoplasms of prostate” OR “cancers of the prostate” OR “neoplasms of the prostate” OR “adenocarcinomas of prostate” OR “adenocarcinomas of the prostate” OR “squamous cell carcinomas of prostate” OR “squamous cell carcinomas of the prostate” OR “transitional cell carcinomas of prostate” OR “transitional cell carcinomas of the prostate” OR “Castration-Resistant Prostate Neoplasm*” OR “Castration Resistant Prostate Neoplasm*” OR “Castration-Resistant Prostate Cancer*” OR “Castration Resistant Prostate cancer*” OR “Androgen-Resistant Prostate Neoplasm*” OR “Androgen Resistant Prostate Neoplasm*” OR “Androgen-Resistant Prostate Cancer*” OR “Androgen Resistant Prostate cancer*” OR “Androgen-Insensitive Prostate Neoplasm*” OR “Androgen Insensitive Prostate Neoplasm*” OR “Androgen-Insensitive Prostate Cancer*” OR “Androgen Insensitive Prostate cancer*” OR “Androgen-Independent Prostate Neoplasm*” OR “Androgen Independent Prostate Neoplasm*” OR “Androgen-Independent Prostate Cancer*” OR “Androgen Independent Prostate cancer*” OR “Hormone refractory Prostate Neoplasm*” OR “Hormone refractory Prostate Neoplasm*” OR “Hormone refractory Prostate Cancer*” OR “Hormone refractory Prostate cancer*” OR “Castration-Resistant Prostatic Neoplasm*” OR “Castration Resistant Prostatic Neoplasm*” OR “Castration-Resistant Prostatic Cancer*” OR “Castration Resistant Prostatic cancer*” OR “Androgen-Resistant Prostatic Neoplasm*” OR “Androgen Resistant Prostatic Neoplasm*” OR “Androgen-Resistant Prostatic Cancer*” OR “Androgen Resistant Prostatic cancer*” OR “Androgen-Insensitive Prostatic Neoplasm*” OR “Androgen Insensitive Prostatic Neoplasm*” OR “Androgen-Insensitive Prostatic Cancer*” OR “Androgen Insensitive Prostatic cancer*” OR “Androgen-Independent Prostatic Neoplasm*” OR “Androgen Independent Prostatic Neoplasm*” OR “Androgen-Independent Prostatic Cancer*” OR “Androgen Independent Prostatic cancer*” OR “Hormone refractory Prostatic Neoplasm*” OR “Hormone refractory Prostatic Neoplasm*” OR “Hormone refractory Prostatic Cancer*” OR “Hormone refractory Prostatic cancer*”) | 336,544 |
|  | #2 | TS=(“Immune Checkpoint Inhibitor*” OR “Immune Checkpoint Blocker*” OR “Immune Checkpoint Blockade*” OR “Immune Checkpoint Inhibition*” OR “PD-L1 Inhibitor*” OR “PD L1 Inhibitor*” OR “PDL1 Inhibitor*” OR “PD-L1 blocker*” OR “PD L1 blocker*” OR “PDL1 blocker*” OR “Programmed Death-Ligand 1 Inhibitor*” OR “Programmed Death Ligand 1 Inhibitor*” OR “Programmed Death-Ligand 1 blocker*” OR “Programmed Death Ligand 1 blocker*” OR “anti Programmed Death-Ligand 1” OR “anti-Programmed Death-Ligand 1” OR “anti Programmed Death Ligand 1” OR “anti-Programmed Death Ligand 1” OR “CTLA-4 Inhibitor*” OR “CTLA 4 Inhibitor*” OR “CTLA4 Inhibitor*” OR “CTLA-4 blocker*” OR “CTLA 4 blocker*” OR “CTLA4 blocker*” OR “Cytotoxic T-Lymphocyte Associated Protein 4 Inhibitor*” OR “Cytotoxic T Lymphocyte-Associated Protein 4 Inhibitor*” OR “Cytotoxic T-Lymphocyte Associated Protein 4 blocker*” OR “Cytotoxic T Lymphocyte-Associated Protein 4 blocker*” OR “Cytotoxic T-Lymphocyte Associated antigen 4 Inhibitor*” OR “Cytotoxic T Lymphocyte-Associated antigen 4 Inhibitor*” OR “Cytotoxic T-Lymphocyte Associated antigen 4 blocker*” OR “Cytotoxic T Lymphocyte-Associated antigen 4 blocker*” OR “Cytotoxic T-Lymphocyte-Associated Protein 4 Inhibitor*” OR “Cytotoxic T Lymphocyte Associated Protein 4 Inhibitor*” OR “Cytotoxic T-Lymphocyte-Associated Protein 4 blocker*” OR “Cytotoxic T Lymphocyte Associated Protein 4 blocker*” OR “Cytotoxic T-Lymphocyte-Associated antigen 4 Inhibitor*” OR “Cytotoxic T Lymphocyte Associated antigen 4 Inhibitor*” OR “Cytotoxic T-Lymphocyte-Associated antigen 4 blocker*” OR “Cytotoxic T Lymphocyte Associated antigen 4 blocker*” OR “anti-cytotoxic T lymphocyte-associated antigen 4” OR “anti cytotoxic T lymphocyte-associated antigen 4” OR “anti-cytotoxic T lymphocyte-associated protein 4” OR “anti cytotoxic T lymphocyte-associated protein 4” OR “anti-cytotoxic T-lymphocyte-associated antigen 4” OR “anti-cytotoxic T-lymphocyte-associated antigen 4” OR “PD-1 Inhibitor*” OR “PD 1 Inhibitor*” OR “PD1 Inhibitor*” OR “PD 1 blocker*” OR “PD1 blocker*” OR “PD-1 blocker*” OR “Programmed Cell Death Protein 1 Inhibitor*” OR “Programmed Cell Death Protein 1 blocker*” OR “anti Programmed Cell Death Protein 1” OR “anti-Programmed Cell Death Protein 1” OR “anti-PD1” OR “anti PD1” OR “anti-PD-1” OR “anti PD-1” OR “anti-PD-L1” OR “anti PDL1” OR “anti PD-L1” OR “anti-PDL1” OR “anti-PD L1” OR “anti PD L1” OR “anti-CTLA4” OR “anti-CTLA 4” OR “anti-CTLA-4” OR “anti CTLA4” OR “anti CTLA 4” OR “anti CTLA-4” OR “PD-1-PD-L1 Blockade*” OR “PD 1 PD L1 Blockade*” OR “Pembrolizumab” OR “MK-3475” OR “lambrolizumab” OR “Keytruda” OR “SCH-900475” OR “Nivolumab” OR “Opdivo” OR “ONO-4538” OR “ONO 4538” OR “ONO4538” OR “MDX-1106” OR “MDX 1106” OR “MDX1106” OR “BMS-936558” OR “BMS 936558” OR “BMS936558” OR “Ipilimumab” OR “Yervoy” OR “MDX 010” OR “MDX010” OR “MDX-010” OR “MDX-CTLA-4” OR “MDX CTLA 4” OR “Durvalumab” OR “MEDI4736” OR “MEDI-4736” OR “Imfinzi” OR “Dostarlimab” OR “GSK4057190” OR “TSR-042” OR “Cemiplimab” OR ”REGN2810” OR “Avelumab” OR “MSB-0010682” OR “MSB0010682” OR ”bavencio” OR ”MSB0010718C” OR ”MSB-0010718C” OR “Atezolizumab” OR “MPDL3280A” OR ”MPDL-3280A” OR “Tecentriq” OR “RG7446” OR “RG-7446” OR “pidilizumab” OR “CT-011” OR “CT 011” OR “tremelimumab” OR “ticilimumab” OR “CP 675” OR “CP675 cpd” OR “CP-675” OR “CP-675,206” OR “CP-675206” OR “CP675206” OR “CP 675206” OR “sintilimab” OR “SHR-1210” OR “camrelizumab” OR “toripalimab” OR “HX008” OR “tremelimumab”) | 60,063 |
|  | #3 | TS=(“RCT” OR “trial*” OR “intervention*” OR “placebo” OR “randomised trial*” OR “randomized trial*” OR “controlled trial*” OR "Random Allocation” OR "Single-Blind" OR "Double-Blind" OR "Cross-Over" OR "Clinical Trial*" OR "Controlled Clinical Trial" OR "Non-Randomized Controlled Trial*") | 3,283,784 |
|  | #4 | #1 AND #2 AND #3 | 797 |
| EMBASE  (8.19.2022) | #1 | (Prostatic OR prostate OR “prostate neoplasm*” OR “prostatic neoplasm*” OR “prostate cancer*” OR “prostatic cancer*” OR “cancer of prostate” OR “neoplasm of prostate” OR “cancer of the prostate” OR “neoplasm of the prostate” OR “adenocarcinoma of prostate” OR “adenocarcinoma of the prostate” OR “squamous cell carcinoma of prostate” OR “squamous cell carcinoma of the prostate” OR “transitional cell carcinoma of prostate” OR “transitional cell carcinoma of the prostate” OR “cancers of prostate” OR “neoplasms of prostate” OR “cancers of the prostate” OR “neoplasms of the prostate” OR “adenocarcinomas of prostate” OR “adenocarcinomas of the prostate” OR “squamous cell carcinomas of prostate” OR “squamous cell carcinomas of the prostate” OR “transitional cell carcinomas of prostate” OR “transitional cell carcinomas of the prostate” OR “Castration-Resistant Prostate Neoplasm*” OR “Castration Resistant Prostate Neoplasm*” OR “Castration-Resistant Prostate Cancer*” OR “Castration Resistant Prostate cancer*” OR “Androgen-Resistant Prostate Neoplasm*” OR “Androgen Resistant Prostate Neoplasm*” OR “Androgen-Resistant Prostate Cancer*” OR “Androgen Resistant Prostate cancer*” OR “Androgen-Insensitive Prostate Neoplasm*” OR “Androgen Insensitive Prostate Neoplasm*” OR “Androgen-Insensitive Prostate Cancer*” OR “Androgen Insensitive Prostate cancer*” OR “Androgen-Independent Prostate Neoplasm*” OR “Androgen Independent Prostate Neoplasm*” OR “Androgen-Independent Prostate Cancer*” OR “Androgen Independent Prostate cancer*” OR “Hormone refractory Prostate Neoplasm*” OR “Hormone refractory Prostate Neoplasm*” OR “Hormone refractory Prostate Cancer*” OR “Hormone refractory Prostate cancer*” OR “Castration-Resistant Prostatic Neoplasm*” OR “Castration Resistant Prostatic Neoplasm*” OR “Castration-Resistant Prostatic Cancer*” OR “Castration Resistant Prostatic cancer*” OR “Androgen-Resistant Prostatic Neoplasm*” OR “Androgen Resistant Prostatic Neoplasm*” OR “Androgen-Resistant Prostatic Cancer*” OR “Androgen Resistant Prostatic cancer*” OR “Androgen-Insensitive Prostatic Neoplasm*” OR “Androgen Insensitive Prostatic Neoplasm*” OR “Androgen-Insensitive Prostatic Cancer*” OR “Androgen Insensitive Prostatic cancer*” OR “Androgen-Independent Prostatic Neoplasm*” OR “Androgen Independent Prostatic Neoplasm*” OR “Androgen-Independent Prostatic Cancer*” OR “Androgen Independent Prostatic cancer*” OR “Hormone refractory Prostatic Neoplasm*” OR “Hormone refractory Prostatic Neoplasm*” OR “Hormone refractory Prostatic Cancer*” OR “Hormone refractory Prostatic cancer*”):ab,ti | 342,405 |
|  | #2 | (“Immune Checkpoint Inhibitor*” OR “Immune Checkpoint Blocker*” OR “Immune Checkpoint Blockade*” OR “Immune Checkpoint Inhibition*” OR “PD-L1 Inhibitor*” OR “PD L1 Inhibitor*” OR “PDL1 Inhibitor*” OR “PD-L1 blocker*” OR “PD L1 blocker*” OR “PDL1 blocker*” OR “Programmed Death-Ligand 1 Inhibitor*” OR “Programmed Death Ligand 1 Inhibitor*” OR “Programmed Death-Ligand 1 blocker*” OR “Programmed Death Ligand 1 blocker*” OR “anti Programmed Death-Ligand 1” OR “anti-Programmed Death-Ligand 1” OR “anti Programmed Death Ligand 1” OR “anti-Programmed Death Ligand 1” OR “CTLA-4 Inhibitor*” OR “CTLA 4 Inhibitor*” OR “CTLA4 Inhibitor*” OR “CTLA-4 blocker*” OR “CTLA 4 blocker*” OR “CTLA4 blocker*” OR “Cytotoxic T-Lymphocyte Associated Protein 4 Inhibitor*” OR “Cytotoxic T Lymphocyte-Associated Protein 4 Inhibitor*” OR “Cytotoxic T-Lymphocyte Associated Protein 4 blocker*” OR “Cytotoxic T Lymphocyte-Associated Protein 4 blocker*” OR “Cytotoxic T-Lymphocyte Associated antigen 4 Inhibitor*” OR “Cytotoxic T Lymphocyte-Associated antigen 4 Inhibitor*” OR “Cytotoxic T-Lymphocyte Associated antigen 4 blocker*” OR “Cytotoxic T Lymphocyte-Associated antigen 4 blocker*” OR “Cytotoxic T-Lymphocyte-Associated Protein 4 Inhibitor*” OR “Cytotoxic T Lymphocyte Associated Protein 4 Inhibitor*” OR “Cytotoxic T-Lymphocyte-Associated Protein 4 blocker*” OR “Cytotoxic T Lymphocyte Associated Protein 4 blocker*” OR “Cytotoxic T-Lymphocyte-Associated antigen 4 Inhibitor*” OR “Cytotoxic T Lymphocyte Associated antigen 4 Inhibitor*” OR “Cytotoxic T-Lymphocyte-Associated antigen 4 blocker*” OR “Cytotoxic T Lymphocyte Associated antigen 4 blocker*” OR “anti-cytotoxic T lymphocyte-associated antigen 4” OR “anti cytotoxic T lymphocyte-associated antigen 4” OR “anti-cytotoxic T lymphocyte-associated protein 4” OR “anti cytotoxic T lymphocyte-associated protein 4” OR “anti-cytotoxic T-lymphocyte-associated antigen 4” OR “anti-cytotoxic T-lymphocyte-associated antigen 4” OR “PD-1 Inhibitor*” OR “PD 1 Inhibitor*” OR “PD1 Inhibitor*” OR “PD 1 blocker*” OR “PD1 blocker*” OR “PD-1 blocker*” OR “Programmed Cell Death Protein 1 Inhibitor*” OR “Programmed Cell Death Protein 1 blocker*” OR “anti Programmed Cell Death Protein 1” OR “anti-Programmed Cell Death Protein 1” OR “anti-PD1” OR “anti PD1” OR “anti-PD-1” OR “anti PD-1” OR “anti-PD-L1” OR “anti PDL1” OR “anti PD-L1” OR “anti-PDL1” OR “anti-PD L1” OR “anti PD L1” OR “anti-CTLA4” OR “anti-CTLA 4” OR “anti-CTLA-4” OR “anti CTLA4” OR “anti CTLA 4” OR “anti CTLA-4” OR “PD-1-PD-L1 Blockade*” OR “PD 1 PD L1 Blockade*” OR “Pembrolizumab” OR “MK-3475” OR “lambrolizumab” OR “Keytruda” OR “SCH-900475” OR “Nivolumab” OR “Opdivo” OR “ONO-4538” OR “ONO 4538” OR “ONO4538” OR “MDX-1106” OR “MDX 1106” OR “MDX1106” OR “BMS-936558” OR “BMS 936558” OR “BMS936558” OR “Ipilimumab” OR “Yervoy” OR “MDX 010” OR “MDX010” OR “MDX-010” OR “MDX-CTLA-4” OR “MDX CTLA 4” OR “Durvalumab” OR “MEDI4736” OR “MEDI-4736” OR “Imfinzi” OR “Dostarlimab” OR “GSK4057190” OR “TSR-042” OR “Cemiplimab” OR ”REGN2810” OR “Avelumab” OR “MSB-0010682” OR “MSB0010682” OR ”bavencio” OR ”MSB0010718C” OR ”MSB-0010718C” OR “Atezolizumab” OR “MPDL3280A” OR ”MPDL-3280A” OR “Tecentriq” OR “RG7446” OR “RG-7446” OR “pidilizumab” OR “CT-011” OR “CT 011” OR “tremelimumab” OR “ticilimumab” OR “CP 675” OR “CP675 cpd” OR “CP-675” OR “CP-675,206” OR “CP-675206” OR “CP675206” OR “CP 675206” OR “sintilimab” OR “SHR-1210” OR “camrelizumab” OR “toripalimab” OR “HX008” OR “tremelimumab”):ab,ti | 74,501 |
|  | #3 | (“RCT” OR “trial*” OR “intervention*” OR “placebo” OR “randomised trial*” OR “randomized trial*” OR “controlled trial*” OR "Random Allocation” OR "Single-Blind" OR "Double-Blind" OR "Cross-Over" OR "Clinical Trial*" OR "Controlled Clinical Trial" OR "Non-Randomized Controlled Trial*"):ab,ti | 3,356,739 |
|  | #4 | #1 AND #2 AND #3 | 818 |

**Table S2.** Treatment dose and schedules of included studies.

| First author | Year of publication | ICI name | Target | Dose of ICI | Schedule of ICI | Concomitant treatments | Dose of concomitant treatments | Schedule of concomitant treatments |
| --- | --- | --- | --- | --- | --- | --- | --- | --- |
| Agarwal et al. (1) | 2022 | Atezolizumab | PD-L1 | 1200 mg | Q3W/IV | Cabozantinib | 40 mg | QD/Orally |
| Alva et al. (2) | 2022 | Nivolumab and Ipilimumab | PD-1/CTLA-4 | Nivolumab: 3 mg/kg Ipilimumab: 1 mg/kg | Q3W for four cycles, followed by maintenance Nivolumab at 480 mg Q4W/IV | NA | NA | NA |
| Antonarakis (1) et al. (3) | 2019 | Pembrolizumab | PD-1 | 200 mg | Q3W for up to 35 cycles/IV | NA | NA | NA |
| Antonarakis (2) et al. (4) | 2021 | Pembrolizumab | PD-1 | 200 mg | Q3W for up to 35 cycles/IV | NA | NA | NA |
| Boudadi et al. (5) | 2018 | Nivolumab and Ipilimumab | PD-1/CTLA-4 | Nivolumab: 3 mg/kg Ipilimumab: 1 mg/kg | Q3W for four doses, followed by a maintenance regimen of 3 mg/Kg of Nivolumab Q2W/IV | NA | NA | NA |
| Brown et al. (6) | 2022 | Avelumab | PD-L1 | 10 mg/kg | Q2W/IV | NA | NA | NA |
| Fizazi (1) et al. (7) | 2021 | Nivolumab | PD-1 | 480 mg | Q4W/IV | Rucaparib | 600 mg | BID/Orally |
| Fizazi (1) et al. (8) | 2022 | Nivolumab | PD-1 | 360 mg | Q3W/IV | Docetaxel | 75 mg/m2 | Q3W for a maximum of 10 cycles, followed by Nivolumab 480 mg Q4W for 2 years/IV |
| Fakhrejahani et al. (9) | 2017 | Avelumab | PD-L1 | 10 mg/kg | Q2W/IV | NA | NA | NA |
| Graff (1) et al. (10) | 2019 | Ipilimumab | CTLA-4 | 10 mg/kg | Q3W for four doses with maintenance Ipilimumab Q12W/IV | NA | NA | NA |
| Fong et al. (11) | 2021 | Atezolizumab | PD-L1 | 840 mg | Day 1 and day 15 of each 28-day cycle/IV | Radium-223 | 55 kBq/kg | Day 1 of each 28-day cycle at a maximum of six times/IV |
| Graff (2) et al. (12) | 2020 | Pembrolizumab | PD-1 | 200 mg | Q3W for four doses/IV | Enzalutamide | NA | NA |
| Hansen et al. (13) | 2018 | Pembrolizumab | PD-1 | 10 mg/kg | Q2W/IV | NA | NA | NA |
| Hotte et al. (14) | 2019 | Durvalumab and Tremelimumab | PD-L1/CTLA-4 | Durvalumab: 1500 mg Trememumab: 75 mg | Durvalumab Q4W/IV 4 doses of Trememumab/IV | NA | NA | NA |
|  |  | Durvalumab | PD-L1 | Durvalumab 1500 mg | Q4W/IV |  |  |  |
| Howard et al. (15) | 2022 | Pembrolizumab | PD-1 | 200 mg | Q3W/IV | Olaparib | 400 mg Capsule/ 300 mg tablet | BID/Orally |
|  |  |  |  |  |  | Docetaxel | 75 mg | Q3W/IV |
|  |  |  |  |  |  | Enzalutamide | 160 mg | QD/Orally |
| Karzai et al. (16) | 2018 | Durvalumab | PD-L1 | 1500 mg | Every 28 days/IV | Olaparib | 300 mg | BID on a 28-day cycle/Orally |
| Kwan et al. (17) | 2021 | Avelumab | PD-1 | 10 mg/kg | Q3W for 12 cycles/IV | Stereotactic Ablative Body Radiotherapy (SABR) | 20 Gy | A single fraction of SABR was administered to one or two disease sites within 5 d before the first and second Avelumab treatments |
| Kwon et al. (18) | 2014 | Ipilimumab | CTLA-4 | 10 mg/kg | Q3W for four doses/IV | NA | NA | NA |
| Layton et al. (19) | 2021 | Avelumab | PD-L1 | 10 mg/kg | Q2W/IV | Next generation hormonal therapies (NHTs) | NA | NA |
| Markowski et al. (20) | 2021 | Nivolumab | PD-1 | 480 mg | Every 28 days/IV | Testosterone cypionate (BAT) | 400 mg | Every 28 days/IM |
| Mourey et al. (21) | 2020 | Pembrolizumab | PD-1 | 200 mg | Q3W/IV | Enzalutamide | 160 mg | QD/Orally |
| Petrylak et al. (22) | 2021 | Atezolizumab | PD-1 | 1200 mg | Q3W/IV | NA | NA | NA |
| Piulats et al. (23) | 2021 | pembrolizumab | PD-1 | 200 mg | Q3W/IV | Abiraterone acetate | 1000 mg | QD/Orally |
| Beer et al. (24) | 2017 | Ipilimumab | CTLA-4 | 10 mg/kg | Q3W for four doses followed by maintenance treatment with Ipilimumab 10 mg/kg Q12W/IV | NA | NA | NA |
| Powles et al. (25) | 2022 | Atezolizumab | PD-L1 | 1200 mg | Q3W/IV | Enzalutamide | 160 mg | QD/Orally |
| Rodriguez-Vida et al. (26) | 2021 | Avelumab | PD-L1 | 10mg/kg | NA | Carboplatin | NA | 2 cycles of Carboplatin followed by 2 cycles of Avelumab plus Carboplatin and by maintenance Avelumab for 2 years/IV |
| Sharma et al. (27) | 2020 | Nivolumab and Ipilimumab | PD-1/CTLA-4 | Nivolumab: 1 mg/kg Ipilimumab: 3 mg/kg | Q3W for up to four doses, followed by 480 mg Nivolumab Q4W/IV | NA | NA | NA |
| Shenderov et al. (28) | 2021 | Nivolumab and Ipilimumab | PD-1/CTLA-4 | Nivolumab: 3 mg/kg Ipilimumab: 1 mg/kg | Q3W for 4 doses, followed by a maintenance regimen of 3 mg/kg of Nivolumab Q2W/IV | NA | NA | NA |
|  |  | Nivolumab and Ipilimumab | PD-1/CTLA-4 | Nivolumab: 3 mg/kg Ipilimumab: 1 mg/kg | Q3W for 4 doses, followed by a maintenance regimen of 3 mg/kg of Nivolumab Q2W/IV | Enzalutamide | NA | NA |
| Small et al. (29) | 2007 | Ipilimumab | CTLA4 | 3mg/kg | Single IV dose | NA | NA | NA |
| Subudhi et al. (30) | 2020 | Ipilimumab | CTLA-4 | 3 mg/kg | Q3W for four doses/IV | NA | NA | NA |
| Vaishampayan et al. (31) | 2020 | Pembrolizumab | PD-1 | 200 mg | Q3W for 35 cycles/IV | Enzalutamide |  | QD |
| Yu (1) et al. (32) | 2021 | Pembrolizumab | PD-1 | 200 mg | Q3W/IV | Olaparib | 400-mg capsule or 300-mg tablet | BID/Orally |
| Slovin et al. (33) | 2013 | Ipilimumab | CTLA-4 | 3 mg/kg | Q3W for four doses/IV | NA | NA | NA |
|  |  |  |  | 3 mg/kg |  | External-beam radiotherapy (XRT) |  |  |
|  |  |  |  | 5 mg/kg |  | NA |  |  |
|  |  |  |  | 10 mg/kg |  | NA |  |  |
|  |  |  |  | 10 mg/kg |  | XRT |  |  |
|  |  |  |  | 10 mg/kg |  | XRT |  |  |
| Zucali et al. (34) | 2022 | Cemiplimab | PD-1 | 350 mg | Q3W/IV | Isatuximab | 10 mg/kg | QW for 3 weeks (one cycle, 21 days) followed by Q3W/IV |
| Yu (2) et al. (35) | 2022 | Pembrolizumab | PD-1 | 200 mg | Q3W for 35 cycles/IV | Docetaxel | 75 mg/m2 | Q3W for ten cycles/IV |

**Table S3.** Criteria of enrolling patients reported in the included studies.

| **First author** | **Criteria for enrolling patients** |
| --- | --- |
| Agarwal et al. (1) | 1) Primary histology of adenocarcinoma without small-cell features, 2) measurable soft tissue disease per Response Evaluation Criteria In Solid Tumors (RECIST) version 1.1,14, 3) an ECOG PS of 0 or 1, 4) serum testosterone concentration less than 1·73 nm/L.  PSA or bone progression alone were not sufficient for enrolment. Chemotherapy in the castrate-resistant setting was not allowed, but previous docetaxel in combination with androgen deprivation therapy for castration-sensitive disease was permitted. Previous treatment with cabozantinib or an ICI was not allowed.  Key exclusion criteria included concurrent treatment with oral anticoagulants (excluding low-dose aspirin and low-dose low molecular-weight heparins); presence of untreated brain metastases; and uncontrolled, clinically significant comorbidities, including cardiovascular and gastrointestinal disorders. |
| Alva et al. (2) | Eligible pts had mCRPC (ongoing androgen deprivation therapy with serum testosterone ≤50 ng/dL) and putative CDK12 inactivation of function aberrations on any commercial or institutional CLIA/CAP approved next generation sequencing assay. |
| Antonarakis (1) et al. (3) | 1) Age 18 years or older, 2) metastatic or locally confined but inoperable, pathologically confirmed prostate adenocarcinoma, 3) measurable disease per RECIST v1.133 (cohorts 1 and 2) or detectable bone metastases by whole-body bone scintigraphy and no RECIST-measurable tumors (cohort 3) by central review, 4) Eastern Cooperative Oncology Group performance status 0, 1, or 2, 5) provision of a tumor sample for PD-L1 assessment (cohort 1 limited to PD-L1–positive disease, cohort 2 limited to PD-L1–negative disease), and 6) previous treatment with one or more targeted endocrine therapies and one to two chemotherapy regimens, one of which must have included docetaxel. |
| Antonarakis (2) et al. (4) | Three cohorts of patients (C1: RECIST-measurable, PD-L1 positive; C2: RECIST-measurable, PD-L1 negative; C3: bone-predominant irrespective of PD-L1). |
| Boudadi et al. (5) | 1) Histologically confirmed, progressive, metastatic castration-resistant prostate cancer (mCRPC) with detectable AR-V7 transcripts using the Johns Hopkins CTC-based clinical-grade ARV7 assay, 2) an ECOG performance-status of 0-1, 3) at least 18 years of age, 4) serum testosterone <50 ng/dL with ongoing androgen-deprivation therapy, 5) adequate organ (liver, kidney, bone marrow) function, and 6) availability of new or archival tumor tissue for biomarker analysis.  Key exclusion criteria included a second active malignancy within 5 years, prior ICI therapy, active brain or meningeal metastases, history of autoimmune disease, or requirement for systemic corticosteroids. |
| Brown et al. (6) | 1) Diagnosed with neuroendocrine or neuroendocrine-like prostate cancer based on histology criteria or clinical presentation criteria of AVPC. To meet histologic criteria patients needed to harbor either primary small cell carcinoma of the prostate, intermediate atypical carcinoma of the prostate, or mixed histology containing both adenocarcinoma and neuroendocrine or small cell components. To meet clinical presentation inclusion criteria of AVPC with adenocarcinoma of the prostate, but without any sign of neuroendocrine or small cell histology, patients had to be radiographically progressing despite castrate levels of testosterone (< 50 ng/ml) and have the following poor risk features: (1) Prior progression despite therapy with either abiraterone acetate and/or enzalutamide. (2) At least one of the following: (1) liver metastases; (2) bulky radiographic progression (> 2 cm short axis lymph nodes or > 1 cm long axis visceral metastases) combined with low serum PSA (<10 ng/ml);  (3) high serum LDH (>1X upper limit of normal (ULN)). 2) Required to have received one line of approved chemotherapy and/or hormonal therapy and could have received up to three prior chemotherapy regimens. Patients who previously received anti-PD(L)1 or anti-CTLA4 agents were excluded. Additional key exclusion criteria include active ongoing immunologic or autoimmune disease, immunosuppressant medication, prior organ or allogeneic stem-cell transplantation or active HIV, hepatitis B, or hepatitis C infection, active cardiovascular disease, persistent toxicity (>Grade 1) related to prior therapy, or other severe acute or chronic medical conditions such as colitis, inflammatory bowel disease, pneumonitis, or pulmonary fibrosis. |
| Fizazi (1) et al. (7) | 1) Adult patients (≥18 years of age), 2) histological confirmation of adenocarcinoma of the prostate with radiologic evidence of stage IV disease (N1 and/or M1), 3) ongoing androgen deprivation therapy or bilateral orchiectomy (confirmed by testosterone level ≤1.73nmol/L at screening), 4) documented progressive disease per Prostate Cancer Clinical Trials Working Group 3 (PCWG3) criteria, 5) an Eastern Cooperative Oncology Group performance status of 0 or 1, and 6) sufficient tumor tissue obtained within 5 years before enrollment from a metastatic or primary tumor lesion not previously irradiated.  Exclusion criteria included active brain metastases, conditions requiring systemic treatment with corticosteroids (>10mg daily prednisone equivalent) or other immunosuppressive medications within 14 days of start of study treatment, and prior therapy specifically targeting T-cell costimulation or immune checkpoint pathways.For assignment to cohort A1, patients must have received 1–2 prior taxane-based chemotherapy regimens in the castration-resistant setting, and prior treatment with up to two novel hormonal therapies. Patients were excluded from both cohorts A1 and A2 if they had myelodysplastic syndrome/acute myeloid leukemia, gastrointestinal disorders likely to interfere with absorption of study treatment, and/or had received previous treatment with a PARP inhibitor, mitoxantrone, cyclophosphamide, or platinum-based chemotherapy. |
| Fizazi (1) et al. (8) | 1) adults with histologically confirmed adenocarcinoma of the prostate with radiologic evidence of M1 metastatic disease, 2) ongoing androgen deprivation therapy with a gonadotropinreleasing hormone analogue or bilateral orchiectomy (confirmed by a testosterone level 1.73 nmol/L at screening), 3) documented prostate cancer progression per Prostate Cancer Clinical Trials Working Group 3 (PCWG3) criteria, 4) an ECOG PD of 0-1, and 5) sufficient tumour tissue obtained within 5 years before enrolment from a metastatic tumour lesion or primary tumour lesion not previously irradiated.  The exclusion criteria included active brain metastases, conditions requiring systemic corticosteroids (>10 mg daily prednisone equivalent) or other immunosuppressive medications within 14 days of the start of study treatment and prior treatments specifically targeting T-cell co-stimulation or checkpoint pathways. For assignment to cohort B, patients had to have chemotherapy-naive mCRPC and be candidates for immediate docetaxel treatment (per the investigator’s discretion). Prior treatment with up to 2 novel hormonal therapies (NHTs; i.e. abiraterone, enzalutamide or apalutamide) in the castration-resistant setting was permissible if the last dose was administered >28 days before cohort assignment. Patients who previously received docetaxel or another chemotherapy for mCRPC were excluded, although prior docetaxel treatment for metastatic hormone-sensitive prostate cancer was allowed if 12 months had elapsed from the last docetaxel dose. Patients with grade 2 peripheral neuropathy (per National Cancer Institute Common Terminology Criteria for Adverse Events [CTCAE] version 4.03) were also excluded from cohort B. |
| Fakhrejahani et al. (9) | mCRPC patients who had progressive disease on previous treatment. Patients who had progressive disease on an androgen receptor antagonist (ARA) could enroll on trial and continue their ARA. |
| Graff (1) et al. (10) | 1) At least 18 years of age, 2) an ECOG PS 0 or 1, 3) histologically confirmed adenocarcinoma of the prostate, 4) castrate levels of testosterone (<50 ng/mL), and 5) a PSA level of >0.2 ng/mL after 6–18 months of androgen deprivation therapy (ADT) utilizing a luteinizing hormone-releasing hormone (LHRH) agonist/antagonist with or without the use of an antiandrogen. ADT was continued throughout the study. If an anti-androgen were stopped prior to enrollment, then it had to be stopped 4 weeks prior to enrollment for nilutamide and flutamide and 6 weeks prior for bicalutamide to ensure an appropriate washout period. All patients had radiographic evidence of distant or regional metastasis at the time of enrollment as detected by computed tomography and/or Technetium-99 bone scan. Exclusion criteria included prior treatment with ipilimumab or any other CTLA-4 targeting agent (e.g., CD137 agonist) or concomitant therapy with any non-study immunomodulatory agent, radiation therapy to any area of the body within 28 days of enrollment, other active malignancies or autoimmune disorders, leucopenia, neutropenia, platelets <50 × 103/uL, hemoglobin <8 g/dL, creatinine >3.0 × ULN, AST/ALT >2.5 × ULN. |
| Fong et al. (11) | 1) 18 years of age or older, 2) measurable disease per RECIST 1.1, 3) known progressive mCRPC defined as castrate serum testosterone level ≤ 50 ng/dL (1.7 nmol/L), bilateral orchiectomy or maintenance on androgen ablation therapy with luteinizing hormone-releasing agonist or antagonist or polyestradiol phosphate throughout study and follow-up period, serum PSA progression, and serum PSA ≥ 2 ng/mL (disease progression was defined according to Prostate Cancer Working Group 2 (PCWG2) criteria during or following treatment with at least 28 days of treatment with a second-generation androgen pathway inhibitor), 4) history of treatment with a taxane-containing regimen or ineligibility/refusal of a taxanecontaining regimen, 5) required to have two or more bone metastases and visceral metastases or malignant lymphadenopathy, 6) having disease that was not amenable to curative or locoregional therapies or had progressed thereafter, 7) an ECOG PS of 0 or 1, and 8) adequate hematologic and organ function. Key exclusion criteria included a history of autoimmune disease, significant liver disease, prior radionuclide therapy, coinfection with hepatitis B and hepatitis C virus, and malignancies other than CRPC within 5 years prior to initiation of study treatment. |
| Graff (2) et al. (12) | Metastatic castration-resistant prostate cancer patients progressing on enzalutamide alone by prostate-specific antigen (PSA) and/or imaging studies. Subjects could have previously received sipuleucel-T and could have received abiraterone or chemotherapy for castration-sensitive disease, but previous treatment with PD-1, PD-L1 or CTLA-4 antibodies for any disease state was prohibited. Furthermore, subjects with a history of autoimmune disease or pneumonitis were excluded. |
| Hansen et al. (13) | 1) Aged 18 years, 2) histologically or cytologically documented, locally advanced or metastatic prostate adenocarcinoma that was incurable and for which standard therapy was ineffective or not considered appropriate, 3) at least one measurable lesion at baseline [irrespective of prostate-specific antigen (PSA) levels] after last line of cancer therapy based on Response Evaluation Criteria in Solid Tumors, version 1.1 (RECIST v1.1), 4) PD-L1 expression in 1% of tumor or stromal cells, 4) ECOG PS 0/1, and 5) adequate organ function, determined by laboratory testing within 10 days of first pembrolizumab dose. Patients with prostate cancer who were receiving luteinizing hormone-releasing hormone (LHRH) analogs were eligible for this study and could continue their therapy at the investigator’s discretion. |
| Hotte et al. (14) | mCRPC patients (measurable disease prior abiraterone and/or enzalutamide, no more than one taxane for mCRPC). |
| Howard et al. (15) | mCRPC patients |
| Karzai et al. (16) | 1) 18 years or older, 2) histopathologically confirmed mCRPC that had progressed after previous treatment with enzalutamide and/or abiraterone, 2) had at least one lesion (soft tissue/viscera or bone) deemed safe to biopsy, 3) ECOG PS of 0–2, and 4) adequate organ function. There were no limitations on previous standard therapies, including previous chemotherapy for mCRPC or metastatic castrationsensitive prostate cancer; however, previous use of ICIs or PARP inhibitors was excluded. Patient selection was not based on mutational status (somatic and/or germline) or other biomarkers. Use of steroids (prednisone or equivalent corticosteroid) exceeding 10 mg/day was not allowed. |
| Kwan et al. (17) | Biochemically/radiographically progressive mCRPC with prior exposure to at least one second-generation androgen receptor (AR) pathway inhibitor. Prior chemotherapy was not mandated, but up to two lines of taxanes were permitted. Patients with any number of metastases were eligible; recruitment was not restricted to patients with oligometastatic disease (five or less metastases). |
| Kwon et al. (18) | 1) Aged 18 years or older, 2) histologically or cytologically confirmed adenocarcinoma of the prostate, 3) at least one bone metastasis that could be irradiated or warranted irradiation in the clinical judgment of the investigator, 4) testosterone concentration less than 1·74 nmol/L, and 5) an ECOG PS of 0 or 1, 6) must have received at least one previous docetaxel-containing regimen for metastatic castrationresistant prostate cancer, consisting of at least two cycles of docetaxel, and progressed while receiving, or within 6 months of receiving, the docetaxel regimen. Disease progression was assessed on the basis of the Prostate Cancer Clinical Trials Working Group’s recommendations.  Patients were excluded if they had received more than two cytotoxic chemotherapy regimens for castration-resistant prostate cancer or if they had brain metastases, an autoimmune disease, or a known HIV, hepatitis B, or hepatitis C infection. |
| Layton et al. (19) | African American men > 18 years of age and had developed mCRPC on next generation hormonal therapies (NHTs). |
| Markowski et al. (20) | mCRPC patients |
| Mourey et al. (21) | Patients who failed or became intolerant to abiraterone acetate after ≥4 wks of treatment in the prechemotherapy mCRPC state and who progressed within 6 months of screening. |
| Petrylak et al. (22) | 1) Prior treatment with sipuleucel-T or enzalutamide for mCRPC, 2) PSA, or radiological disease progression in soft tissue or bone prior to enrollment, 3) ECOG PS of 0 to 1, 4) no history of autoimmune disease, and 5) amenability to metastatic biopsy at screening (pretreatment) and during treatment. |
| Piulats et al. (23) | 1) Had not received second generation hormonal manipulation for mCRPC or failed/were intolerant to enzalutamide for Mcrpc, 2) had progressive disease ≤6 months before screening; and 3) had ECOG PS 0/1. |
| Beer et al. (24) | 1) Confirmed CRPC, 2) radiographic evidence of metastases, 3) prior disease progression during hormonal treatment, 4) discontinuation of prior antiandrogen therapy, 5) ECOG PS of 0 to 1, and 5) testosterone levels , 50 ng/dL. Patients were considered to have minimally symptomatic disease if they rated their 24-h worst pain as # 4 on the Brief Pain Inventory-Short Form scale (1 to 10) in each of the 5 assessment days before random assignment and if they did not require opiate analgesic therapy for cancer-related pain.  Patients were excluded from this study if they had liver, lung, or brain metastases; received prior immunotherapy or chemotherapy for mCRPC; had a history of autoimmune disease; had a HIV or hepatitis B or C infection; or received pelvic-targeted radiation therapy within 3 months of study entry. |
| Powles et al. (25) | 1) Aged ≥18 years, 2) had mCRPC after failure of an androgen synthesis inhibitor (for example, abiraterone acetate) and after failure of, ineligibility for, or refusal of a taxane regimen, 3) not be receiving a hormone receptor inhibitor (for example, enzalutamide), 4) PSA or radiological disease progression in soft tissue or bone before enrollment, and 5) ECOG PS of 0–1. Patients were excluded if they had a history of active autoimmune disease or immune deficiency, coinfection with hepatitis B or hepatitis C virus or previous treatment with immunotherapy, enzalutamide or any other newer AR antagonist. |
| Rodriguez-Vida et al. (26) | Patients with metastatic CRPC progressing to at least 1 taxane and 1 androgen receptor inhibitor. |
| Sharma et al. (27) | 1) At least 18 years of age, 2) histologically confirmed adenocarcinoma of the prostate and evidence of metastatic disease, 3) androgen deprivation therapy (ADT) with a gonadotropin-releasing hormone analogue or bilateral orchiectomy confirmed by testosterone %1.73 nmol/L (50 ng/dL) at screening, 4) soft tissue or bone tumor progression while receiving ADT per Prostate Cancer Working Group 2 (PCWG2) criteria, and 5) an ECOG PS of 0–1.  Key exclusion criteria were visceral liver metastases, active brain or leptomeningeal metastases, autoimmune disease, corticosteroid or immunosuppressant use, and prior treatment targeting T-cell co-stimulation or immune checkpoint pathways. Patients were enrolled into two cohorts: asymptomatic/minimally symptomatic patients progressing after R1 second-generation hormone therapy who had not received chemotherapy for mCRPC (cohort 1), and patients progressing after cytotoxic chemotherapy for mCRPC (cohort 2). |
| Shenderov et al. (28) | 1) Histologically confirmed, progressive, mCRPC, 2) detectable AR‐V7 transcripts using the Johns Hopkins CTC‐based clinical‐grade AR‐V7 assay, 3) an ECOG PS of 0–1, 4) at least 18 years of age, 5) serum testosterone less than 50 ng/dl with ongoing androgen‐deprivation therapy, 6) adequate organ (liver, kidney, bone marrow) function, and 7) availability of new or archival tumor tissue for biomarker analysis. Cohort 1 subjects had to progress through initial hormonal therapy, either by orchiectomy or by using a GnRH agonist in combination with an anti‐androgen. Cohort 2 subjects had to have enzalutamide as their most recent therapy and enzalutamide was continued for study duration despite prior progressive disease.  Key exclusion criteria included a second active malignancy within 5 years, prior ICI therapy, active brain or meningeal metastases, history of autoimmune disease, or requirement for systemic corticosteroids. |
| Small et al. (29) | 1) Histologicallyconfirmed adenocarcinoma of the prostate with evidence of metastatic spread on imaging studies and evidence of disease progression despite androgen deprivation (and if applicable, antiandrogen withdrawal) as defined by the PSA Consensus Criteria, 2) a Karnofsky performance status of ≥60% and an expected survival of at least 3 months, 3) required laboratory tests included adequate hematologic, renal, and hepatic function (WBC ≥1,500/mL, ANC ≥1,500/mL, platelets ≥150*10^3/mL, hematocrit ≥30 %, hemoglobin ≥10 g/dL, creatinine <1.25 upper limit of normal, aspartate aminotransferase <1.25 upper limit of normal, and bilirubin <1.0 upper limit of normal) and serum testosterone <50 ng/mL.  Patients with known autoimmune disorders were excluded, and a negative serum ANA test was required. Prior treatment with secondary hormonal therapies, chemotherapy, or investigational therapy was allowed, provided it was discontinued at least 1 month before treatment, the patient had recovered adequately, and further progressive disease as defined bythe Consensus Criteria was shown. Per Consensus Criteria, an antiandrogen withdrawal response had to be excluded before enrollment. Prior radiation therapyhad to have been completed at least 1 month before treatment, and radiopharmaceuticals could not have been administered within 2 months of treatment. Patients who required systemic corticosteroids for any indication were not eligible. |
| Subudhi et al. (30) | 1) At least 18 years of age, 2) have histologically confirmed prostate carcinoma, with radiographic evidence of metastatic disease, 3) tumor progression while on hormone therapy with castrate serum testosterone (≤1.7 nM or 50 ng/dl) with biopsy-proven viable disease, PSA, and/or radiographic progression according to the Prostate Cancer Clinical Trials Working Group 2, 4) have had a resected prostate cancer mass (primary and/or metastatic site) within 3 months of study entry, 5) adequate hematological, renal, and hepatic function, and 6) an ECOG PS of 0 to 1. |
| Vaishampayan et al. (31) | Patients who did or did not previously take abiraterone acetate were eligible if they developed resistance to Enzalutamide after prior response. Cohorts were composed of pts who had RECIST-measurable (C4) or bone-predominant nonmeasurable (C5) disease. |
| Yu (1) et al. (32) | Molecularly unselected, docetaxel-pretreated mCRPC whose disease progressed within 6 mo before screening. Patients could have received chemotherapy other than docetaxel for mCRPC and second-generation androgen receptoretargeted therapies. |
| Slovin et al. (33) | 1) Diagnosed with mCRPC (rising PSA or progression on scans with a serum testosterone concentration of <50 ng/dl) and the evidence of progression after the discontinuation of anti-androgen therapy who had no more than one prior chemotherapy, 2) had a life expectancy of >12 weeks, 3) an ECOG PS of 0 or 1, and 4) adequate hematologic, hepatic, and renal functions. Adenocarcinoma of the prostate was confirmed histologically, and the extent of disease was documented radiographically by bone scan and computed tomography. Patients with radiation-induced diarrhea within 12 months of study entry or with prior colitis or irritable bowel syndrome were excluded. Other key exclusion criteria were autoimmune disease (except for vitiligo) requiring systemic steroids or immunosuppressive agents, other prior malignancy within 5 years, active infection, bone pain severe enough to require routine narcotic analgesics, and prior treatment with anti-CTLA-4 therapies. |
| Zucali et al. (34) | mCRPC patients |
| Yu (2) et al. (35) | 1) Age more than 18 yr, 2) histologically or cytologically confirmed adenocarcinoma of the prostate without small-cell histology, 3) disease that progressed within 6 mo before screening (PSA progression or radiologic bone/soft tissue progression), 4) EECOG PS of 0 or 1, 5) received 4 wk of treatment with either abiraterone or enzalutamide (but not both) for mCRPC and with treatment failure or intolerance to the drug, 6) no previous chemotherapy, and 7) serum testosterone level <50 ng/dL. |

**Table S4.** Table of p-values for prostate specific antigen response rate in different immune checkpoint inhibitor medication subgroups.

| anti PD-1/PD-L1/CTLA-4 |  |  |  |  |  |  |  |  |  |  |  |  |
| --- | --- | --- | --- | --- | --- | --- | --- | --- | --- | --- | --- | --- |
| *anti PD-1/PD-L1 monotherapy* | **0.007** |  |  |  |  |  |  |  |  |  |  |  |
| *anti CTLA-4 monotherapy* | 0.115 | **0.000** |  |  |  |  |  |  |  |  |  |  |
| *anti PD-1/PD-L1+anti CTLA-4* | 0.302 | **0.003** | 0.880 |  |  |  |  |  |  |  |  |  |
| *anti PD-1/PD-L1+chemotherapy* | **0.000** | **0.000** | **0.000** | **0.000** |  |  |  |  |  |  |  |  |
| *anti PD-1/PD-L1+radiotherapy* | 0.843 | 0.066 | 0.568 | 0.666 | **0.000** |  |  |  |  |  |  |  |
| *anti PD-1/PD-L1+hormon therapy* | **0.000** | **0.000** | **0.000** | **0.000** | 0.058 | **0.000** |  |  |  |  |  |  |
| *anti PD-1/PD-L1+PARP inhibitor* | **0.001** | **0.000** | 0.111 | 0.152 | **0.000** | 0.142 | **0.000** |  |  |  |  |  |
| *anti PD-1/PD-L1+TKI* | **0.000** | **0.000** | **0.035** | 0.053 | **0.001** | 0.059 | **0.046** | 0.425 |  |  |  |  |
| *anti PD-1/PD-L1+anti CD-38* | 0.275 | 0.726 | 0.155 | 0.176 | **0.000** | 0.268 | **0.003** | 0.064 | **0.036** |  |  |  |
| *anti CTLA-4+radiotherapy* | 0.226 | **0.003** | 0.807 | 0.741 | **0.000** | 0.511 | **0.001** | 0.423 | 0.188 | 0.142 |  |  |
| *anti PD-1/PD-L1+anti CTLA-4+hormon therapy* | 0.168 | 0.333 | 0.112 | 0.120 | **0.002** | 0.157 | **0.008** | 0.060 | **0.038** | 0.423 | 0.101 |  |
|  | anti PD-1/PD-L1/CTLA-4 | *anti PD-1/PD-L1 monotherapy* | *anti CTLA-4 monotherapy* | *anti PD-1/PD-L1+anti CTLA-4* | *anti PD-1/PD-L1+chemotherapy* | *anti PD-1/PD-L1+radiotherapy* | *anti PD-1/PD-L1+hormon therapy* | *anti PD-1/PD-L1+PARP inhibitor* | *anti PD-1/PD-L1+TKI* | *anti PD-1/PD-L1+anti CD-38* | *anti CTLA-4+radiotherapy* | *anti PD-1/PD-L1+anti CTLA-4+hormon therapy* |

**Table S5.** Table of p-values for objective-response rate in different immune checkpoint inhibitor medication subgroups.

| anti PD-1/PD-L1/CTLA-4 |  |  |  |  |  |  |  |  |  |  |  |  |
| --- | --- | --- | --- | --- | --- | --- | --- | --- | --- | --- | --- | --- |
| *anti PD-1/PD-L1 monotherapy* | 0.075 |  |  |  |  |  |  |  |  |  |  |  |
| *anti CTLA-4 monotherapy* | NA | NA |  |  |  |  |  |  |  |  |  |  |
| *anti PD-1/PD-L1+anti CTLA-4* | **0.006** | **0.000** | NA |  |  |  |  |  |  |  |  |  |
| *anti PD-1/PD-L1+chemotherapy* | **0.000** | **0.000** | NA | **0.041** |  |  |  |  |  |  |  |  |
| *anti PD-1/PD-L1+radiotherapy* | 0.640 | 0.098 | NA | 0.165 | **0.002** |  |  |  |  |  |  |  |
| *anti PD-1/PD-L1+hormon therapy* | **0.008** | **0.000** | NA | 0.476 | **0.001** | 0.309 |  |  |  |  |  |  |
| *anti PD-1/PD-L1+PARP inhibitor* | 0.913 | 0.094 | NA | **0.020** | **0.000** | 0.715 | **0.040** |  |  |  |  |  |
| *anti PD-1/PD-L1+TKI* | 0.055 | **0.001** | NA | 0.487 | **0.005** | 0.400 | 0.904 | 0.110 |  |  |  |  |
| *anti PD-1/PD-L1+anti CD-38* | 0.977 | 0.629 | NA | 0.425 | 0.129 | 0.841 | 0.544 | 0.954 | 0.573 |  |  |  |
| *anti CTLA-4+radiotherapy* | NA | NA | NA | NA | NA | NA | NA | NA | NA | NA |  |  |
| *anti PD-1/PD-L1+anti CTLA-4+hormon therapy* | 0.336 | 0.464 | NA | 0.153 | **0.049** | 0.292 | 0.194 | 0.329 | 0.203 | 0.353 | NA |  |
|  | anti PD-1/PD-L1/CTLA-4 | *anti PD-1/PD-L1 monotherapy* | *anti CTLA-4 monotherapy* | *anti PD-1/PD-L1+anti CTLA-4* | *anti PD-1/PD-L1+chemotherapy* | *anti PD-1/PD-L1+radiotherapy* | *anti PD-1/PD-L1+hormon therapy* | *anti PD-1/PD-L1+PARP inhibitor* | *anti PD-1/PD-L1+TKI* | *anti PD-1/PD-L1+anti CD-38* | *anti CTLA-4+radiotherapy* | *anti PD-1/PD-L1+anti CTLA-4+hormon therapy* |

**Table S6.** Table of p-values for disease control rate in different immune checkpoint inhibitor medication subgroups.

| anti PD-1/PD-L1/CTLA-4 |  |  |  |  |  |  |  |  |  |  |  |  |
| --- | --- | --- | --- | --- | --- | --- | --- | --- | --- | --- | --- | --- |
| *anti PD-1/PD-L1 monotherapy* | **0.010** |  |  |  |  |  |  |  |  |  |  |  |
| *anti CTLA-4 monotherapy* | 0.096 | **0.004** |  |  |  |  |  |  |  |  |  |  |
| *anti PD-1/PD-L1+anti CTLA-4* | **0.000** | **0.000** | **0.039** |  |  |  |  |  |  |  |  |  |
| *anti PD-1/PD-L1+chemotherapy* | **0.000** | **0.000** | **0.000** | **0.009** |  |  |  |  |  |  |  |  |
| *anti PD-1/PD-L1+radiotherapy* | **0.000** | **0.000** | 0.549 | 0.056 | **0.000** |  |  |  |  |  |  |  |
| *anti PD-1/PD-L1+hormon therapy* | **0.000** | **0.000** | 0.058 | 0.505 | **0.000** | 0.073 |  |  |  |  |  |  |
| *anti PD-1/PD-L1+PARP inhibitor* | **0.000** | **0.000** | 0.074 | 0.482 | **0.000** | 0.107 | 0.920 |  |  |  |  |  |
| *anti PD-1/PD-L1+TKI* | **0.000** | **0.000** | **0.000** | **0.002** | 0.593 | **0.000** | **0.000** | **0.000** |  |  |  |  |
| *anti PD-1/PD-L1+anti CD-38* | **0.021** | **0.001** | 0.343 | 0.670 | 0.055 | 0.531 | 0.897 | 0.925 | **0.027** |  |  |  |
| *anti CTLA-4+radiotherapy* | NA | NA | NA | NA | NA | NA | NA | NA | NA | NA |  |  |
| *anti PD-1/PD-L1+anti CTLA-4+hormon therapy* | NA | NA | NA | NA | NA | NA | NA | NA | NA | NA | NA |  |
|  | anti PD-1/PD-L1/CTLA-4 | *anti PD-1/PD-L1 monotherapy* | *anti CTLA-4 monotherapy* | *anti PD-1/PD-L1+anti CTLA-4* | *anti PD-1/PD-L1+chemotherapy* | *anti PD-1/PD-L1+radiotherapy* | *anti PD-1/PD-L1+hormon therapy* | *anti PD-1/PD-L1+PARP inhibitor* | *anti PD-1/PD-L1+TKI* | *anti PD-1/PD-L1+anti CD-38* | *anti CTLA-4+radiotherapy* | *anti PD-1/PD-L1+anti CTLA-4+hormon therapy* |

**Table S7.** Table of p-values for complete response rate in different immune checkpoint inhibitor medication subgroups.

| anti PD-1/PD-L1/CTLA-4 |  |  |  |  |  |  |  |  |  |  |  |  |
| --- | --- | --- | --- | --- | --- | --- | --- | --- | --- | --- | --- | --- |
| *anti PD-1/PD-L1 monotherapy* | 0.241 |  |  |  |  |  |  |  |  |  |  |  |
| *anti CTLA-4 monotherapy* | 0.052 | **0.004** |  |  |  |  |  |  |  |  |  |  |
| *anti PD-1/PD-L1+anti CTLA-4* | 0.061 | **0.005** | 0.499 |  |  |  |  |  |  |  |  |  |
| *anti PD-1/PD-L1+chemotherapy* | 0.408 | 0.948 | **0.014** | **0.043** |  |  |  |  |  |  |  |  |
| *anti PD-1/PD-L1+radiotherapy* | 0.185 | 0.369 | **0.001** | **0.022** | 0.388 |  |  |  |  |  |  |  |
| *anti PD-1/PD-L1+hormon therapy* | 0.961 | 0.225 | 0.056 | 0.068 | 0.394 | 0.179 |  |  |  |  |  |  |
| *anti PD-1/PD-L1+PARP inhibitor* | 0.056 | 0.195 | **0.000** | **0.001** | 0.213 | NA | 0.053 |  |  |  |  |  |
| *anti PD-1/PD-L1+TKI* | 0.075 | 0.228 | **0.000** | **0.002** | 0.246 | NA | 0.072 | NA |  |  |  |  |
| *anti PD-1/PD-L1+anti CD-38* | 0.604 | 0.725 | 0.197 | 0.365 | 0.735 | NA | 0.599 | NA | NA |  |  |  |
| *anti CTLA-4+radiotherapy* | 0.675 | 0.265 | 0.289 | 0.549 | 0.330 | 0.101 | 0.692 | **0.018** | **0.028** | 0.518 |  |  |
| *anti PD-1/PD-L1+anti CTLA-4+hormon therapy* | NA | NA | NA | NA | NA | NA | NA | NA | NA | NA | NA |  |
|  | anti PD-1/PD-L1/CTLA-4 | *anti PD-1/PD-L1 monotherapy* | *anti CTLA-4 monotherapy* | *anti PD-1/PD-L1+anti CTLA-4* | *anti PD-1/PD-L1+chemotherapy* | *anti PD-1/PD-L1+radiotherapy* | *anti PD-1/PD-L1+hormon therapy* | *anti PD-1/PD-L1+PARP inhibitor* | *anti PD-1/PD-L1+TKI* | *anti PD-1/PD-L1+anti CD-38* | *anti CTLA-4+radiotherapy* | *anti PD-1/PD-L1+anti CTLA-4+hormon therapy* |

| anti PD-1/PD-L1/CTLA-4 |  |  |  |  |  |  |  |  |  |  |  |  |
| --- | --- | --- | --- | --- | --- | --- | --- | --- | --- | --- | --- | --- |
| *anti PD-1/PD-L1 monotherapy* | 0.491 |  |  |  |  |  |  |  |  |  |  |  |
| *anti CTLA-4 monotherapy* | 0.516 | 0.568 |  |  |  |  |  |  |  |  |  |  |
| *anti PD-1/PD-L1+anti CTLA-4* | 0.065 | **0.022** | 0.326 |  |  |  |  |  |  |  |  |  |
| *anti PD-1/PD-L1+chemotherapy* | **0.000** | **0.000** | 0.088 | **0.013** |  |  |  |  |  |  |  |  |
| *anti PD-1/PD-L1+radiotherapy* | 0.094 | **0.032** | 0.352 | 0.837 | **0.003** |  |  |  |  |  |  |  |
| *anti PD-1/PD-L1+hormon therapy* | **0.005** | **0.001** | 0.330 | 0.948 | **0.000** | 0.840 |  |  |  |  |  |  |
| *anti PD-1/PD-L1+PARP inhibitor* | 0.051 | **0.014** | 0.362 | 0.741 | **0.000** | 0.920 | 0.685 |  |  |  |  |  |
| *anti PD-1/PD-L1+TKI* | **0.001** | **0.000** | 0.261 | 0.578 | **0.009** | 0.400 | 0.339 | 0.239 |  |  |  |  |
| *anti PD-1/PD-L1+anti CD-38* | 0.635 | 0.472 | 0.412 | 0.764 | 0.144 | 0.841 | 0.772 | 0.870 | 0.573 |  |  |  |
| *anti CTLA-4+radiotherapy* | 0.203 | 0.263 | NA | 0.057 | **0.001** | 0.071 | 0.057 | 0.075 | **0.029** | 0.112 |  |  |
| *anti PD-1/PD-L1+anti CTLA-4+hormon therapy* | NA | NA | NA | NA | NA | NA | NA | NA | NA | NA | NA |  |
|  | anti PD-1/PD-L1/CTLA-4 | *anti PD-1/PD-L1 monotherapy* | *anti CTLA-4 monotherapy* | *anti PD-1/PD-L1+anti CTLA-4* | *anti PD-1/PD-L1+chemotherapy* | *anti PD-1/PD-L1+radiotherapy* | *anti PD-1/PD-L1+hormon therapy* | *anti PD-1/PD-L1+PARP inhibitor* | *anti PD-1/PD-L1+TKI* | *anti PD-1/PD-L1+anti CD-38* | *anti CTLA-4+radiotherapy* | *anti PD-1/PD-L1+anti CTLA-4+hormon therapy* |

**Table S8.** Table of p-values for partial response rate in different immune checkpoint inhibitor medication subgroups.

| anti PD-1/PD-L1/CTLA-4 |  |  |  |  |  |  |  |  |  |  |  |  |
| --- | --- | --- | --- | --- | --- | --- | --- | --- | --- | --- | --- | --- |
| *anti PD-1/PD-L1 monotherapy* | 0.299 |  |  |  |  |  |  |  |  |  |  |  |
| *anti CTLA-4 monotherapy* | 0.339 | 0.220 |  |  |  |  |  |  |  |  |  |  |
| *anti PD-1/PD-L1+anti CTLA-4* | **0.017** | **0.003** | 0.970 |  |  |  |  |  |  |  |  |  |
| *anti PD-1/PD-L1+chemotherapy* | **0.000** | **0.000** | 0.657 | 0.258 |  |  |  |  |  |  |  |  |
| *anti PD-1/PD-L1+radiotherapy* | 0.251 | 0.072 | 0.612 | 0.306 | **0.018** |  |  |  |  |  |  |  |
| *anti PD-1/PD-L1+hormon therapy* | **0.000** | **0.000** | 0.984 | 0.961 | 0.161 | 0.190 |  |  |  |  |  |  |
| *anti PD-1/PD-L1+PARP inhibitor* | **0.000** | **0.000** | 0.319 | **0.018** | 0.149 | **0.000** | **0.003** |  |  |  |  |  |
| *anti PD-1/PD-L1+TKI* | **0.000** | **0.000** | 0.191 | **0.001** | **0.019** | **0.000** | **0.000** | 0.445 |  |  |  |  |
| *anti PD-1/PD-L1+anti CD-38* | 0.168 | 0.086 | 0.914 | 0.837 | 0.702 | 0.432 | 0.847 | 0.291 | 0.151 |  |  |  |
| *anti CTLA-4+radiotherapy* | **0.048** | **0.014** | 0.940 | 0.840 | 0.514 | 0.306 | 0.849 | 0.105 | **0.028** | 0.955 |  |  |
| *anti PD-1/PD-L1+anti CTLA-4+hormon therapy* | NA | NA | NA | NA | NA | NA | NA | NA | NA | NA | NA |  |
|  | anti PD-1/PD-L1/CTLA-4 | *anti PD-1/PD-L1 monotherapy* | *anti CTLA-4 monotherapy* | *anti PD-1/PD-L1+anti CTLA-4* | *anti PD-1/PD-L1+chemotherapy* | *anti PD-1/PD-L1+radiotherapy* | *anti PD-1/PD-L1+hormon therapy* | *anti PD-1/PD-L1+PARP inhibitor* | *anti PD-1/PD-L1+TKI* | *anti PD-1/PD-L1+anti CD-38* | *anti CTLA-4+radiotherapy* | *anti PD-1/PD-L1+anti CTLA-4+hormon therapy* |

**Table S9.** Table of p-values for stable disease rate in different immune checkpoint inhibitor medication subgroups.

| anti PD-1/PD-L1/CTLA-4 |  |  |  |  |  |  |  |  |  |  |  |  |
| --- | --- | --- | --- | --- | --- | --- | --- | --- | --- | --- | --- | --- |
| *anti PD-1/PD-L1 monotherapy* | 0.667 |  |  |  |  |  |  |  |  |  |  |  |
| *anti CTLA-4 monotherapy* | 0.814 | 0.750 |  |  |  |  |  |  |  |  |  |  |
| *anti PD-1/PD-L1+anti CTLA-4* | 0.217 | 0.149 | 0.827 |  |  |  |  |  |  |  |  |  |
| *anti PD-1/PD-L1+chemotherapy* | **0.000** | **0.000** | 0.101 | **0.004** |  |  |  |  |  |  |  |  |
| *anti PD-1/PD-L1+radiotherapy* | 0.199 | 0.309 | 0.520 | 0.056 | **0.000** |  |  |  |  |  |  |  |
| *anti PD-1/PD-L1+hormon therapy* | 0.247 | 0.146 | 0.957 | 0.672 | **0.000** | 0.054 |  |  |  |  |  |  |
| *anti PD-1/PD-L1+PARP inhibitor* | **0.000** | **0.000** | 0.301 | 0.079 | 0.209 | **0.000** | **0.008** |  |  |  |  |  |
| *anti PD-1/PD-L1+TKI* | **0.000** | **0.000** | 0.098 | **0.002** | 0.974 | **0.000** | **0.000** | 0.186 |  |  |  |  |
| *anti PD-1/PD-L1+anti CD-38* | 0.902 | 0.820 | 0.914 | 0.670 | **0.029** | 0.531 | 0.814 | 0.150 | **0.027** |  |  |  |
| *anti CTLA-4+radiotherapy* | 0.649 | 0.771 | 0.671 | 0.252 | **0.000** | 0.742 | 0.333 | **0.008** | **0.000** | 0.721 |  |  |
| *anti PD-1/PD-L1+anti CTLA-4+hormon therapy* | NA | NA | NA | NA | NA | NA | NA | NA | NA | NA | NA |  |
|  | anti PD-1/PD-L1/CTLA-4 | *anti PD-1/PD-L1 monotherapy* | *anti CTLA-4 monotherapy* | *anti PD-1/PD-L1+anti CTLA-4* | *anti PD-1/PD-L1+chemotherapy* | *anti PD-1/PD-L1+radiotherapy* | *anti PD-1/PD-L1+hormon therapy* | *anti PD-1/PD-L1+PARP inhibitor* | *anti PD-1/PD-L1+TKI* | *anti PD-1/PD-L1+anti CD-38* | *anti CTLA-4+radiotherapy* | *anti PD-1/PD-L1+anti CTLA-4+hormon therapy* |

**Table S10.** Table of p-values for progressive disease rate in different immune checkpoint inhibitor medication subgroups.

**Table S11.** Table of p-values for any grade treatment-related adverse events in different immune checkpoint inhibitor medication subgroups.

| anti PD-1/PD-L1/CTLA-4 |  |  |  |  |  |  |  |  |  |  |  |  |
| --- | --- | --- | --- | --- | --- | --- | --- | --- | --- | --- | --- | --- |
| *anti PD-1/PD-L1 monotherapy* | **0.000** |  |  |  |  |  |  |  |  |  |  |  |
| *anti CTLA-4 monotherapy* | 0.051 | **0.000** |  |  |  |  |  |  |  |  |  |  |
| *anti PD-1/PD-L1+anti CTLA-4* | **0.000** | **0.000** | **0.000** |  |  |  |  |  |  |  |  |  |
| *anti PD-1/PD-L1+chemotherapy* | **0.000** | **0.000** | **0.000** | 0.631 |  |  |  |  |  |  |  |  |
| *anti PD-1/PD-L1+radiotherapy* | 0.053 | **0.001** | 0.125 | 0.426 | 0.200 |  |  |  |  |  |  |  |
| *anti PD-1/PD-L1+hormon therapy* | **0.001** | **0.000** | 0.094 | **0.004** | **0.000** | 0.263 |  |  |  |  |  |  |
| *anti PD-1/PD-L1+PARP inhibitor* | **0.000** | **0.000** | **0.000** | 0.301 | 0.061 | 0.682 | **0.000** |  |  |  |  |  |
| *anti PD-1/PD-L1+TKI* | **0.000** | **0.000** | **0.000** | 0.733 | 0.901 | 0.260 | **0.000** | 0.122 |  |  |  |  |
| *anti PD-1/PD-L1+anti CD-38* | 0.659 | 0.065 | 0.980 | **0.019** | **0.001** | 0.245 | 0.669 | **0.039** | **0.004** |  |  |  |
| *anti CTLA-4+radiotherapy* | **0.006** | **0.000** | **0.043** | 0.122 | **0.015** | 0.716 | 0.203 | 0.243 | **0.037** | 0.271 |  |  |
| *anti PD-1/PD-L1+anti CTLA-4+hormon therapy* | NA | NA | NA | NA | NA | NA | NA | NA | NA | NA | NA |  |
|  | anti PD-1/PD-L1/CTLA-4 | *anti PD-1/PD-L1 monotherapy* | *anti CTLA-4 monotherapy* | *anti PD-1/PD-L1+anti CTLA-4* | *anti PD-1/PD-L1+chemotherapy* | *anti PD-1/PD-L1+radiotherapy* | *anti PD-1/PD-L1+hormon therapy* | *anti PD-1/PD-L1+PARP inhibitor* | *anti PD-1/PD-L1+TKI* | *anti PD-1/PD-L1+anti CD-38* | *anti CTLA-4+radiotherapy* | *anti PD-1/PD-L1+anti CTLA-4+hormon therapy* |

**Table S12.** Table of p-values for ≥ grade 3 treatment-related adverse events in different immune checkpoint inhibitor medication subgroups.

| anti PD-1/PD-L1/CTLA-4 |  |  |  |  |  |  |  |  |  |  |  |  |
| --- | --- | --- | --- | --- | --- | --- | --- | --- | --- | --- | --- | --- |
| *anti PD-1/PD-L1 monotherapy* | **0.000** |  |  |  |  |  |  |  |  |  |  |  |
| *anti CTLA-4 monotherapy* | **0.017** | **0.000** |  |  |  |  |  |  |  |  |  |  |
| *anti PD-1/PD-L1+anti CTLA-4* | **0.002** | **0.000** | 0.054 |  |  |  |  |  |  |  |  |  |
| *anti PD-1/PD-L1+chemotherapy* | **0.000** | **0.000** | **0.046** | 0.758 |  |  |  |  |  |  |  |  |
| *anti PD-1/PD-L1+radiotherapy* | 0.706 | **0.001** | 0.216 | **0.022** | **0.025** |  |  |  |  |  |  |  |
| *anti PD-1/PD-L1+hormon therapy* | 0.494 | **0.000** | **0.007** | **0.001** | **0.000** | 0.915 |  |  |  |  |  |  |
| *anti PD-1/PD-L1+PARP inhibitor* | **0.000** | **0.000** | **0.000** | 0.563 | 0.276 | **0.002** | **0.000** |  |  |  |  |  |
| *anti PD-1/PD-L1+TKI* | **0.000** | **0.000** | **0.000** | 0.240 | 0.092 | **0.001** | **0.000** | 0.415 |  |  |  |  |
| *anti PD-1/PD-L1+anti CD-38* | 0.095 | 0.778 | **0.034** | **0.006** | **0.007** | 0.180 | 0.128 | **0.001** | **0.000** |  |  |  |
| *anti CTLA-4+radiotherapy* | NA | NA | NA | NA | NA | NA | NA | NA | NA | NA |  |  |
| *anti PD-1/PD-L1+anti CTLA-4+hormon therapy* | 0.092 | **0.000** | 0.222 | 0.679 | 0.571 | 0.092 | 0.069 | 0.858 | 0.884 | **0.016** | NA |  |
|  | anti PD-1/PD-L1/CTLA-4 | *anti PD-1/PD-L1 monotherapy* | *anti CTLA-4 monotherapy* | *anti PD-1/PD-L1+anti CTLA-4* | *anti PD-1/PD-L1+chemotherapy* | *anti PD-1/PD-L1+radiotherapy* | *anti PD-1/PD-L1+hormon therapy* | *anti PD-1/PD-L1+PARP inhibitor* | *anti PD-1/PD-L1+TKI* | *anti PD-1/PD-L1+anti CD-38* | *anti CTLA-4+radiotherapy* | *anti PD-1/PD-L1+anti CTLA-4+hormon therapy* |

| anti PD-1/PD-L1/CTLA-4 |  |  |  |  |  |  |  |  |  |  |  |  |
| --- | --- | --- | --- | --- | --- | --- | --- | --- | --- | --- | --- | --- |
| *anti PD-1/PD-L1 monotherapy* | **0.000** |  |  |  |  |  |  |  |  |  |  |  |
| *anti CTLA-4 monotherapy* | **0.000** | **0.000** |  |  |  |  |  |  |  |  |  |  |
| *anti PD-1/PD-L1+anti CTLA-4* | 0.883 | **0.000** | **0.001** |  |  |  |  |  |  |  |  |  |
| *anti PD-1/PD-L1+chemotherapy* | **0.000** | **0.000** | **0.000** | **0.000** |  |  |  |  |  |  |  |  |
| *anti PD-1/PD-L1+radiotherapy* | **0.000** | **0.000** | **0.039** | **0.001** | **0.000** |  |  |  |  |  |  |  |
| *anti PD-1/PD-L1+hormon therapy* | **0.001** | 0.165 | **0.000** | **0.003** | 0.435 | **0.000** |  |  |  |  |  |  |
| *anti PD-1/PD-L1+PARP inhibitor* | **0.004** | 0.342 | **0.000** | **0.010** | 0.450 | **0.000** | 0.911 |  |  |  |  |  |
| *anti PD-1/PD-L1+TKI* | NA | NA | NA | NA | NA | NA | NA | NA |  |  |  |  |
| *anti PD-1/PD-L1+anti CD-38* | NA | NA | NA | NA | NA | NA | NA | NA | NA |  |  |  |
| *anti CTLA-4+radiotherapy* | **0.002** | **0.000** | 0.601 | **0.010** | **0.000** | 0.123 | **0.000** | **0.000** | NA | NA |  |  |
| *anti PD-1/PD-L1+anti CTLA-4+hormon therapy* | NA | NA | NA | NA | NA | NA | NA | NA | NA | NA | NA |  |
|  | anti PD-1/PD-L1/CTLA-4 | *anti PD-1/PD-L1 monotherapy* | *anti CTLA-4 monotherapy* | *anti PD-1/PD-L1+anti CTLA-4* | *anti PD-1/PD-L1+chemotherapy* | *anti PD-1/PD-L1+radiotherapy* | *anti PD-1/PD-L1+hormon therapy* | *anti PD-1/PD-L1+PARP inhibitor* | *anti PD-1/PD-L1+TKI* | *anti PD-1/PD-L1+anti CD-38* | *anti CTLA-4+radiotherapy* | *anti PD-1/PD-L1+anti CTLA-4+hormon therapy* |

**Table S13.** Table of p-values for immune-related adverse events in different immune checkpoint inhibitor medication subgroups.

**Table S14.** Table of p-values for serious adverse events in different immune checkpoint inhibitor medication subgroups.

| anti PD-1/PD-L1/CTLA-4 |  |  |  |  |  |  |  |  |  |  |  |  |
| --- | --- | --- | --- | --- | --- | --- | --- | --- | --- | --- | --- | --- |
| *anti PD-1/PD-L1 monotherapy* | **0.001** |  |  |  |  |  |  |  |  |  |  |  |
| *anti CTLA-4 monotherapy* | 0.388 | **0.000** |  |  |  |  |  |  |  |  |  |  |
| *anti PD-1/PD-L1+anti CTLA-4* | NA | NA | NA |  |  |  |  |  |  |  |  |  |
| *anti PD-1/PD-L1+chemotherapy* | 0.164 | **0.009** | **0.044** | NA |  |  |  |  |  |  |  |  |
| *anti PD-1/PD-L1+radiotherapy* | 0.088 | 0.140 | **0.042** | NA | 0.338 |  |  |  |  |  |  |  |
| *anti PD-1/PD-L1+hormon therapy* | **0.000** | 0.247 | **0.000** | NA | **0.001** | 0.444 |  |  |  |  |  |  |
| *anti PD-1/PD-L1+PARP inhibitor* | 0.155 | **0.012** | **0.045** | NA | 0.919 | 0.377 | **0.003** |  |  |  |  |  |
| *anti PD-1/PD-L1+TKI* | NA | NA | NA | NA | NA | NA | NA | NA |  |  |  |  |
| *anti PD-1/PD-L1+anti CD-38* | 0.060 | 0.536 | **0.035** | NA | 0.174 | 0.543 | 0.847 | 0.191 | NA |  |  |  |
| *anti CTLA-4+radiotherapy* | NA | NA | NA | NA | NA | NA | NA | NA | NA | NA |  |  |
| *anti PD-1/PD-L1+anti CTLA-4+hormon therapy* | NA | NA | NA | NA | NA | NA | NA | NA | NA | NA | NA |  |
|  | anti PD-1/PD-L1/CTLA-4 | *anti PD-1/PD-L1 monotherapy* | *anti CTLA-4 monotherapy* | *anti PD-1/PD-L1+anti CTLA-4* | *anti PD-1/PD-L1+chemotherapy* | *anti PD-1/PD-L1+radiotherapy* | *anti PD-1/PD-L1+hormon therapy* | *anti PD-1/PD-L1+PARP inhibitor* | *anti PD-1/PD-L1+TKI* | *anti PD-1/PD-L1+anti CD-38* | *anti CTLA-4+radiotherapy* | *anti PD-1/PD-L1+anti CTLA-4+hormon therapy* |

| anti PD-1/PD-L1/CTLA-4 |  |  |  |  |  |  |  |  |  |  |  |  |
| --- | --- | --- | --- | --- | --- | --- | --- | --- | --- | --- | --- | --- |
| *anti PD-1/PD-L1 monotherapy* | **0.000** |  |  |  |  |  |  |  |  |  |  |  |
| *anti CTLA-4 monotherapy* | **0.000** | **0.000** |  |  |  |  |  |  |  |  |  |  |
| *anti PD-1/PD-L1+anti CTLA-4* | 0.157 | **0.002** | **0.014** |  |  |  |  |  |  |  |  |  |
| *anti PD-1/PD-L1+chemotherapy* | 0.411 | **0.000** | 0.106 | 0.097 |  |  |  |  |  |  |  |  |
| *anti PD-1/PD-L1+radiotherapy* | **0.001** | 0.132 | **0.000** | 0.223 | **0.000** |  |  |  |  |  |  |  |
| *anti PD-1/PD-L1+hormon therapy* | **0.000** | **0.000** | **0.000** | 0.871 | **0.000** | 0.134 |  |  |  |  |  |  |
| *anti PD-1/PD-L1+PARP inhibitor* | 0.956 | **0.000** | **0.024** | 0.176 | 0.573 | **0.002** | **0.001** |  |  |  |  |  |
| *anti PD-1/PD-L1+TKI* | 0.294 | **0.000** | **0.003** | 0.423 | 0.157 | **0.014** | 0.066 | 0.374 |  |  |  |  |
| *anti PD-1/PD-L1+anti CD-38* | NA | NA | NA | NA | NA | NA | NA | NA | NA |  |  |  |
| *anti CTLA-4+radiotherapy* | 0.658 | **0.000** | 0.189 | 0.138 | 0.900 | **0.001** | **0.003** | 0.737 | 0.280 | NA |  |  |
| *anti PD-1/PD-L1+anti CTLA-4+hormon therapy* | NA | NA | NA | NA | NA | NA | NA | NA | NA | NA | NA |  |
|  | anti PD-1/PD-L1/CTLA-4 | *anti PD-1/PD-L1 monotherapy* | *anti CTLA-4 monotherapy* | *anti PD-1/PD-L1+anti CTLA-4* | *anti PD-1/PD-L1+chemotherapy* | *anti PD-1/PD-L1+radiotherapy* | *anti PD-1/PD-L1+hormon therapy* | *anti PD-1/PD-L1+PARP inhibitor* | *anti PD-1/PD-L1+TKI* | *anti PD-1/PD-L1+anti CD-38* | *anti CTLA-4+radiotherapy* | *anti PD-1/PD-L1+anti CTLA-4+hormon therapy* |

**Table S15.** Table of p-values for adverse events led to treatment discontinuation in different immune checkpoint inhibitor medication subgroups.

| anti PD-1/PD-L1/CTLA-4 |  |  |  |  |  |  |  |  |  |  |  |  |
| --- | --- | --- | --- | --- | --- | --- | --- | --- | --- | --- | --- | --- |
| *anti PD-1/PD-L1 monotherapy* | 0.315 |  |  |  |  |  |  |  |  |  |  |  |
| *anti CTLA-4 monotherapy* | 0.621 | 0.212 |  |  |  |  |  |  |  |  |  |  |
| *anti PD-1/PD-L1+anti CTLA-4* | 0.997 | 0.452 | 0.813 |  |  |  |  |  |  |  |  |  |
| *anti PD-1/PD-L1+chemotherapy* | 0.128 | 0.051 | 0.267 | 0.355 |  |  |  |  |  |  |  |  |
| *anti PD-1/PD-L1+radiotherapy* | **0.049** | **0.016** | 0.109 | 0.176 | 0.568 |  |  |  |  |  |  |  |
| *anti PD-1/PD-L1+hormon therapy* | 0.570 | 0.197 | 0.927 | 0.777 | 0.313 | 0.130 |  |  |  |  |  |  |
| *anti PD-1/PD-L1+PARP inhibitor* | 0.809 | 0.518 | 0.601 | 0.866 | 0.198 | 0.082 | 0.566 |  |  |  |  |  |
| *anti PD-1/PD-L1+TKI* | 0.618 | 0.853 | 0.498 | 0.674 | 0.217 | 0.103 | 0.476 | 0.757 |  |  |  |  |
| *anti PD-1/PD-L1+anti CD-38* | 0.581 | 0.703 | 0.545 | 0.581 | 0.419 | 0.320 | 0.537 | 0.609 | 0.669 |  |  |  |
| *anti CTLA-4+radiotherapy* | 0.282 | 0.457 | 0.239 | 0.283 | 0.116 | 0.054 | 0.230 | 0.320 | 0.405 | NA |  |  |
| *anti PD-1/PD-L1+anti CTLA-4+hormon therapy* | 0.662 | 0.763 | 0.632 | 0.662 | 0.522 | 0.431 | 0.626 | 0.686 | 0.735 | NA | NA |  |
|  | anti PD-1/PD-L1/CTLA-4 | *anti PD-1/PD-L1 monotherapy* | *anti CTLA-4 monotherapy* | *anti PD-1/PD-L1+anti CTLA-4* | *anti PD-1/PD-L1+chemotherapy* | *anti PD-1/PD-L1+radiotherapy* | *anti PD-1/PD-L1+hormon therapy* | *anti PD-1/PD-L1+PARP inhibitor* | *anti PD-1/PD-L1+TKI* | *anti PD-1/PD-L1+anti CD-38* | *anti CTLA-4+radiotherapy* | *anti PD-1/PD-L1+anti CTLA-4+hormon therapy* |

**Table S16.** Table of p-values for adverse events led to death in different immune checkpoint inhibitor medication subgroups.

1. Agarwal N, McGregor B, Maughan BL, Dorff TB, Kelly W, Fang B, et al. Cabozantinib in combination with atezolizumab in patients with metastatic castration-resistant prostate cancer: results from an expansion cohort of a multicentre, open-label, phase 1b trial (COSMIC-021). Lancet Oncol. 2022;23(7):899-909.

2. Alva AS, Li J, Chou J, Reimers MA, McKay RR, Zhang J, et al. Phase 2 trial of immunotherapy in tumors with CDK12 inactivation (IMPACT): Results from cohort A of patients (pts) with metastatic castration resistant prostate cancer (mCRPC) receiving dual immune checkpoint inhibition (ICI). Journal of Clinical Oncology. 2022;40(6 SUPPL).

3. Antonarakis ES, Piulats JM, Gross-Goupil M, Goh J, Ojamaa K, Hoimes CJ, et al. Pembrolizumab for Treatment-Refractory Metastatic Castration-Resistant Prostate Cancer: Multicohort, Open-Label Phase II KEYNOTE-199 Study. Journal of Clinical Oncology. 2020;38(5).

4. Antonarakis ES, Piulats JM, Gross-Goupil M, Goh JC, Vaishampayan UN, De Wit R, et al. Pembrolizumab (pembro) monotherapy for docetaxel-pretreated metastatic castration-resistant prostate cancer (mCRPC): Updated analyses with 4 years of follow-up from cohorts 1-3 of the KEYNOTE-199 study. Annals of Oncology. 2021;32:S651-S2.

5. Boudadi K, Suzman DL, Anagnostou V, Fu W, Luber B, Wang H, et al. Ipilimumab plus nivolumab and DNA-repair defects in AR-V7-expressing metastatic prostate cancer. Oncotarget. 2018;9(47):28561-71.

6. Brown LC, Halabi S, Somarelli JA, Humeniuk M, Wu Y, Oyekunle T, et al. A phase 2 trial of avelumab in men with aggressive-variant or neuroendocrine prostate cancer. Prostate Cancer Prostatic Dis. 2022:1-8.

7. Fizazi K, Retz M, Petrylak DP, Goh JC, Perez-Gracia J, Lacombe L, et al. Nivolumab plus rucaparib for metastatic castration-resistant prostate cancer: results from the phase 2 CheckMate 9KD trial. J Immunother Cancer. 2022;10(8).

8. Fizazi K, González Mella P, Castellano D, Minatta JN, Rezazadeh Kalebasty A, Shaffer D, et al. Nivolumab plus docetaxel in patients with chemotherapy-naïve metastatic castration-resistant prostate cancer: results from the phase II CheckMate 9KD trial. European Journal of Cancer. 2022;160:61-71.

9. Fakhrejahani F, Madan RA, Dahut WL, Karzai F, Cordes LM, Schlom J, et al. Avelumab in metastatic castration-resistant prostate cancer (mCRPC). Journal of Clinical Oncology. 2017;35(6).

10. Graff JN, Stein MN, Surana R, Al Rabadi L, Liu ER, Fong L, et al. Phase II Study of Ipilimumab in Men With Metastatic Prostate Cancer With an Incomplete Response to Androgen Deprivation Therapy. Frontiers in Oncology. 2020;10.

11. Fong L, Morris MJ, Sartor O, Higano CS, Pagliaro L, Alva A, et al. A phase Ib study of atezolizumab with radium-223 dichloride in men with metastatic castration-resistant prostate cancer. Clinical Cancer Research. 2021;27(17):4746-56.

12. Graff JN, Beer TM, Alumkal JJ, Slottke RE, Redmond WL, Thomas GV, et al. A phase II single-arm study of pembrolizumab with enzalutamide in men with metastatic castration-resistant prostate cancer progressing on enzalutamide alone. J Immunother Cancer. 2020;8(2).

13. Hansen AR, Massard C, Ott PA, Haas NB, Lopez JS, Ejadi S, et al. Pembrolizumab for advanced prostate adenocarcinoma: findings of the KEYNOTE-028 study. Ann Oncol. 2018;29(8):1807-13.

14. Hotte SJ, Winquist E, Chi KN, Ellard SL, Sridhar S, Emmenegger U, et al. CCTG IND 232: A phase II study of durvalumab with or without tremelimumab in patients with metastatic castration resistant prostate cancer (mCRPC). Annals of Oncology. 2019;30:v885.

15. Gurney H. Multicohort Phase 1b/2 Study of Pembrolizumab Combination Therapies in Patients With Metastatic Castration-Resistant Prostate Cancer: Updated Results From KEYNOTE 365 Cohorts A-C. Asia-Pacific Journal of Clinical Oncology. 2021;17(SUPPL 7):59-60.

16. Karzai F, Vanderweele D, Madan RA, Owens H, Cordes LM, Hankin A, et al. Activity of durvalumab plus olaparib in metastatic castration-resistant prostate cancer in men with and without DNA damage repair mutations 11 Medical and Health Sciences 1112 Oncology and Carcinogenesis. Journal for ImmunoTherapy of Cancer. 2018;6(1).

17. Kwan EM, Spain L, Anton A, Gan CL, Garrett L, Chang D, et al. Avelumab Combined with Stereotactic Ablative Body Radiotherapy in Metastatic Castration-resistant Prostate Cancer: The Phase 2 ICE-PAC Clinical Trial. European Urology. 2022;81(3):253-62.

18. Kwon ED, Drake CG, Scher HI, Fizazi K, Bossi A, Van den Eertwegh AJM, et al. Ipilimumab versus placebo after radiotherapy in patients with metastatic castration-resistant prostate cancer that had progressed after docetaxel chemotherapy (CA184-043): A multicentre, randomised, double-blind, phase 3 trial. The Lancet Oncology. 2014;15(7):700-12.

19. Layton JL, Manogue C, Light M, Jaeger E, Cotogno P, Ledet EM, et al. PD-L1 inhibition with avelumab plus abiraterone acetate or enzalutamide in African Americans with metastatic castrate-resistant prostate cancer (mCRPC). Journal of Clinical Oncology. 2021;39(6 SUPPL).

20. Markowski MC, Taplin ME, Aggarwal RR, Wang H, Lalji A, Paller CJ, et al. COMBAT-CRPC: Concurrent administration of bipolar androgen therapy (BAT) and nivolumab in men with metastatic castration-resistant prostate cancer (mCRPC). Journal of Clinical Oncology. 2021;39(15 SUPPL).

21. Mourey L, Conter HJ, Shore N, Berry WR, Fong PC, Piulats JM, et al. Pembrolizumab (pembro) plus enzalutamide (enza) in patients with abiraterone acetate (abi)-pretreated metastatic castration-resistant prostate cancer (mCRPC): KEYNOTE-365 Cohort C update. Annals of Oncology. 2020;31:S516-S7.

22. Petrylak DP, Loriot Y, Shaffer DR, Braiteh F, Powderly J, Harshman LC, et al. Safety and Clinical Activity of Atezolizumab in Patients with Metastatic Castration-Resistant Prostate Cancer: A Phase I Study. Clin Cancer Res. 2021;27(12):3360-9.

23. Piulats J, Ferrario C, Linch M, Stoeckle M, Laguerre B, Arranz J, et al. 351 KEYNOTE-365 cohort D: pembrolizumab plus abiraterone acetate and prednisone in patients with chemotherapy-naive metastatic castration-resistant prostate cancer (mCRPC). BMJ Specialist Journals; 2021.

24. Beer TM, Kwon ED, Drake CG, Fizazi K, Logothetis C, Gravis G, et al. Randomized, double-blind, phase III trial of ipilimumab versus placebo in asymptomatic or minimally symptomatic patients with metastatic chemotherapy-naive castration-resistant prostate cancer. Journal of Clinical Oncology. 2017;35(1):40-7.

25. Powles T, Yuen KC, Gillessen S, Kadel EE, Rathkopf D, Matsubara N, et al. Atezolizumab with enzalutamide versus enzalutamide alone in metastatic castration-resistant prostate cancer: a randomized phase 3 trial. Nature Medicine. 2022;28(1):144-+.

26. Rodriguez-Vida A, Maroto Rey JP, Font Pous A, Martin C, Mellado B, Corbera Lloret A, et al. Safety and efficacy of avelumab plus carboplatin in patients with metastatic castration resistant prostate cancer in an open-label phase Ib study. Annals of Oncology. 2021;32:S665.

27. Sharma P, Pachynski RK, Narayan V, Flechon A, Gravis G, Galsky MD, et al. Nivolumab Plus Ipilimumab for Metastatic Castration-Resistant Prostate Cancer: Preliminary Analysis of Patients in the CheckMate 650 Trial. Cancer Cell. 2020;38(4):489-+.

28. Shenderov E, Boudadi K, Fu W, Wang H, Sullivan R, Jordan A, et al. Nivolumab plus ipilimumab, with or without enzalutamide, in AR-V7-expressing metastatic castration-resistant prostate cancer: A phase-2 nonrandomized clinical trial. Prostate. 2021;81(6):326-38.

29. Small EJ, Tchekmedyian NS, Rini BI, Fong L, Lowy I, Allison JP. A pilot trial of CTLA-4 blockade with human anti-CTLA-4 in patients with hormone-refractory prostate cancer. Clin Cancer Res. 2007;13(6):1810-5.

30. Subudhi SK, Vence L, Zhao H, Blando J, Yadav SS, Xiong Q, et al. Neoantigen responses, immune correlates, and favorable outcomes after ipilimumab treatment of patients with prostate cancer. Science Translational Medicine. 2020;12(537).

31. Vaishampayan UN, Elliott T, Omlin AG, Graff JN, Hoimes CJ, Tagawa ST, et al. Phase II study of pembrolizumab (pembro) plus enzalutamide for enzalutamide (enza)-resistant metastatic castration-resistant prostate cancer (mCRPC): Cohorts (C) 4 and 5 update from KEYNOTE-199. Annals of Oncology. 2020;31:S1330.

32. Yu E, Piulats JM, Gravis G, Fong PCC, Todenhöfer T, Laguerre B, et al. Pembrolizumab (pembro) plus olaparib in patients with docetaxel-pretreated metastatic castration-resistant prostate cancer (mCRPC): Update of KEYNOTE-365 cohort A with a minimum of 11 months of follow-up for all patients. Annals of Oncology. 2021;32:S652-S3.

33. Slovin SF, Higano CS, Hamid O, Tejwani S, Harzstark A, Alumkal JJ, et al. Ipilimumab alone or in combination with radiotherapy in metastatic castration-resistant prostate cancer: results from an open-label, multicenter phase I/II study. Ann Oncol. 2013;24(7):1813-21.

34. Zucali PA, Lin CC, Carthon BC, Bauer TM, Tucci M, Italiano A, et al. Targeting CD38 and PD-1 with isatuximab plus cemiplimab in patients with advanced solid malignancies: results from a phase I/II open-label, multicenter study. Journal for Immunotherapy of Cancer. 2022;10(1).

35. Yu EY, Kolinsky MP, Berry WR, Retz M, Mourey L, Piulats JM, et al. Pembrolizumab Plus Docetaxel and Prednisone in Patients with Metastatic Castration-resistant Prostate Cancer: Long-term Results from the Phase 1b/2 KEYNOTE-365 Cohort B Study. Eur Urol. 2022;82(1):22-30.
